# Remodelling of microRNAs in colorectal cancer by hypoxia alters metabolism profiles and 5-fluorouracil resistance

**DOI:** 10.1093/hmg/ddx059

**Published:** 2017-02-16

**Authors:** Anke Nijhuis, Hannah Thompson, Julie Adam, Alexandra Parker, Luke Gammon, Amy Lewis, Jacob G. Bundy, Tomoyoshi Soga, Aisha Jalaly, David Propper, Rosemary Jeffery, Nirosha Suraweera, Sarah McDonald, Mohamed A. Thaha, Roger Feakins, Robert Lowe, Cleo L. Bishop, Andrew Silver

**Affiliations:** 1 Centre for Genomics and Child Health, Blizard Institute, Barts and The London School of Medicine and Dentistry, London E1 2AT, UK; 2 Radcliffe Department of Medicine, OCDEM, University of Oxford, Oxford OX3 7LJ, UK; 3 Centre for Cell Biology and Cutaneous Research, Blizard Institute, Barts and The London School of Medicine and Dentistry, London E1 2AT, UK; 4 Department of Surgery and Cancer, Imperial College London, Sir Alexander Fleming Building, London SW7 2AZ, UK; 5 Institute for Advanced Biosciences, Keio University, Tsuruoka, Yamagata 997-0052, Japan; 6 Department of Medical Oncology, St Bartholomew's Hospital, Gloucester House, Little Britain, London EC1A 7BE, UK; 7 Academic Surgical Unit, The Royal London Hospital, Whitechapel, London E1 1BB, UK; 8 Department of Histopathology, Royal London Hospital, Whitechapel, London, UK

## Abstract

Solid tumours have oxygen gradients and areas of near and almost total anoxia. Hypoxia reduces sensitivity to 5-fluorouracil (5-FU)-chemotherapy for colorectal cancer (CRC). MicroRNAs (miRNAs) are hypoxia sensors and were altered consistently in six CRC cell lines (colon cancer: DLD-1, HCT116 and HT29; rectal cancer: HT55, SW837 and VACO4S) maintained in hypoxia (1 and 0.2% oxygen) compared with normoxia (20.9%). CRC cell lines also showed altered amino acid metabolism in hypoxia and hypoxia-responsive miRNAs were predicted to target genes in four metabolism pathways: beta-alanine; valine, leucine, iso-leucine; aminoacyl-tRNA; and alanine, aspartate, glutamate. MiR-210 was increased in hypoxic areas of CRC tissues and hypoxia-responsive miR-21 and miR-30d, but not miR-210, were significantly increased in 5-FU resistant CRCs. Treatment with miR-21 and miR-30d antagonists sensitized hypoxic CRC cells to 5-FU. Our data highlight the complexity and tumour heterogeneity caused by hypoxia. MiR-210 as a hypoxic biomarker, and the targeting of miR-21 and miR-30d and/or the amino acid metabolism pathways may offer translational opportunities.

## Introduction

Recurrence of colorectal cancer (CRC) following surgery and chemotherapy occurs in almost 50% of patients and is driven, in part, by the acquisition of resistance to chemo- and radiotherapy (1). Low oxygen tension (hypoxia) within the tumour microenvironment is a consistent feature of solid tumours. Hypoxia is associated with a poorer prognosis for many cancers, including breast (2), cervix (3), head and neck (4), and CRC (5). This is probably because of hypoxic areas being more resistant to chemo- and radiotherapy (6,7). Understanding the relationship between the hypoxic microenvironment and how the tumour cells therein adapt to survive and proliferate is critical in developing better therapies that circumvent mechanisms of resistance.

Hypoxia inducible factor-1 alpha (HIF-1α) is the key regulator of cellular response to hypoxia and can act as an experimental biomarker of hypoxia. Although a few reports have shown a correlation between HIF-1α and poor prognosis (8,9), accurately detecting hypoxia is challenging (10) because of tumour heterogeneity, the short half-life of the protein and technical issues associated with immuno-histochemical (IHC) detection in formalin-fixed paraffin-embedded (FFPE) sections. Moreover, indirect assessment of hypoxia using endogenous markers such as HIFs are inherently different from direct measures of oxygen partial pressure, which themselves present technical difficulties and shortcoming when assessing tumours *in vivo* or *exvivo*. Potentially any HIF-1α responsive protein could act as a surrogate marker of tumour hypoxia and most attention has focused on carbonic anhydrase 9 (CAIX). CAIX has been considered an endogenous marker of hypoxia and many solid tumours overexpress CAIX (11). The relationship between the expression of CAIX and patient outcome has been investigated extensively [reviewed in (12)], although expression of CAIX as a consequence of hypoxia is often implied rather than proved.

MicroRNAs (miRNAs) are non-coding RNA molecules that regulate mRNAs (13–15). Their expression is altered in tumours compared with normal tissue and both oncogenic and tumour suppressive roles have been identified for them (16). In addition, miRNAs are differentially expressed and their biogenesis altered in the hypoxic regions of tumours (17–22). Hypoxia-responsive miRNAs have been identified by comparing cancer cell lines maintained in hypoxia against controls sustained in normoxia. Furthermore, hypoxic signatures of miRNA expression are known for a number of tumour types including glioblastoma and bladder cancer (23,24). However, *in vitro* studies have often used only a few cancer-specific lines and corroborating *in vivo* data remains very limited. A larger-scale identification of miRNA expression under hypoxia in an extensive panel of CRC cell lines with supporting *in vivo* data is currently lacking.

The hypoxamir-210 is consistently upregulated in hypoxia across a number of cancer types (25). Many targets of miR-210 regulate cell cycle, differentiation, apoptosis, translation, transcription, metabolism and migration (25). Using matched fresh frozen CRCs and control tissue, Qu *et al.* showed that miR-210 was frequently up-regulated in the cancer (26). Although the degree of hypoxia was not assessed in resected tissues, miR-210 expression correlated significantly with large tumour size, lymph node metastasis, advanced clinical stage and poor prognosis (26). Experimental over-expression of miR-210 promoted migration and invasion in transwell experiments using the HT-29 and SW480 CRC lines *in vitro* (26)*.* However, whether hypoxia modulated these responses was not investigated.

The chemotherapeutic drug 5-fluorouracil (5-FU) has for decades been the standard first-line treatment for CRC (27). Although treatment options have broadened with the availability of therapies combined with 5-FU, tumour resistance remains a major challenge in the treatment of advanced CRC (28,29). The altered profile of miRNAs induced by 5-FU has been determined in CRC cell lines maintained in normoxia (30), but the role of hypoxia on miRNA modulation of chemosensitivity has not been investigated extensively. In particular, it is unclear whether expression of individual miRNAs is simply a consequence of hypoxia, or whether hypoxia-responsive miRNAs are of critical biological importance. For example, metabolic reprogramming is essential for cancer cell survival, with and without the additional stress of surviving exposure to chemotherapy drugs, in both normoxic and hypoxic environments. In the cancer cell, miRNAs regulate key metabolic transporters and enzymes (31), and so a role for hypoxia-responsive miRNAs is possible and requires investigation.

Clearly, the identification of markers of hypoxia with clinical/biomarker utility and an understanding of their role in tumorigenesis would be welcomed. Moreover, a better understanding of the molecular events involved in tumour adaptation to hypoxia and its consequences with respect to treatment response will help to improve survival outcome for CRC patients. Whilst *in vitro* experimental studies commonly use oxygen tensions in the region of 0.8–1.0%, there is a paucity of data from studies that consider conditions of more severe hypoxia. Yet large gradients of oxygen tension, including areas of near anoxia (0.1% O_2_) and almost total anoxia have been recorded in tumours and in a spheroid model (32–36).

Here, we investigated miRNA expression and metabolite profiles in a panel of six CRC cell lines under hypoxic (1%) and severe hypoxic (0.2%) conditions. Following validation, *in situ* hybridization demonstrated the up-regulation of miRNAs in human CRC tumours. Hypoxia-responsive miRNAs were upregulated in 5-FU resistant CRC tumours and miRNA inhibition *in vitro* could sensitise CRC cells to 5-FU in hypoxia. Finally, our studies indicate that changes in the metabolic profile affected by hypoxia in the CRC cell line panel are associated with altered amino acid metabolism and may be linked with hypoxia-responsive miRNAs.

## Results

### MiRNA array revealed hypoxia-responsive miRNAs in CRC cell lines

**Figure 1:**
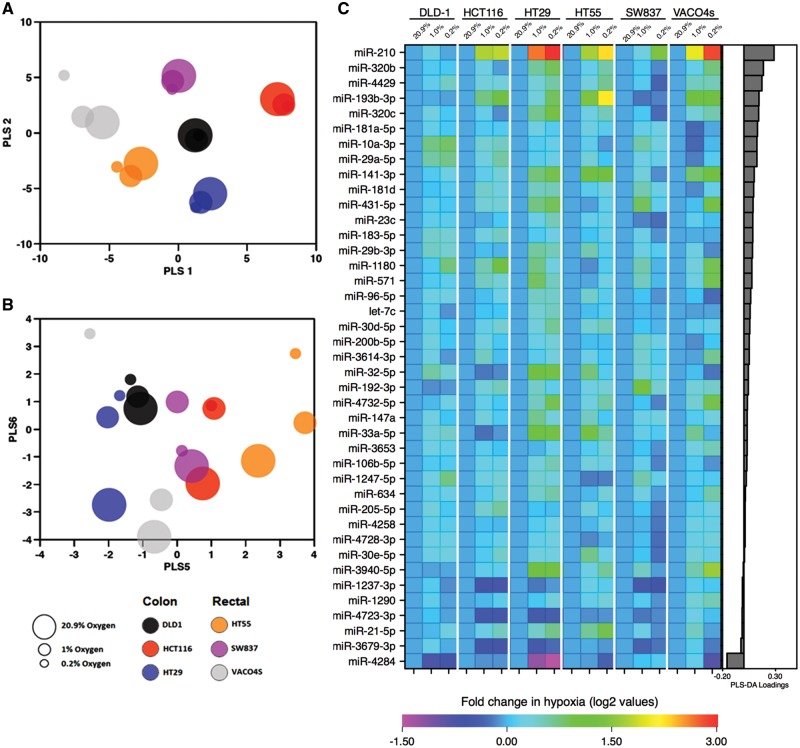
Altered miRNA expression profile under hypoxic conditions. Log-transformed fold change expression of significant miRNAs between normoxia (20.9%) and both low oxygen conditions (1 and 0.2%) in six CRC cell lines for 48 h. (**A**) Data were subjected to PLS-DA, which was supervised according to cell line and unsupervised to oxygen tension. Observation of the first two axes (PLS 1 and PLS 2) demonstrated a clear separation of the miRNA data between the different cell lines, suggesting the main variation in the data is because of the differential expression of the miRNAs between the cell lines; further cell line differences were observed on additional PLS axes and are not shown here (six axes were fitted on the basis of cross-validation). (**B**) A separation of the data according to oxygen tension was observed along PLS axis 6 in five of the six cell lines (excluding SW837). This model indicates an existence of differential expression of miRNA between cells maintained under the three different oxygen conditions. (**C**) miRNAs that are significantly altered under hypoxia were subsequently identified by linear regression multivariate analysis. A heatmap of log-transformed fold changes between the different oxygen tensions normalized to 20.9% is shown. The miRNAs are ranked according to PLS-DA loadings.

MiRNAs are key players in many cellular processes, including pathways that respond to changes in the cell microenvironment such as hypoxia. CRC cell lines are representative of the main subtypes of primary tumours at the genomic level and effective tools for investigating CRC biology (37). Firstly, we aimed to identify miRNAs expressed commonly in three colon cancer cell lines (DLD-1, HCT116, HT29) and three rectal cancer cell lines (HT55, SW837, VACO4s) cultured for 48 h in normoxic (20.9% oxygen) and two hypoxic tensions (1 and 0.2% oxygen). Following global miRNA expression profiling (see Material and Methods), log-transformed expression data of the six cell lines were subjected to partial least squares discriminant analysis (PLS-DA) modelling (see Materials and Methods, Supplementary Material, Tables S1 and S2). Here, the analysis was supervised with regard to cell line and unsupervised in regard to oxygen tension. Unsurprisingly, there were clear differences between the six cell lines (Fig. 1A). However, PLS axis 6 showed an additional (and unsupervised) separation according to oxygen tension for five out of the six cell lines (Fig. 1B). Even though the cell line is by far the greatest source of variance in the data, there is a miRNA signature of hypoxia once the effects of cell line have been allowed for.

Given this, we used (univariate) linear models for each miRNA in turn against each cell line (treated as a categorical variable) and oxygen tension (treated as an ordinal variable). A total of 41 miRNAs (2.2%, 41/1896) were significantly (*P* < 0.05) associated with oxygen tension (see Supplementary Material, Table S3). Fold change in expression between normoxia and both hypoxic tensions (20.9 versus 1% and 20.9 versus 0.2%) were calculated. Log-transformed fold changes for the 41 altered miRNAs are ranked according to their PLS-DA loadings from the PLS axis 6 and illustrated in a heatmap (Fig. 1C). The most significantly altered miRNA across the six cell lines was miR-210, which accords with previous reports that miR-210 is consistently up-regulated following hypoxia in cancer cell lines (38). PLS-DA loadings of axis 6 demonstrated high loading scores (see Supplementary Material, Fig. S1), also showing that this miRNA has a large impact on the separation of the miRNA data between the three oxygen conditions.

**Figure 2:**
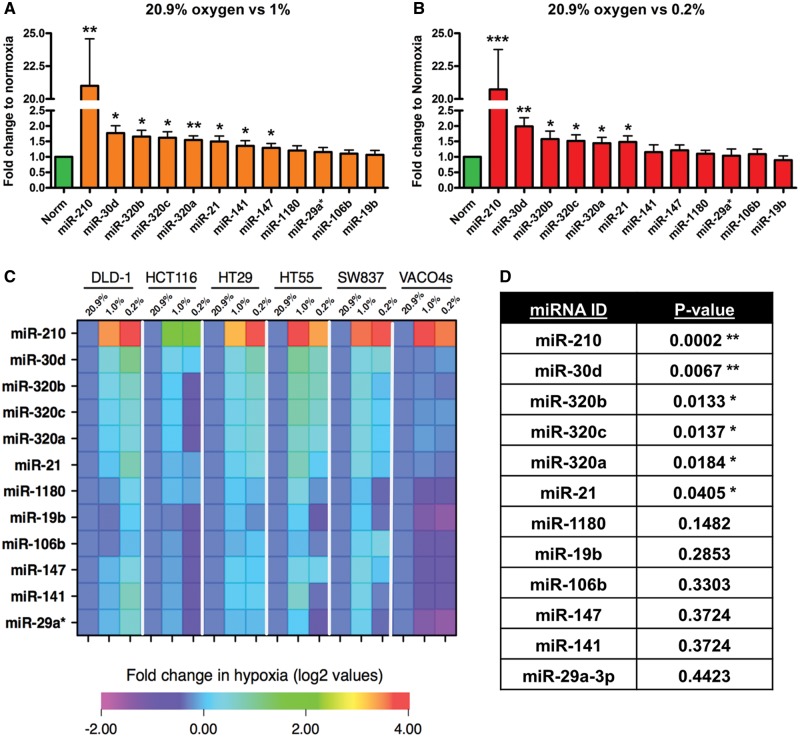
Validation of hypoxia-responsive miRNAs by qRT-PCR in six CRC cell lines. (**A** and **B**) Fold change expression of candidate miRNAs in normoxia (20.9%) and low oxygen tensions (1 and 0.2% oxygen) in six CRC cell lines combined. (**C**) Heatmap of fold change (log 2) in miRNA expression between the three oxygen conditions normalized to 20.9% oxygen (n = 3). (**D**) Multivariate analysis (linear regression model) identified a significant up-regulation of 6 of the 12 miRNAs between in hypoxia. The heatmap is ranked according to *P*-values obtained from the multivariate analysis. Asterisks indicate significantly altered (*P* < 0.05) miRNAs. Bars represent mean values with SEM calculated from three individual experiments. *P < 0.05, **P < 0.01 and ***P < 0.001.

Next, we chose a subset of 12/41 candidate miRNAs that were upregulated for validation (miR-19b; -106b; -141; -147; -1180; -21; -29a*; -210; -30d; -320a; -320b; 320c) based on previous reports linking the miRNA to hypoxia or therapy resistance and/or on statistical significance values, and the view that upregulated miRNAs rather than down regulated might have better utility as biomarkers (21,38–43). Validation was performed by qRT-PCR on independently generated samples, which confirmed the marked increase in miR-210 under both hypoxic conditions (Fig. 2A and B). A further 12 miRNAs were confirmed as significantly altered under the hypoxic oxygen tensions as compared with normoxia; seven were significant for 20.9 versus 1% oxygen and five for 20.9 versus 0.2% oxygen, respectively (Fig. 2A and C). These miRNAs were ranked according to their *P*-value calculated from the linear regression (Fig. 2D) and overall, the up-regulation of six miRNAs (miR-21, -210, -30d, -320a, b and c) was validated under both low oxygen tensions in CRC *in vitro* and miR-210 again found to be the most significant.

### A role for cellular metabolism in hypoxia in CRC cell lines

**Figure 3:**
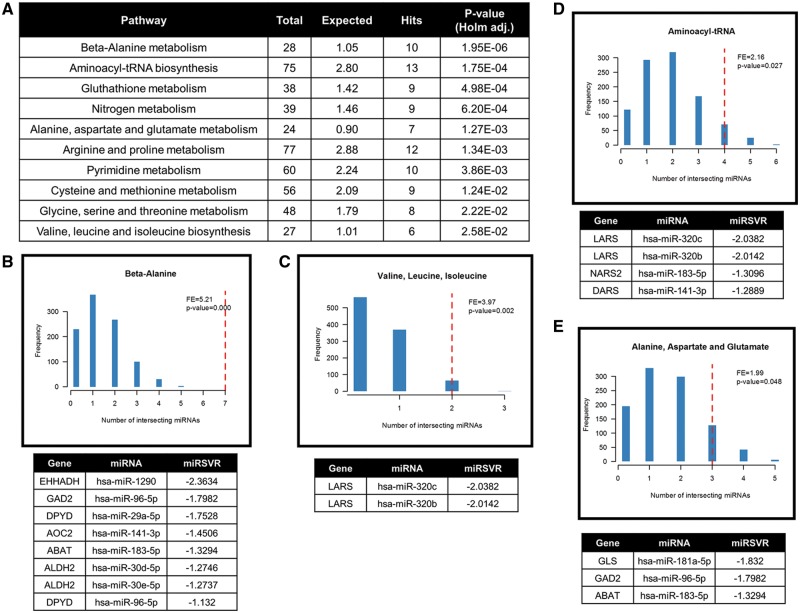
Cellular metabolism altered under hypoxic conditions in CRC cell lines*.* Metabolite analysis *via* CE-TOF/MS between six CRC cell lines maintained under normoxic (20.9%) and low oxygen conditions (1 and 0.2%). Over-representation analysis of significantly altered metabolites was performed using MetaboAnalyst. (**A**) Ten significant pathways associated with altered metabolites under hypoxic conditions. (**B**–**E**) *In silico* analysis of miRNA target prediction of genes involved in the ten pathways. Four pathways showed a significant association between hypoxia-responsive miRNAs. Genes with a miRSVR score lower than -1.25 are listed.

The microenvironment in tumours, in particular hypoxia, is a key driver of changes in metabolic pathways that impact on cell survival and drug-resistance, and contribute to recurrence and metastasis. Using capillary electrophoresis-time of flight mass spectrometry (CE-TOF/MS) we have identified changes in the metabolite expression profiles across the panel of six CRC cell lines under hypoxic conditions (1 and 0.2%) as compared with normoxia. A total of 92 significantly altered metabolites (*P* < 0.05) were identified after linear modelling following fitting for cell line and oxygen tension (see Supplementary Material, Table S4). We observed a significant increase in lactate in cells confirming the hypoxic state of the cells (see Supplementary Material, Fig. S2). To allow broad pathway analysis, significantly altered metabolites were subjected to an over-representation analysis (MetaboAnalyst, www.metaboanalyst.com). This identified ten metabolic pathways that are enriched when cells are cultured under hypoxia (Fig. 3A). To investigate the potential role of hypoxia-responsive miRNAs from our previous analysis (Fig. 1C) in modulating these pathways, *in silico* target analysis was performed on all miRNAs detected by the array. Interestingly, hypoxia-responsive miRNAs are significantly predicted to target genes in four pathways: beta-alanine (*P* < 0.0001, Fig. 3B); valine, leucine, iso-leucine metabolism (*P* = 0.002, Fig. 3C); aminoacyl-tRNA metabolism (*P* = 0.027, Fig. 3D); and alanine, aspartate, glutamate metabolism (*P* = 0.048, Fig. 3E). Genes targeted for each pathway by hypoxia-responsive miRNAs are listed and ranked by their corresponding microRNA support vector regression (miRSVR) score. These data indicate that hypoxia modulates amino acid metabolism and that this modulation could involve hypoxia-responsive miRNAs.

### MiR-210 is upregulated in CRC tissue and correlates positively with the hypoxia marker CAIX

**Figure 4:**
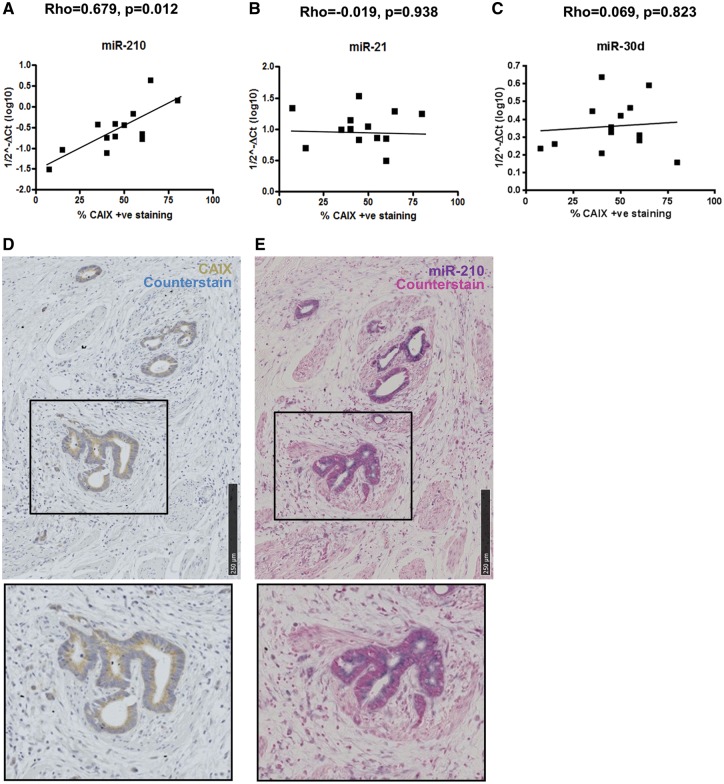
*In situ* expression of miR-210 positively correlates to hypoxia marker CAIX in CRC tissue. (**A**–**C**) Scatter plots of miR-210, miR-21 and miR-30d expression (1/2^−ΔCT^) assessed by qRT-PCR and percentage of CAIX-positive staining within the tumour (n = 13). Correlation efficiency calculated with Spearman’s Rank analysis showed a significant positive correlation between miR-210 and CAIX expression CRC tumours (**D** and **E**) IHC of CAIX and *in situ* hybridization of miR-210 performed on serial sections of FFPE blocks of CRC tumours (n = 8). Staining of miR-210 was observed in the epithelial cells along the crypts and overlapped with CAIX staining within these cells.

To corroborate our *in vitro* hypoxia-responsive data, the expression of candidate miRNAs was tested in 13 human CRC samples. Normal and tumour regions were identified on H&E stained paraffin sections of CRC tumour tissue and then small RNAs (including miRNAs) were extracted from appropriate regions of corresponding serial sections. The hypoxia marker CAIX was used as a comparator by staining a serial section with CAIX and the extent of expression within the CRC tumour determined according to Korkeila *et al.* (44). The expression of miR-210, miR-21 and miR-30d was assayed by qRT-PCR and correlation between the miRNA expression and CAIX staining within the tumour was determined. Spearman’s rank correlation demonstrated that the expression of miR-210 correlated positively to the expression of the hypoxia marker CAIX (*ρ* = 0.67, *P* = 0.01, Fig. 4A). In contrast, neither miR-21 nor miR-30d expression had any correlation to CAIX expression (miR-21, *ρ* = −0.019, *P* = 0.95; miR-30d, *ρ* = 0.069, *P* = 0.823; Fig. 4B and C).

To determine the localization of miR-210 within the tumour tissue, *in situ* hybridization was performed for miR-210 on sections available from 8 of the 13 FFPE blocks and compared with any corresponding hypoxic regions in a serial section stained for CAIX. In all tumours, both CAIX and miR-210 were expressed in the epithelial cells of the gut crypts and their expression overlapped in the majority of the tumours (Fig. 4D and E). Our data provided *in vivo* confirmation of the correlation between miR-210 and hypoxia identified in the CRC cell line panel.

### Differential miRNA expression in 5-FU resistant CRC tumours

**Figure 5:**
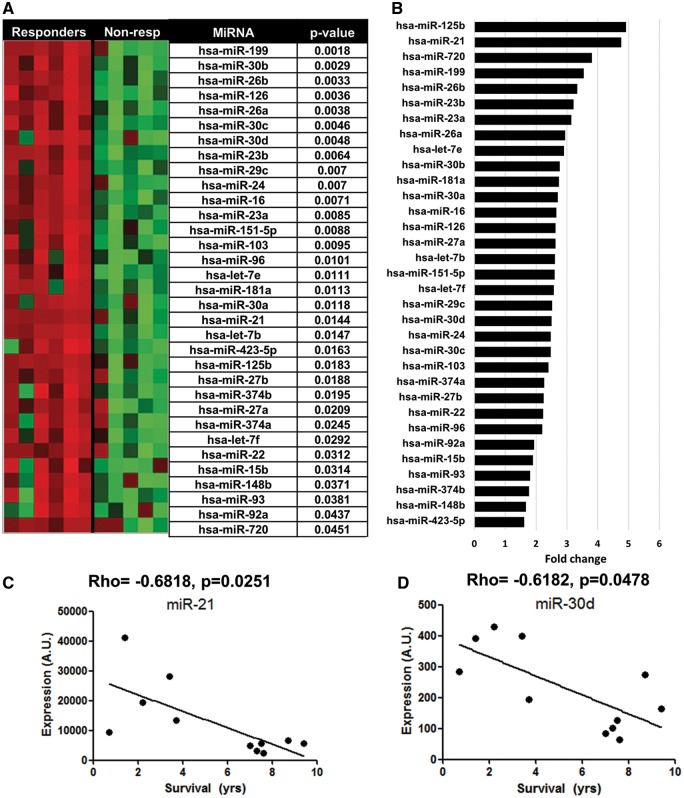
MiRNA expression profiling in 5FU resistant CRC tumours and modulation of resistance in hypoxic DLD-1 cells. Eleven CRC tumours treated with 5-FU treatment (six non-responders, five responders) were subjected to miRNA profiling. (**A**) Heatmap of significant altered miRNAs (*P* < 0.05, Benjamini-Hochberg adjusted). Corrected *P*-values are shown. (**B**) Fold change in expression of miRNAs significantly altered between each group. (**C** and **D**) Correlation between patient survival and expression of miR-21 or miR-30d. Spearman’s rank analysis indicated a significant correlation between each miRNA and patient survival.

5-FU is the standard first line treatment for CRC. However, resistance, partly as a consequence of tumour hypoxia, remains a major challenge (27–29). Therefore, we investigated which miRNAs are altered in CRC tumours of patients that had undergone adjuvant 5-FU treatment (*n* = 11) and for whom survival data were available (responders, >5 years survival post-surgery; non-responders, <5 years). Clinicopathological features of patients are given in Supplementary Material, Table S5. A total of 33 (4.1%, 33/800) significantly enriched miRNAs were identified by array profiling (see Materials and Methods) in tumours associated with non-response to 5-FU treatment (Fig. 5A, adjusted *P*-value <0.05). miR-21 and four members of the miR-30 family (miR-30a, b, c and d) showed the highest fold changes between the responders versus non-responders (Fig. 5B). Importantly, miR-210 was not amongst the significant miRNAs. A Spearman’s rank correlation analysis identified a significant negative correlation between the expression of both miR-21 (*ρ* = −0.6818; *P* = 0.02, Fig. 5C) and miR-30d (*ρ* = −0.6182, *P* = 0.0478, Fig. 5D) to patient survival.

### MiR-21 and miR-30d modulate 5-FU resistance in hypoxic DLD-1 cells

**Figure 6:**
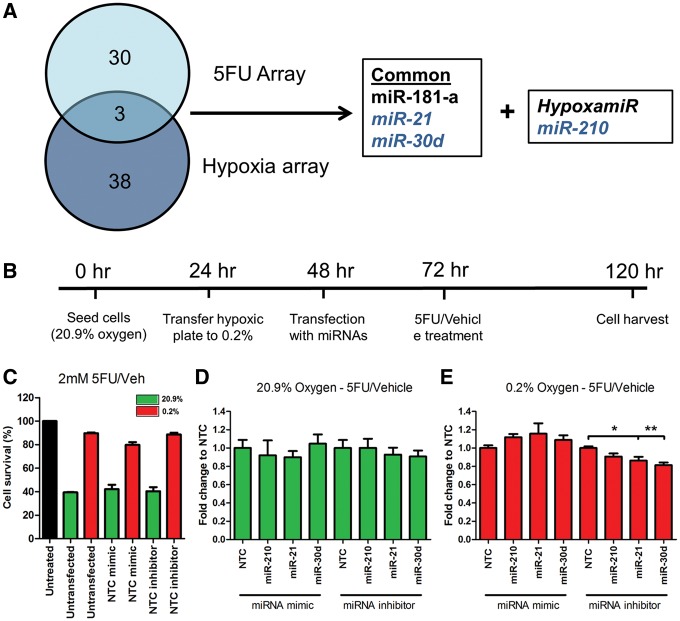
MiR-30d modulates 5-FU resistance in hypoxic DLD-1 cells. (**A**) Venn diagram of common miRNAs that are dysregulated in both arrays (*in vivo* hypoxia array and *ex vivo* CRC tissue array). (**B**) DLD-1 cells were transfected with miRNA mimic and inhibitors under normoxic and hypoxic conditions. Experimental timeline of the experiments is shown. (**C**) Cell survival following treatment with 2 mM 5-FU and vehicle (DMSO). Data normalized to untreated cells. (**D** and **E**) Graph represents fold change in survival following treatment with 5-FU and vehicle. Data normalized to vehicle and then to its transfection control (NTC) inhibition of miR-30d under hypoxic conditions resulted in a significant reduction in resistance to 5-FU. Bars represent mean values with SEM calculated from three individual experiments. *P < 0.05, **P < 0.01.

Our data suggest a possible role for altered miRNA profiles in 5-FU resistant CRC tumours. Interestingly, miR-21 and miR-30d are two of only three miRNAs that are common across both the *in vitro* hypoxia and *ex vivo* CRC tissue arrays (Fig. 6A).

The functional roles of miR-21 and miR-30d in 5-FU resistance under conditions of severe hypoxia (0.2%) were investigated *in vitro* in DLD-1 cells and contrasted with the hypoxamir miR-210. The rationale for selecting the DLD-1 cell lines for these experiments included the marked induction of the hypoxamir miR-210 in addition to miR-21 and 30d, which together had the highest fold change in hypoxic DLD-1 cells compared with the other cell lines (Fig. 2C). DLD-1 cells were grown under two oxygen conditions (20.9 or 0.2%) and transfected with miRNA mimic/inhibitor or their appropriate control prior to 5-FU treatment (Fig. 6B). Cells were treated with various concentrations of 5-FU and from these experiments a dose (2 mM) was selected that gives between 60 and 80% cell survival in 0.2% hypoxia (see Supplementary Materials, Fig. S3). Under normoxic conditions, treatment with this dose resulted in an average cell survival of 42 and 40% for the non-targeting control (NTC) mimic or inhibitor, respectively (green bars, Fig. 6C). Under 0.2% oxygen, cell survival increased to 80 and 89% following transfection with NTC mimic or inhibitor, respectively (red bars, Fig. 6C). Next, cells were transfected with the mimic or inhibitor of the three hypoxia-responsive miRNAs (miR-210, -21 and -30d) under normoxic and hypoxic conditions. Survival data for each miRNA were normalized to its appropriate NTC control. Under normoxic conditions, no change in cell survival was observed when cells were transfected with either the mimic or inhibitor of either miRNA (Fig. 6D). However, when cells were transfected with inhibitors for miR-21 or miR-30d under 0.2% oxygen a significant reduction in cell survival was observed compared with their NTC inhibitor control (miR-21 inhibitor, *P* = 0.0390; miR-30d inhibitor, *P* = 0.0047; Fig. 6E). Taken together, these data demonstrated that down-regulation of miR-21 or miR-30d can sensitise DLD-1 cancer cells to 5-FU under hypoxic conditions. This indicates a functional role for miR-21 and miR-30d in the molecular events leading to 5-FU resistance in low oxygen conditions.

## Discussion

We have identified altered miRNA and metabolism profiles in a panel of CRC lines (three colon and three rectum) at two hypoxic oxygen tensions. Our data reveal that while there are differences between the cell lines, a set of miRNAs changes in response to hypoxia can be identified, once allowance has been made for the effects of the cell line. Significant differences were highlighted in the expression of miRNAs between the three oxygen conditions and six miRNAs (miR-21, -210, -30d, -320a, -320b and -320c) were up-regulated consistently under both hypoxic conditions (1 and 0.2%) *in vitro*. We sought to confirm our data in tumour samples from patients to determine the link between 5-FU resistance, hypoxia and miRNAs. The hypoxamiR-210 has a key role in cellular responses to low oxygen conditions in cancer cell lines and has been linked to clinico-pathological features in CRC including outcome (26,38). Our data confirmed the importance of miR-210 in hypoxic CRC cells *in vitro*, which we now link to hypoxia *in vivo* by demonstrating co-expression with CAIX, a known marker of hypoxia in CRC, as a comparator (12,44). Both CAIX and miR-210 were expressed in the epithelial cells of the gut crypts and their expression overlapped. This highlights the potential use of miR-210 as an *in situ* hypoxia biomarker for use in CRC FFPE tissue, which has been suggested by others linking expression to prognostic factors including metastasis in CRC (26); but this requires large scale trials for validation. Given the importance of hypoxia in chemotherapy resistance, a larger scale investigation is now warranted to determine whether increased *in vivo* miR-210 expression *per se* can be used to predict patient outcome.

Resistance to 5-FU-based therapy poses a major challenge to clinical management. Therefore, we aimed to identify those miRNAs that are differentially expressed in CRC tumours that are either responsive or resistant to 5-FU-based therapy based on 5-year survival data. A change in the expression of a number of miRNAs in 5-FU resistant tumours relative to susceptible tumours was identified including the hypoxia-responsive miR-21 and miR-30d. Although numbers in our series was limited, we do corroborate the findings of others who reported that miR-21 was amongst the most strongly up-regulated miRNAs in 5-FU resistant CRC tumours and correlated significantly with reduced survival in patients with CRC and pathological response to 5-FU-based therapies in rectal cancer (45-47). In addition to confirming results for miR-21, we have now shown for the first time, that miR-30d is also up-regulated in 5-FU resistance CRC tumours and that this maybe associated with poor survival. The small sample set currently limits clinical utility and so our observational data requires further validation in a larger cohort where account is taken of the degree of hypoxia within the tumour. Our finding that DLD-1 cells can be sensitized to 5-FU with anti-miR-30d under hypoxic conditions provides further impetus.

Earlier studies showed that miR-21 over-expression reduced responsiveness to 5-FU-based therapies by targeting mutS homolog (*MSH*)*2*, *MSH6* and sprout homolog (*Spry*)*2* (47,48), and enhanced chemo-sensitivity in the HT-29 cell line to 5-FU (49). Both miR-30d and miR-21 have been reported to be up-regulated in the 5-FU resistant CRC cell line KM12C (39). However, the role of hypoxia was not explored in these studies. We have shown that the CRC cell line DLD-1 can be sensitized to 5-FU with anti-miR-21 as well as anti-miR-30d under hypoxic conditions. Interestingly, miR-21 expression is increased in CRC cell lines exposed to 5-FU (50). A hypoxia-driven increase in miR-21/miR-30d expression could be a potential mechanism for acquired resistance to chemotherapy drugs such as 5-FU.

Tumour cells are known to have a reprogrammed metabolism compared with normal cells, including specific features such as reliance on aerobic glycolysis (the Warburg effect) (51–53). We observed a large number of metabolic changes in the same panel of six CRC cell lines under hypoxia, beyond the expected alterations in key metabolites such as lactate. Metabolic pathway overrepresentation analysis should arguably be considered an exploratory technique, rather than a means of determining exactly which pathways have been altered by a treatment. Nonetheless, we observed changes in a number of pathways involved in amino acid metabolism. In particular, the inclusion of the high-level ‘pathway’ of aminoacyl-tRNA metabolism supports a hypothesis that overall changes in amino acid pools are probably driven by changes in protein synthesis rates controlled, in part, by hypoxia responsive miRNAs. These, in turn, may influence cell growth rates and response to drug treatment. In support of this, Frezza *et al.* also reported altered amino acid profiles under hypoxia in a single CRC cell line HCT116 (54). Changes in amino acid concentrations have been reported previously in CRC tumour tissue compared with normal gut mucosa, suggesting a link between modulated amino acid metabolism and CRC (55). Hypoxia can increase the expression of amino acid transporters such as solute carrier family 7 members 5 and 11 *via* HIFs (56,57), to cope with the rise of non-aerobic energy demands. The latter was shown to occur as a consequence of chemotherapy as well as hypoxia (57).

While miRNAs play complex roles in gene regulation using *in silico* analysis revealed a potential link between the hypoxia-responsive miRNAs and genes involved in amino acid metabolism. For example, dihydropropyrimidine dehydrogenase (DPYD), an enzyme active in amino acid metabolic pathways, including beta-alanine metabolism, is targeted by hypoxia-responsive miRNAs miR-29a and miR-96. Interestingly, variants in DPYD are predictive of toxicity by 5-FU-based therapies in cancer (58). Also, miR-494 sensitises CRC cell line SW480 to 5-FU by targeting *DPYD* (59), suggesting a link between amino acid metabolism and drug resistance in CRC. Our investigation on a possible link between metabolism and hypoxia-responsive miRNAs has highlighted more complexity than has hitherto been considered. Further experimental investigation of the exact role of miRNAs and the link between them and altered metabolism in hypoxia is required. Furthermore, this will require confirmation in oxygen-deprived regions of tumour tissue.

In summary, our work has demonstrated the complexities and multiple cellular consequences of hypoxia. It highlights the importance of interrogating a panel of cell lines and the challenge of integrating data across multiple platforms and between model systems including cell lines and tumour samples, with their inherent heterogeneity. As yet there is no definitive answer as to whether all or only a few hypoxia responsive miRNAs have a role of biological significance in the oxygen-deprived microenvironment of the tumour. Many of these roles still need to be determined, but it seems likely that both scenarios are true. Nevertheless, our *in vivo* analyses confirmed miR-210 as a CRC hypoxamir, indicating its potential as a biomarker of hypoxia, and determined the localized expression of miR-21 in CRC, which suggested an important role for this miRNA in tumour cell maintenance and survival. Furthermore, we have identified important roles for miR-21 and miR-30d in 5-FU resistance in hypoxia. We have also highlighted the metabolic remodelling that occurs with increasing hypoxia. Further work is now necessary to investigate whether targeting miR-21 and miR-30d and/or the amino acid metabolism pathways in the hypoxic regions of tumours represent an appropriate therapeutic approach.

## Materials and Methods

### Ethics statement

All methods were carried out in accordance with relevant guidelines and regulations. The Blizard Institute and local Ethics Committee (NRES Committee London; REC ref: 13/LO/1271) approved experimental protocols that involved FFPE tissue samples from patients. Informed consent was obtained.

### CRC cell lines and culture

Three colon cancer (DLD-1, HCT116, HT29) and three rectal cancer (HT55, SW837, VACO4S) cell lines (37) were maintained in 5% CO_2_ humidified atmosphere containing Dulbecco’s Modified Eagles Medium (DMEM, Invitrogen, UK) supplemented with 10% fetal bovine serum (FBS, Labtech, UK) and 10 000 U/ml penicillin and 10 000 µg/ml streptomycin (PenStrep, Sigma, UK). All cells were a kind gift from Professor Ian Tomlinson (Wellcome Institute of Human Genetics, Oxford, UK). Cells are regularly checked for known mutation status. For hypoxic experiments, cells were maintained at three different oxygen tensions (20.9, 1 and 0.2%) controlled by an Invivo2 1000 Hypoxia Workstation (Ruskinn Life Sciences Ltd) for 48 h.

### RNA extraction from cells and miRNA array

Cell lines were incubated under three oxygen conditions for 48 h. Extractions were performed with the miRNeasy kit (Qiagen, UK). RNA (2 μg) was subjected to the LNA seventh generation Array (1896 mature human miRNAs; total, 3100 miRNA probes, miRBase v19, Exiqon, Denmark). Raw data were background corrected and normalized to mean plate intensity.

### RNA extraction from FFPE tissue and miRNA array

All patients (n = 11) had received 5-FU-chemotherapy and based on years survival post-surgery were grouped into responders (survival >5 years) and non-responders (<5 years). A pathologist determined the tumour tissue area. Ten adjacent FFPE sections (10 µm) from each CRC were used for miRNA extractions with the RecoverAll Total Nucleic Acid Isolation kit (Ambion, Applied Biosystems, USA). MiRNA profiling was performed using the nCounter miRNA array (800 miRNAs, miRBase v12, Nanotechnologies, USA) because of its compatibility with FFPE tissue. Positive and negative control corrections were applied to the raw data, background was corrected and data were normalized using mean plate intensity.

### *In situ* hybridization of miRNAs

For *in situ* hybridization of miRNAs, 13 CRC tumours were used. Sections (5 µm) were cut in RNAse free water, deparaffinized, incubated with Proteinase-K (10 µg/ml) for 15 min at 37 °C, dehydrated and air-dried. Double-DIG-labelled miRNA probes (Exiqon, Denmark): miR-210 (40 nM) and scrambled (60 nM). Incubation was at 56 °C for 2 h with 25 µL of probe followed by washing with saline–sodium citrate. Slides were incubated with blocking buffer for 15 min, then with anti-DIG-Alkaline phophatase reagent (1:600, Exiqon, Denmark) at RT. Slides were washed with PBS–Tween and incubated with anti-AP substrate (NCT-BCIP, Roche, UK) for 2 h at RT, washed in water before counterstaining with Nuclear Fast Red (Vector, UK). Slides were rinsed in running tap water for 10 min, dehydrated, mounted with Eukkitt mounting medium (Sigma, UK) and then scanned using a Hamamatsu slide scanner.

### Quantitative real-time PCR

Ten nanograms RNA were used for reverse transcription with the microRNA Reverse Transcriptase kit (Applied Biosystems, UK). RT products were incubated with Taqman microRNA probe and Universal Mastermix (Applied Biosystems, UK) on a 7500 System RealTime PCR cycler (ABI) according to the manufacturer’s protocol. Fold changes were calculated using the 2^−^^ΔΔCT^ method normalized to the appropriate control. For CRC tissue, the geomean of miR-16 and let-7a was used as the endogenous control. For CRC cell lines RNU19b was used.

### Immunohistochemistry

Sections (4 µm) were de-waxed, hydrated and blocked in goat serum. Primary antibody for anti-CAIX (1:50, SantaCruz, UK) was used. Secondary antibody bound to HRP was incubated followed by washing, streptavidin-biotin and DAB incubation, and then counterstained with haematoxylin. Stomach tissue was used as positive control samples (44). Two observers, blinded to section details, assessed staining.

### MiRNA transfection and 5-FU treatments

DLD-1 cells were seeded in 96-well plates overnight then placed in either 20.9% or 0.2% oxygen. The next day, cells were transfected with 60 nM miRNA mimic or 120 nM miRNA inhibitor and their appropriate NTC (all from Qiagen, UK). Cells were treated with 5-FU (2 mM) the following day, for 48 h, before harvesting. Cells were fixed with 3.7% PFA for 15 min and nuclei were stained with Hoechst 33342. Cells were imaged with the IN Cell 1000 microscope (GE Healthcare, UK). Nuclei were counted using the IN Cell Developer Software v1.8.

### Metabolite analysis: CRC cell lines

The levels of metabolites (fmol/cell) were determined by CE-TOF/MS analysis. The six CRC cell lines were all cultured under the three oxygen conditions for 48 h as described above. Cells were washed two times in 5% Mannitol (Wako, Japan) and harvested in methanol containing methionine sulfone, 2-morpholinoethanesulfonate and d-camphor-10-sulfonic acid, each at 25 µM. Samples were flash frozen on dry ice and shipped to the Institute for Advanced Biosciences in Japan for CE-TOF/MS as described previously (60,61).

### Statistical analysis

General statistics were performed using Prism (Graphpad) analysis software. Student’s *t*-tests and one-way ANOVA tests were used. A *P*-value of <0.05 was considered statistically significant. Experiments were performed in duplicate in three independent experiments, unless otherwise stated.

For multivariate analysis, the supervised technique PLS-DA was used as this is particularly useful with highly multivariate datasets, but care must still be taken to avoid overfitting. One approach that can be very useful for experiments with multiple factors is to model only one factor as supervised, which may also simplify the interpretation of the remaining, unsupervised factor (62). To accomplish this, fitting with respect to one variable was performed, and then the separation examined in another variable. Here, a supervised model to differentiate the six cell lines was fitted, and the miRNAs were examined for (unsupervised) separation with respect to oxygen tension. The loadings were then interrogated in order to identify potential miRNAs for further investigations.

The multivariate analysis was complemented with univariate analyses. The relationship between miRNA expression (dependent variable) was modelled against the independent variables oxygen tension (numerical variable) + cell line (categorical variable) using multiple linear regression. This permitted the identification of miRNAs and metabolites that were significantly (*P* < 0.05) associated with oxygen tension after allowance was made for the effect of cell line.

The August 2010 release of predictive targets for each miRNA was obtained from http://www.microrna.org/microrna/getDownloads.do; date last accessed January 2017 for good mirSVR scores of both conserved and non-conserved miRNA (63). Gene pathway information was obtained from www.miRWalk.com and the number of genes targeted with a mirSVR score <−1.25 by the significant miRNAs was calculated. To calculate the fold enrichment of each of these pathways with our significant miRNAs a permutation analysis was performed. We randomly sampled 1000 different sets of miRNAs from the non-significant list in our experiment and calculated the overlap for each pathway. These values were compared with our significant values and the fold enrichment and exact *P*-value were calculated.

## Supplementary Material

Supplementary Material is available at *HMG* online.

## Supplementary Material

Supplementary Data
