# Supplementary material for: Remodelling of microRNAs in colorectal cancer by hypoxia alters metabolism profiles and 5-fluorouracil resistance
Source: Hum Mol Genet. 2017 Feb 16;26(8):1552–64. doi: 10.1093/hmg/ddx059 (PMC5393147; doi:10.1093/hmg/ddx059)

**Supplementary Tables**

**Supplementary Table S1. MicroRNA profiling for colorectal cancer cell lines at 20.9% versus 1% oxygen.** LogFC, Log2(FoldChange); AvEx, average expression level calculated based on all samples included in the comparison; AvH3, Log2 (average Hy3 hybridized fluorescent tag signal intensity; p-value calculated from t-statistics comparing the two sample groups; adj.P.Val, adjusted p-value from t-statistics comparing the two sample groups.

|  |  |  |  |  |  |  | **1% Oxygen** | | | | | | **normoxia** | | | | | |  |
| --- | --- | --- | --- | --- | --- | --- | --- | --- | --- | --- | --- | --- | --- | --- | --- | --- | --- | --- | --- |
| **ProbeID** | **Annotation** | **logFC** | **AvEx** | **AvH3** | **P.Value** | **adj.P.Val** | **HCT116** | **HT29** | **SW837** | **VACO** | **DLD1** | **HT55** | **SW837** | **HCT116** | **HT29** | **DLD1** | **HT55** | **VACO** | |
| 42485 | ebv-miR-BART10-5p | 0.380 | 0.773 | 5.778 | 3.41E-03 | 4.83E-02 | 1.108 | 1.475 | 1.203 | 0.818 | 0.606 | 0.568 | 0.469 | 0.748 | 0.828 | 0.391 | 0.546 | 0.514 | |
| 17306 | ebv-miR-BART12 | -0.068 | 0.903 | 5.755 | 7.14E-01 | 8.39E-01 | 0.784 | 0.734 | 0.500 | 1.106 | 1.016 | 1.074 | 1.552 | 1.026 | 0.870 | 0.894 | 0.718 | 0.562 | |
| 42897 | ebv-miR-BART15 | 0.070 | 0.674 | 5.836 | 3.43E-01 | 5.54E-01 | 0.544 | 0.605 | 0.852 | 0.648 | 0.677 | 0.932 | 0.606 | 0.702 | 0.633 | 0.648 | 0.691 | 0.556 | |
| 42522 | ebv-miR-BART19-3p | 0.015 | 0.862 | 6.858 | 8.55E-01 | 9.14E-01 | 0.763 | 0.755 | 0.664 | 1.028 | 1.111 | 0.897 | 0.536 | 0.913 | 0.903 | 0.744 | 1.008 | 1.022 | |
| 145990 | ebv-miR-BART21-3p | 0.111 | 0.871 | 5.741 | 5.41E-01 | 7.29E-01 | 0.692 | 0.709 | 0.757 | 1.350 | 1.132 | 0.919 | 1.468 | 1.008 | 0.479 | 0.772 | 0.659 | 0.506 | |
| 42610 | hcmv-miR-UL36-3p | 0.544 | 0.605 | 5.591 | 1.34E-03 | 4.35E-02 | 1.109 | 1.491 | 1.106 | 0.700 | 0.550 | 0.305 | 0.288 | 0.544 | 0.600 | 0.093 | 0.397 | 0.071 | |
| 17619 | hcmv-miR-US25-2-5p | 0.033 | 0.847 | 5.757 | 8.66E-01 | 9.20E-01 | 0.674 | 0.703 | 0.490 | 1.197 | 1.301 | 0.819 | 1.148 | 1.226 | 0.864 | 0.730 | 0.646 | 0.372 | |
| 147162 | hsa-let-7a-5p | 0.031 | 0.031 | 7.676 | 6.27E-01 | 7.80E-01 | -0.119 | 0.235 | 0.011 | -0.180 | 0.209 | 0.123 | 0.113 | -0.235 | 0.230 | 0.183 | -0.087 | -0.112 | |
| 147165 | hsa-let-7b-5p | 0.066 | -0.530 | 8.696 | 4.42E-01 | 6.42E-01 | -1.431 | -0.071 | -0.526 | -0.466 | 0.033 | -0.519 | -0.633 | -1.686 | -0.316 | 0.346 | -0.610 | -0.475 | |
| 145820 | hsa-let-7c | 0.130 | -0.015 | 6.654 | 1.06E-01 | 2.60E-01 | -0.483 | 0.352 | -0.144 | -0.069 | 0.705 | -0.063 | -0.196 | -0.571 | -0.081 | 0.510 | -0.120 | -0.023 | |
| 145968 | hsa-let-7d-5p | 0.011 | 0.120 | 7.260 | 8.71E-01 | 9.23E-01 | 0.139 | 0.425 | 0.057 | -0.110 | 0.231 | 0.010 | 0.122 | 0.033 | 0.360 | 0.044 | 0.021 | 0.105 | |
| 145846 | hsa-let-7e-5p | 0.095 | -0.259 | 6.801 | 2.35E-01 | 4.31E-01 | 0.402 | 0.977 | 0.141 | -1.373 | -1.355 | -0.063 | 0.082 | 0.220 | 0.747 | -1.239 | -0.383 | -1.270 | |
| 17752 | hsa-let-7f-5p | -0.027 | 0.472 | 6.109 | 6.95E-01 | 8.29E-01 | 0.256 | 0.510 | 0.314 | 0.446 | 0.652 | 0.571 | 0.529 | 0.329 | 0.322 | 0.588 | 0.599 | 0.543 | |
| 46438 | hsa-let-7g-5p | 0.021 | 0.145 | 7.871 | 7.94E-01 | 8.75E-01 | 0.503 | 0.084 | -0.056 | -0.278 | 0.200 | 0.478 | -0.162 | 0.355 | -0.078 | 0.258 | 0.389 | 0.046 | |
| 9938 | hsa-let-7i-5p | 0.024 | 0.194 | 7.452 | 7.09E-01 | 8.38E-01 | 0.419 | 0.891 | 0.144 | -0.282 | 0.109 | -0.044 | 0.125 | 0.338 | 0.680 | 0.127 | 0.005 | -0.180 | |
| 31026 | hsa-miR-101-3p | 0.391 | 0.052 | 6.389 | 2.57E-03 | 4.35E-02 | 0.301 | 1.009 | 0.308 | 0.028 | -0.117 | -0.043 | 0.127 | 0.274 | 0.330 | -0.643 | -0.670 | -0.280 | |
| 10919 | hsa-miR-103a-3p | 0.097 | 0.066 | 7.925 | 1.71E-01 | 3.55E-01 | 0.144 | 0.623 | -0.002 | 0.078 | -0.135 | -0.022 | -0.173 | -0.134 | 0.429 | -0.083 | -0.025 | 0.088 | |
| 46801 | hsa-miR-106a-5p | 0.059 | 0.037 | 7.885 | 3.97E-01 | 5.87E-01 | -0.048 | 0.275 | -0.495 | 0.440 | -0.167 | 0.392 | -0.549 | -0.195 | 0.323 | -0.225 | 0.128 | 0.562 | |
| 17854 | hsa-miR-106b-3p | 0.084 | 0.401 | 6.425 | 4.34E-01 | 6.33E-01 | 0.583 | 0.767 | 0.591 | 0.064 | 0.354 | 0.300 | 0.088 | 0.459 | 0.812 | 0.021 | 0.517 | 0.259 | |
| 19582 | hsa-miR-106b-5p | 0.242 | 0.029 | 8.395 | 2.52E-03 | 4.35E-02 | 0.135 | 0.887 | 0.243 | -0.463 | -0.222 | 0.319 | 0.026 | -0.108 | 0.542 | -0.364 | -0.039 | -0.611 | |
| 10923 | hsa-miR-107 | 0.031 | 0.377 | 6.853 | 6.22E-01 | 7.80E-01 | 0.185 | 0.587 | 0.197 | 0.403 | 0.585 | 0.396 | 0.232 | 0.258 | 0.438 | 0.451 | 0.313 | 0.477 | |
| 28019 | hsa-miR-10a-3p | 0.312 | -0.217 | 5.464 | 3.69E-02 | 1.42E-01 | -0.647 | 0.142 | -0.374 | -0.524 | 1.242 | -0.208 | -0.997 | -1.081 | -0.009 | 0.476 | -0.399 | -0.229 | |
| 13485 | hsa-miR-10a-5p | -0.102 | -0.333 | 8.056 | 3.87E-01 | 5.83E-01 | -2.250 | 0.255 | -1.117 | -0.285 | 0.996 | 0.099 | -1.347 | -2.194 | 0.069 | 1.184 | 0.201 | 0.396 | |
| 42969 | hsa-miR-10b-3p | -0.053 | 0.602 | 5.828 | 5.12E-01 | 6.96E-01 | 0.414 | 0.422 | 0.482 | 0.596 | 0.854 | 0.689 | 0.686 | 0.643 | 0.548 | 0.576 | 0.643 | 0.676 | |
| 10925 | hsa-miR-10b-5p | 0.006 | 0.532 | 5.860 | 9.21E-01 | 9.48E-01 | 0.179 | 0.469 | 0.118 | 0.762 | 1.144 | 0.541 | 0.152 | 0.222 | 0.453 | 0.957 | 0.461 | 0.930 | |
| 42848 | hsa-miR-1180 | 0.391 | 0.663 | 5.700 | 6.87E-03 | 6.11E-02 | 1.095 | 1.384 | 1.093 | 0.787 | 0.349 | 0.447 | 0.488 | 0.508 | 0.612 | 0.209 | 0.544 | 0.443 | |
| 46345 | hsa-miR-1207-3p | -0.100 | 1.293 | 6.193 | 4.90E-01 | 6.79E-01 | 0.906 | 1.036 | 1.005 | 1.615 | 1.480 | 1.414 | 1.383 | 1.428 | 1.553 | 1.351 | 1.182 | 1.161 | |
| 46531 | hsa-miR-1231 | 0.150 | 0.632 | 5.670 | 8.70E-02 | 2.31E-01 | 0.645 | 0.545 | 0.708 | 0.570 | 0.949 | 0.822 | 0.645 | 0.726 | 0.392 | 0.443 | 0.695 | 0.437 | |
| 46624 | hsa-miR-1236-3p | 0.009 | 0.776 | 5.826 | 9.14E-01 | 9.47E-01 | 0.780 | 0.897 | 0.682 | 0.755 | 0.808 | 0.759 | 0.719 | 0.490 | 1.202 | 0.831 | 0.652 | 0.735 | |
| 46850 | hsa-miR-1237-3p | -0.114 | 0.828 | 5.921 | 3.74E-01 | 5.83E-01 | 0.610 | 0.566 | 0.707 | 0.893 | 0.932 | 0.918 | 1.154 | 1.232 | 0.759 | 0.775 | 0.666 | 0.726 | |
| 46690 | hsa-miR-1238-3p | 0.021 | 0.585 | 5.692 | 8.16E-01 | 8.88E-01 | 0.793 | 0.553 | 0.348 | 0.566 | 0.699 | 0.615 | 0.675 | 0.643 | 0.418 | 0.339 | 0.683 | 0.685 | |
| 42898 | hsa-miR-124-5p | 0.136 | 0.559 | 5.793 | 8.43E-02 | 2.29E-01 | 0.668 | 0.625 | 0.609 | 0.707 | 0.632 | 0.525 | 0.381 | 0.735 | 0.305 | 0.416 | 0.575 | 0.536 | |
| 168870 | hsa-miR-1246 | 0.215 | -0.920 | 11.392 | 2.01E-01 | 3.86E-01 | -1.043 | -1.456 | -0.921 | 0.806 | -2.075 | -0.188 | -1.143 | -1.652 | -1.640 | -1.369 | -0.665 | 0.302 | |
| 145977 | hsa-miR-1247-5p | 0.270 | -0.106 | 5.702 | 2.01E-02 | 1.01E-01 | -0.324 | -0.103 | -0.026 | 0.560 | -0.296 | 0.366 | -0.631 | -0.609 | -0.543 | -0.610 | 0.535 | 0.414 | |
| 46427 | hsa-miR-1248 | 0.305 | 0.593 | 5.847 | 2.55E-02 | 1.14E-01 | 0.913 | 1.010 | 1.193 | 0.585 | 0.466 | 0.306 | 0.308 | 0.579 | 0.670 | 0.178 | 0.419 | 0.488 | |
| 46210 | hsa-miR-1249 | 0.019 | 0.950 | 6.456 | 7.80E-01 | 8.73E-01 | 0.981 | 0.905 | 1.014 | 1.022 | 1.028 | 0.806 | 0.805 | 1.124 | 0.944 | 0.994 | 0.908 | 0.865 | |
| 10928 | hsa-miR-125a-5p | 0.040 | -0.237 | 6.661 | 6.57E-01 | 7.93E-01 | 0.337 | 1.129 | 0.214 | -1.616 | -1.499 | 0.136 | 0.221 | 0.232 | 0.807 | -1.265 | -0.131 | -1.405 | |
| 30787 | hsa-miR-125b-5p | -0.014 | -0.812 | 6.107 | 8.88E-01 | 9.30E-01 | 2.289 | -1.853 | -1.007 | -1.867 | -0.744 | -1.731 | -1.215 | 1.955 | -1.899 | -0.658 | -1.512 | -1.502 | |
| 4610 | hsa-miR-126-3p | -0.003 | 0.160 | 5.668 | 9.59E-01 | 9.71E-01 | 0.858 | -0.113 | 0.226 | 0.169 | -0.188 | 0.001 | 0.395 | 0.797 | -0.213 | -0.125 | -0.006 | 0.123 | |
| 169412 | hsa-miR-1260a | 0.233 | -0.814 | 8.225 | 4.61E-02 | 1.58E-01 | -0.298 | -0.837 | -0.005 | -1.306 | -0.889 | -0.847 | -0.550 | -0.859 | -0.840 | -0.938 | -1.176 | -1.218 | |
| 168619 | hsa-miR-1260b | 0.085 | -0.760 | 13.308 | 6.29E-01 | 7.80E-01 | -0.210 | -0.555 | 0.255 | -1.388 | -1.460 | -0.944 | 0.017 | -0.960 | -0.593 | -0.605 | -1.285 | -1.388 | |
| 46732 | hsa-miR-1264 | 0.037 | -0.400 | 8.101 | 5.57E-01 | 7.33E-01 | -0.460 | -0.303 | -0.022 | -0.732 | -0.185 | -0.586 | -0.034 | -0.412 | -0.401 | -0.287 | -0.765 | -0.612 | |
| 168925 | hsa-miR-1273g-3p | 0.072 | -0.835 | 7.422 | 3.45E-01 | 5.54E-01 | -0.976 | -1.268 | -0.300 | -0.722 | -0.686 | -0.838 | -0.323 | -0.875 | -1.135 | -0.944 | -1.081 | -0.865 | |
| 169082 | hsa-miR-1275 | 0.192 | -0.643 | 6.884 | 5.33E-02 | 1.69E-01 | -0.586 | -0.656 | -0.253 | -0.435 | -0.710 | -0.640 | -0.463 | -0.698 | -1.280 | -0.787 | -0.599 | -0.608 | |
| 46634 | hsa-miR-1281 | -0.136 | 0.525 | 5.865 | 2.26E-01 | 4.18E-01 | 0.275 | 0.323 | 0.301 | 0.715 | 0.608 | 0.518 | 0.648 | 0.752 | 0.699 | 0.473 | 0.496 | 0.488 | |
| 46368 | hsa-miR-1282 | 0.030 | 0.964 | 6.148 | 6.80E-01 | 8.14E-01 | 1.022 | 0.964 | 0.944 | 1.028 | 1.077 | 0.837 | 0.856 | 1.140 | 0.806 | 0.981 | 1.042 | 0.869 | |
| 46440 | hsa-miR-1287 | 0.039 | 0.674 | 5.790 | 6.09E-01 | 7.72E-01 | 0.746 | 0.697 | 0.614 | 0.729 | 0.825 | 0.551 | 0.683 | 0.750 | 0.757 | 0.446 | 0.619 | 0.672 | |
| 168568 | hsa-miR-1290 | 0.182 | -0.383 | 5.679 | 1.78E-02 | 9.90E-02 | -0.707 | -0.809 | -0.466 | 0.832 | -0.651 | 0.052 | -0.614 | -0.752 | -0.917 | -0.845 | -0.147 | 0.433 | |
| 46416 | hsa-miR-1293 | 0.177 | 0.779 | 5.775 | 2.09E-02 | 1.02E-01 | 0.844 | 0.872 | 0.916 | 0.712 | 0.967 | 0.893 | 0.711 | 0.784 | 0.700 | 0.568 | 0.735 | 0.643 | |
| 46215 | hsa-miR-1301 | 0.099 | 0.500 | 5.686 | 3.54E-01 | 5.65E-01 | 0.722 | 0.304 | 0.329 | 0.485 | 0.858 | 0.601 | 0.563 | 0.823 | -0.159 | 0.437 | 0.489 | 0.550 | |
| 46223 | hsa-miR-1306-3p | 0.026 | 0.796 | 5.714 | 9.05E-01 | 9.40E-01 | 0.423 | 0.525 | 0.437 | 1.389 | 1.065 | 1.013 | 0.864 | 1.439 | 0.669 | 0.658 | 0.481 | 0.585 | |
| 10936 | hsa-miR-130b-3p | 0.001 | 0.269 | 6.666 | 9.89E-01 | 9.92E-01 | 0.739 | -0.030 | 0.860 | -0.325 | 0.325 | 0.051 | 0.860 | 0.659 | 0.056 | 0.228 | -0.018 | -0.170 | |
| 42839 | hsa-miR-135a-5p | 0.062 | 0.785 | 6.052 | 3.44E-01 | 5.54E-01 | 0.663 | 0.903 | 0.946 | 0.609 | 0.732 | 1.042 | 1.002 | 0.661 | 0.766 | 0.580 | 0.840 | 0.674 | |
| 145914 | hsa-miR-135b-5p | 0.070 | -0.129 | 5.603 | 5.49E-01 | 7.31E-01 | -0.742 | 0.295 | 0.669 | -0.483 | -0.818 | 0.513 | 0.807 | -0.622 | 0.033 | -0.605 | -0.128 | -0.468 | |
| 10943 | hsa-miR-136-5p | 0.625 | 0.409 | 5.597 | 4.37E-03 | 5.22E-02 | 0.912 | 1.046 | 1.511 | 0.386 | 0.296 | 0.177 | 0.196 | 0.465 | -0.181 | -0.067 | 0.070 | 0.095 | |
| 148278 | hsa-miR-138-2-3p | 0.048 | -0.180 | 6.072 | 4.94E-01 | 6.80E-01 | -0.036 | -0.076 | -0.061 | -0.443 | -0.180 | -0.142 | -0.145 | -0.252 | -0.071 | -0.350 | -0.157 | -0.252 | |
| 10946 | hsa-miR-141-3p | 0.588 | -0.454 | 9.333 | 2.02E-03 | 4.35E-02 | -0.707 | 0.144 | -0.077 | 0.111 | -0.617 | 0.190 | -0.450 | -0.893 | -0.657 | -0.758 | -0.974 | -0.756 | |
| 10947 | hsa-miR-142-3p | 0.175 | 0.780 | 6.296 | 1.26E-01 | 2.81E-01 | 0.280 | 0.749 | 0.300 | 0.527 | 1.554 | 1.792 | 0.388 | 0.425 | 0.596 | 1.057 | 1.232 | 0.455 | |
| 42641 | hsa-miR-145-5p | 0.026 | 0.514 | 5.942 | 7.30E-01 | 8.46E-01 | 0.839 | 0.440 | 0.349 | 0.476 | 0.614 | 0.445 | 0.383 | 0.995 | 0.313 | 0.303 | 0.534 | 0.482 | |
| 146072 | hsa-miR-1469 | 0.180 | -0.380 | 5.617 | 9.37E-03 | 7.16E-02 | -0.649 | -0.498 | 0.675 | -0.062 | -0.736 | -0.471 | 0.480 | -0.765 | -0.704 | -0.879 | -0.600 | -0.355 | |
| 146052 | hsa-miR-1471 | 0.139 | 0.801 | 5.764 | 4.00E-01 | 5.89E-01 | 0.463 | 0.775 | 0.711 | 1.147 | 1.111 | 1.016 | 0.829 | 1.178 | 0.125 | 0.742 | 0.750 | 0.765 | |
| 10954 | hsa-miR-147a | 0.394 | 0.988 | 5.905 | 9.80E-04 | 4.35E-02 | 1.402 | 1.545 | 0.752 | 0.990 | 1.061 | 1.359 | 0.485 | 1.036 | 0.813 | 0.753 | 0.818 | 0.840 | |
| 19585 | hsa-miR-148b-3p | 0.055 | 0.582 | 6.278 | 3.85E-01 | 5.83E-01 | 0.697 | 0.732 | 0.566 | 0.661 | 0.576 | 0.428 | 0.635 | 0.742 | 0.503 | 0.480 | 0.380 | 0.588 | |
| 42810 | hsa-miR-149-5p | 0.283 | 0.414 | 5.588 | 4.39E-02 | 1.55E-01 | 0.885 | 0.783 | 1.112 | 0.271 | 0.202 | 0.083 | 0.208 | 0.580 | 0.397 | -0.013 | 0.217 | 0.246 | |
| 17463 | hsa-miR-151a-3p | 0.145 | 0.256 | 6.388 | 6.55E-02 | 1.91E-01 | 0.571 | 1.251 | 0.078 | -0.005 | 0.087 | -0.013 | -0.194 | 0.230 | 1.022 | 0.086 | -0.026 | -0.017 | |
| 11260 | hsa-miR-151a-5p | 0.121 | 0.192 | 6.470 | 1.04E-01 | 2.60E-01 | 0.477 | 1.407 | -0.016 | -0.198 | -0.206 | 0.047 | -0.142 | 0.313 | 1.050 | -0.253 | -0.047 | -0.133 | |
| 168871 | hsa-miR-151a-5p/-151b | 0.136 | -0.152 | 6.412 | 1.01E-01 | 2.57E-01 | 0.272 | 1.015 | -0.417 | -0.505 | -0.629 | -0.236 | -0.320 | 0.144 | 0.923 | -1.044 | -0.485 | -0.536 | |
| 10964 | hsa-miR-155-5p | -0.030 | 0.520 | 6.055 | 8.07E-01 | 8.84E-01 | -0.052 | 0.285 | 0.903 | 1.467 | 0.197 | 0.229 | 1.238 | 0.225 | 0.204 | 0.409 | 0.263 | 0.868 | |
| 27720 | hsa-miR-15a-5p | 0.052 | 0.360 | 7.413 | 4.52E-01 | 6.50E-01 | -0.853 | 0.537 | 0.126 | 1.034 | 0.678 | 0.796 | 0.001 | -0.635 | 0.419 | 0.502 | 0.722 | 0.995 | |
| 17280 | hsa-miR-15b-5p | -0.047 | 0.335 | 7.811 | 5.08E-01 | 6.96E-01 | -0.257 | 0.585 | 0.786 | 0.023 | 0.315 | 0.416 | 0.661 | -0.104 | 0.628 | 0.350 | 0.326 | 0.287 | |
| 10967 | hsa-miR-16-5p | 0.075 | -0.035 | 9.085 | 4.65E-01 | 6.58E-01 | -0.271 | 0.178 | 0.255 | -0.088 | -0.284 | 0.222 | 0.029 | -0.591 | 0.087 | -0.069 | -0.109 | 0.216 | |
| 169336 | hsa-miR-17-5p | 0.042 | -0.115 | 7.990 | 5.92E-01 | 7.63E-01 | -0.121 | 0.180 | -0.721 | 0.224 | -0.422 | 0.292 | -0.864 | -0.343 | 0.161 | -0.398 | 0.127 | 0.498 | |
| 42865 | hsa-miR-181a-5p | 0.150 | 0.046 | 6.162 | 1.10E-01 | 2.61E-01 | -0.644 | 0.910 | -0.622 | -0.551 | 0.413 | 1.223 | -0.853 | -0.776 | 0.609 | 0.203 | 0.915 | -0.270 | |
| 10972 | hsa-miR-181b-5p | 0.012 | -0.181 | 5.505 | 8.58E-01 | 9.15E-01 | -0.525 | 0.170 | -0.569 | -0.289 | -0.034 | 0.196 | -0.596 | -0.380 | 0.149 | -0.226 | 0.129 | -0.195 | |
| 169408 | hsa-miR-181d | 0.282 | -0.257 | 5.376 | 1.96E-02 | 1.01E-01 | -0.197 | 0.486 | -0.160 | -0.690 | -0.294 | 0.156 | -0.745 | -0.657 | -0.027 | -0.370 | -0.017 | -0.574 | |
| 10975 | hsa-miR-182-5p | 0.286 | -0.181 | 6.280 | 1.76E-02 | 9.90E-02 | 0.703 | -0.076 | -0.143 | -1.199 | 0.198 | 0.290 | -0.265 | 0.243 | -0.571 | -0.374 | 0.074 | -1.047 | |
| 46266 | hsa-miR-1825 | 0.196 | 0.446 | 5.769 | 1.81E-02 | 9.90E-02 | 0.608 | 0.621 | 0.652 | 0.407 | 0.510 | 0.463 | 0.230 | 0.386 | 0.440 | 0.247 | 0.379 | 0.407 | |
| 10977 | hsa-miR-183-5p | 0.209 | -0.120 | 6.228 | 1.80E-02 | 9.90E-02 | 0.505 | 0.109 | -0.035 | -0.784 | 0.194 | -0.084 | -0.289 | 0.188 | -0.031 | -0.264 | -0.163 | -0.786 | |
| 42902 | hsa-miR-185-5p | -0.030 | 0.184 | 6.539 | 6.49E-01 | 7.87E-01 | 0.151 | 0.912 | 0.379 | -0.283 | 0.140 | -0.286 | 0.341 | 0.251 | 0.976 | -0.049 | -0.206 | -0.119 | |
| 18739 | hsa-miR-186-5p | 0.166 | 0.087 | 5.863 | 2.29E-02 | 1.08E-01 | 0.545 | 0.581 | 0.095 | -0.088 | 0.073 | -0.189 | -0.173 | 0.506 | 0.436 | -0.250 | -0.324 | -0.174 | |
| 145670 | hsa-miR-18b-5p | 0.212 | 0.215 | 6.441 | 1.29E-02 | 8.59E-02 | 0.662 | 0.538 | -0.181 | 0.584 | -0.048 | 0.372 | -0.519 | 0.607 | 0.517 | -0.433 | 0.093 | 0.390 | |
| 27536 | hsa-miR-190a | 0.197 | 0.749 | 6.105 | 2.36E-02 | 1.08E-01 | 0.607 | 0.409 | 0.656 | 1.134 | 0.892 | 1.385 | 0.383 | 0.556 | 0.388 | 0.593 | 0.939 | 1.042 | |
| 42705 | hsa-miR-191-3p | 0.107 | 0.783 | 5.714 | 4.77E-01 | 6.69E-01 | 0.658 | 0.753 | 0.370 | 0.640 | 1.216 | 1.381 | 0.798 | 0.687 | 0.874 | 0.693 | 0.622 | 0.701 | |
| 10985 | hsa-miR-191-5p | -0.001 | -0.169 | 7.593 | 9.92E-01 | 9.92E-01 | 0.031 | -0.521 | -0.718 | 0.299 | 0.150 | -0.256 | -0.636 | 0.043 | -0.465 | 0.055 | -0.242 | 0.233 | |
| 146103 | hsa-miR-1913 | 0.017 | 0.693 | 6.146 | 7.91E-01 | 8.75E-01 | 0.603 | 0.650 | 0.660 | 0.720 | 0.870 | 0.704 | 0.704 | 0.741 | 0.686 | 0.656 | 0.627 | 0.692 | |
| 17946 | hsa-miR-192-3p | 0.406 | 0.195 | 5.680 | 8.08E-03 | 6.32E-02 | 0.232 | 0.603 | 0.518 | 0.663 | -0.186 | 0.557 | -0.477 | -0.152 | 0.120 | -0.083 | 0.274 | 0.270 | |
| 17732 | hsa-miR-192-5p | 0.246 | -1.274 | 7.357 | 3.38E-02 | 1.34E-01 | -3.825 | 0.530 | -3.253 | 1.063 | -2.418 | 0.996 | -3.683 | -3.690 | 0.123 | -2.343 | 0.517 | 0.695 | |
| 10986 | hsa-miR-193a-3p | 0.475 | -0.525 | 5.591 | 5.28E-03 | 5.47E-02 | -0.789 | -0.796 | -0.412 | 1.006 | -0.506 | -0.223 | -0.745 | -0.673 | -1.064 | -1.135 | -1.004 | 0.049 | |
| 46443 | hsa-miR-193a-5p | -0.085 | 0.502 | 5.722 | 4.51E-01 | 6.50E-01 | 0.216 | 0.284 | 0.233 | 0.750 | 0.764 | 0.510 | 0.629 | 0.740 | 0.389 | 0.568 | 0.386 | 0.556 | |
| 10987 | hsa-miR-193b-3p | 0.593 | -0.828 | 5.960 | 1.87E-02 | 9.90E-02 | -0.369 | -0.368 | 0.091 | -0.947 | -1.473 | -0.126 | 0.295 | -1.147 | -0.734 | -1.484 | -1.632 | -2.043 | |
| 10988 | hsa-miR-194-5p | 0.197 | -1.019 | 7.059 | 3.30E-02 | 1.34E-01 | -2.770 | 0.234 | -2.804 | 0.953 | -1.961 | 0.821 | -3.160 | -2.721 | -0.021 | -1.912 | 0.506 | 0.599 | |
| 10990 | hsa-miR-196a-5p | 0.117 | 0.441 | 6.790 | 1.86E-01 | 3.66E-01 | 0.075 | 0.878 | 0.287 | 0.220 | 0.709 | 0.828 | 0.136 | -0.237 | 0.529 | 0.580 | 0.880 | 0.411 | |
| 145889 | hsa-miR-196b-5p | 0.103 | 0.535 | 5.857 | 1.30E-01 | 2.87E-01 | 0.615 | 0.791 | 0.544 | 0.259 | 0.727 | 0.585 | 0.450 | 0.576 | 0.489 | 0.561 | 0.600 | 0.226 | |
| 146140 | hsa-miR-1976 | -0.052 | 0.753 | 5.765 | 7.33E-01 | 8.46E-01 | 0.540 | 0.648 | 0.484 | 0.831 | 1.001 | 0.856 | 0.808 | 1.298 | 0.861 | 0.661 | 0.521 | 0.525 | |
| 10997 | hsa-miR-19a-3p | 0.223 | 0.034 | 7.863 | 6.02E-03 | 5.69E-02 | 0.518 | 0.302 | -0.335 | 0.577 | -0.413 | 0.225 | -0.475 | 0.372 | 0.037 | -0.598 | -0.221 | 0.419 | |
| 10998 | hsa-miR-19b-3p | 0.195 | -0.099 | 7.276 | 1.26E-02 | 8.59E-02 | -0.487 | 0.179 | -0.672 | 0.657 | -0.072 | 0.385 | -0.768 | -0.502 | -0.017 | -0.404 | 0.089 | 0.419 | |
| 168819 | hsa-miR-200a-3p | 0.027 | -0.650 | 7.586 | 7.86E-01 | 8.75E-01 | -3.446 | 0.648 | -0.528 | -0.662 | -0.573 | 0.744 | -0.658 | -2.967 | 0.637 | -0.763 | 0.468 | -0.698 | |
| 147186 | hsa-miR-200b-3p | -0.078 | -0.477 | 8.582 | 2.75E-01 | 4.80E-01 | -2.943 | 0.762 | -0.502 | -0.618 | -0.542 | 0.747 | -0.589 | -3.007 | 0.810 | -0.318 | 0.832 | -0.353 | |
| 145974 | hsa-miR-200b-5p | 0.229 | 0.116 | 5.666 | 2.07E-02 | 1.02E-01 | -0.125 | 0.652 | 0.253 | -0.125 | 0.244 | 0.486 | -0.229 | -0.466 | 0.399 | -0.083 | 0.416 | -0.027 | |
| 17427 | hsa-miR-200c-3p | 0.139 | -0.125 | 9.160 | 1.13E-01 | 2.63E-01 | -0.364 | 0.132 | 0.198 | -0.096 | -0.368 | 0.165 | -0.034 | -0.641 | 0.045 | -0.130 | -0.130 | -0.279 | |
| 42507 | hsa-miR-202-5p | 0.215 | 1.200 | 5.800 | 3.83E-01 | 5.83E-01 | 1.054 | 1.039 | 0.644 | 1.953 | 1.426 | 1.728 | 1.824 | 0.983 | 0.727 | 0.993 | 1.034 | 0.989 | |
| 11004 | hsa-miR-203a | 0.098 | -0.218 | 7.208 | 2.20E-01 | 4.14E-01 | -1.506 | -1.318 | 0.483 | 1.255 | -0.430 | 0.500 | 0.260 | -1.675 | -1.452 | -0.550 | 0.306 | 1.509 | |
| 42502 | hsa-miR-204-3p | 0.144 | 0.142 | 5.607 | 9.48E-02 | 2.44E-01 | 0.042 | 0.135 | 0.672 | 0.280 | 0.125 | 0.033 | 0.241 | 0.062 | 0.138 | -0.207 | 0.000 | 0.187 | |
| 11005 | hsa-miR-204-5p | 0.097 | 0.381 | 5.727 | 1.93E-01 | 3.76E-01 | 0.482 | 0.360 | 0.416 | 0.460 | 0.507 | 0.350 | 0.143 | 0.480 | 0.351 | 0.209 | 0.419 | 0.391 | |
| 46917 | hsa-miR-205-5p | 0.255 | 1.035 | 5.856 | 5.68E-03 | 5.69E-02 | 1.354 | 1.457 | 0.991 | 0.979 | 1.152 | 1.042 | 0.915 | 0.902 | 1.373 | 0.774 | 0.696 | 0.787 | |
| 145845 | hsa-miR-20a-5p | 0.061 | 0.052 | 9.196 | 4.88E-01 | 6.79E-01 | 0.134 | 0.315 | -0.506 | 0.299 | -0.297 | 0.552 | -0.628 | -0.178 | 0.395 | -0.168 | 0.229 | 0.478 | |
| 42640 | hsa-miR-20b-5p | 0.205 | -0.019 | 5.786 | 3.79E-02 | 1.43E-01 | 0.057 | 0.292 | -0.338 | 0.134 | -0.188 | 0.545 | -0.621 | -0.352 | 0.189 | -0.297 | 0.087 | 0.263 | |
| 147506 | hsa-miR-21-5p | 0.375 | -0.078 | 8.786 | 2.00E-03 | 4.35E-02 | 0.562 | 0.590 | 0.232 | 0.046 | -0.867 | 0.092 | -0.027 | 0.312 | 0.105 | -0.919 | -0.605 | -0.462 | |
| 145852 | hsa-miR-210 | 1.505 | -1.200 | 7.133 | 9.05E-04 | 4.35E-02 | -0.009 | 0.930 | -2.404 | 0.028 | -1.177 | -0.051 | -2.712 | -1.828 | -1.820 | -1.472 | -1.809 | -2.076 | |
| 11011 | hsa-miR-211-5p | -0.004 | 1.235 | 5.974 | 9.87E-01 | 9.92E-01 | 0.924 | 0.969 | 0.735 | 1.963 | 1.487 | 1.318 | 1.403 | 1.756 | 1.325 | 1.023 | 0.995 | 0.917 | |
| 11020 | hsa-miR-22-3p | 0.190 | -0.292 | 7.657 | 7.47E-02 | 2.16E-01 | -0.634 | -0.063 | 0.196 | -0.261 | -0.232 | -0.188 | 0.115 | -0.526 | -0.282 | -0.240 | -0.510 | -0.879 | |
| 42532 | hsa-miR-22-5p | -0.051 | 0.398 | 5.702 | 6.48E-01 | 7.87E-01 | 0.128 | 0.269 | 0.278 | 0.393 | 0.665 | 0.503 | 0.564 | 0.686 | -0.013 | 0.647 | 0.444 | 0.217 | |
| 11022 | hsa-miR-221-3p | 0.059 | 0.193 | 7.412 | 3.76E-01 | 5.83E-01 | 0.510 | 0.460 | 0.553 | -0.026 | -0.231 | 0.067 | 0.480 | 0.273 | 0.352 | -0.126 | -0.033 | 0.034 | |
| 11023 | hsa-miR-222-3p | 0.216 | -0.539 | 8.560 | 4.20E-02 | 1.55E-01 | 0.282 | -0.207 | 0.201 | -0.645 | -1.446 | -0.775 | -0.059 | -0.105 | -0.331 | -1.205 | -1.135 | -1.048 | |
| 11024 | hsa-miR-223-3p | -0.218 | -0.522 | 5.741 | 6.11E-02 | 1.83E-01 | -0.848 | -1.038 | -0.655 | -0.480 | -0.380 | -0.385 | -0.521 | -0.557 | -0.319 | -0.107 | -0.402 | -0.570 | |
| 42744 | hsa-miR-23a-3p | 0.143 | -0.384 | 9.007 | 8.70E-02 | 2.31E-01 | -0.154 | -0.361 | 0.489 | -0.802 | -0.939 | -0.108 | 0.263 | -0.453 | -0.489 | -0.745 | -0.357 | -0.950 | |
| 169330 | hsa-miR-23b-3p | 0.132 | -0.106 | 7.482 | 5.35E-02 | 1.69E-01 | -0.524 | 0.030 | 0.652 | -0.790 | 0.006 | 0.385 | 0.676 | -0.596 | -0.116 | -0.249 | 0.168 | -0.917 | |
| 148217 | hsa-miR-23c | 0.121 | -0.611 | 5.427 | 1.10E-01 | 2.61E-01 | -1.085 | -0.522 | -0.278 | -0.934 | -0.427 | -0.059 | -0.145 | -1.097 | -0.772 | -0.627 | -0.267 | -1.123 | |
| 17506 | hsa-miR-24-3p | 0.079 | -0.189 | 8.389 | 1.92E-01 | 3.76E-01 | -0.354 | -0.018 | 0.562 | -0.751 | -0.406 | 0.075 | 0.427 | -0.395 | -0.075 | -0.361 | -0.097 | -0.869 | |
| 42682 | hsa-miR-25-3p | -0.023 | 0.275 | 6.632 | 8.12E-01 | 8.86E-01 | 0.724 | 0.510 | 0.473 | -0.363 | 0.152 | 0.088 | 0.226 | 0.433 | 0.813 | 0.128 | 0.211 | -0.090 | |
| 42523 | hsa-miR-26b-3p | 0.109 | 1.099 | 6.018 | 2.55E-01 | 4.57E-01 | 1.075 | 1.073 | 0.878 | 1.398 | 1.334 | 1.162 | 1.095 | 1.152 | 0.862 | 1.154 | 1.082 | 0.918 | |
| 146008 | hsa-miR-26b-5p | 0.099 | 0.413 | 7.415 | 2.85E-01 | 4.92E-01 | 0.203 | 0.697 | 0.368 | -0.037 | 0.580 | 0.963 | 0.285 | -0.031 | 0.380 | 0.434 | 0.799 | 0.309 | |
| 46483 | hsa-miR-27a-3p | 0.303 | -0.036 | 6.294 | 3.33E-03 | 4.83E-02 | 0.168 | 0.316 | 0.699 | -0.542 | -0.331 | 0.388 | 0.480 | -0.052 | -0.293 | -0.555 | -0.057 | -0.644 | |
| 147199 | hsa-miR-27b-3p | 0.285 | 0.175 | 7.182 | 4.13E-03 | 5.22E-02 | -0.416 | 0.581 | 1.228 | -0.644 | 0.671 | 0.487 | 0.881 | -0.592 | -0.006 | 0.329 | 0.374 | -0.790 | |
| 11038 | hsa-miR-299-5p | -0.064 | 0.504 | 5.740 | 3.97E-01 | 5.87E-01 | 0.370 | 0.401 | 0.405 | 0.711 | 0.370 | 0.572 | 0.345 | 0.655 | 0.609 | 0.366 | 0.676 | 0.562 | |
| 168687 | hsa-miR-29a-3p | 0.234 | -0.450 | 7.078 | 4.34E-03 | 5.22E-02 | -0.776 | -0.434 | -0.901 | -0.664 | 0.198 | 0.579 | -1.094 | -0.973 | -0.686 | -0.175 | 0.246 | -0.718 | |
| 145638 | hsa-miR-29a-5p | 0.281 | 0.080 | 5.812 | 1.59E-02 | 9.84E-02 | 0.034 | 0.288 | -0.101 | -0.420 | 0.819 | 0.705 | -0.382 | -0.295 | 0.112 | 0.183 | 0.292 | -0.270 | |
| 17810 | hsa-miR-29b-1-5p | 0.049 | 0.978 | 6.422 | 6.37E-01 | 7.83E-01 | 1.162 | 1.028 | 0.872 | 0.822 | 1.247 | 0.883 | 0.598 | 1.042 | 0.794 | 0.976 | 1.233 | 1.080 | |
| 11040 | hsa-miR-29b-3p | 0.401 | 0.048 | 6.192 | 1.24E-03 | 4.35E-02 | -0.046 | 0.533 | -0.327 | -0.049 | 0.509 | 0.870 | -0.591 | -0.118 | -0.130 | 0.010 | 0.260 | -0.344 | |
| 13143 | hsa-miR-301a-3p | 0.189 | 0.002 | 6.606 | 4.63E-02 | 1.58E-01 | 0.399 | 0.693 | 0.327 | -0.782 | 0.015 | -0.073 | 0.353 | 0.303 | 0.557 | -0.485 | -0.467 | -0.817 | |
| 27838 | hsa-miR-302d-3p | 0.278 | 0.845 | 5.825 | 5.21E-02 | 1.69E-01 | 1.173 | 1.265 | 0.848 | 0.614 | 1.109 | 0.893 | 0.736 | 0.714 | 0.421 | 0.679 | 0.890 | 0.793 | |
| 146112 | hsa-miR-30b-5p | 0.277 | -0.172 | 7.790 | 1.20E-02 | 8.55E-02 | -0.221 | 0.523 | -0.043 | -0.620 | -0.691 | 0.850 | -0.211 | -0.647 | 0.218 | -0.794 | 0.198 | -0.629 | |
| 42923 | hsa-miR-30c-5p | 0.081 | -0.193 | 7.165 | 3.04E-01 | 5.14E-01 | -0.107 | 0.051 | 0.217 | -0.695 | -0.561 | 0.177 | 0.138 | -0.133 | 0.015 | -0.870 | -0.057 | -0.498 | |
| 19596 | hsa-miR-30d-5p | 0.263 | 0.130 | 6.380 | 2.33E-03 | 4.35E-02 | 0.001 | 0.758 | 0.102 | 0.004 | -0.237 | 0.938 | -0.006 | -0.226 | 0.389 | -0.484 | 0.506 | -0.189 | |
| 28191 | hsa-miR-30e-5p | 0.320 | -0.223 | 5.724 | 5.89E-03 | 5.69E-02 | -0.122 | 0.165 | 0.126 | -0.235 | -0.657 | 0.347 | 0.052 | -0.255 | -0.151 | -0.922 | -0.402 | -0.620 | |
| 46320 | hsa-miR-31-3p | 0.052 | -0.466 | 5.737 | 4.58E-01 | 6.57E-01 | 0.623 | 1.419 | -0.351 | -1.697 | -0.994 | -1.642 | -0.493 | 0.446 | 1.306 | -1.095 | -1.634 | -1.484 | |
| 11052 | hsa-miR-31-5p | -0.039 | -0.752 | 6.640 | 7.10E-01 | 8.38E-01 | 0.535 | 1.524 | -0.230 | -2.725 | -1.273 | -2.462 | -0.397 | 0.271 | 1.462 | -1.341 | -2.053 | -2.340 | |
| 169380 | hsa-miR-3124-3p | 0.210 | -0.469 | 6.885 | 4.59E-03 | 5.22E-02 | -0.477 | -0.531 | 0.309 | -0.131 | -0.720 | -0.636 | 0.040 | -0.629 | -0.795 | -0.825 | -0.812 | -0.426 | |
| 146005 | hsa-miR-3129-5p | 0.373 | 0.819 | 5.649 | 3.21E-03 | 4.83E-02 | 0.989 | 1.176 | 1.283 | 0.903 | 1.004 | 0.677 | 0.614 | 0.551 | 0.665 | 0.515 | 0.695 | 0.755 | |
| 169232 | hsa-miR-3156-3p | -0.031 | -0.773 | 6.101 | 6.49E-01 | 7.87E-01 | -0.834 | -0.813 | -0.533 | -0.992 | -0.585 | -0.976 | -0.439 | -0.721 | -0.676 | -0.821 | -0.976 | -0.914 | |
| 146085 | hsa-miR-3170 | 0.090 | 0.064 | 5.648 | 2.27E-01 | 4.18E-01 | 0.088 | 0.018 | 0.342 | -0.097 | 0.173 | 0.129 | 0.054 | 0.039 | 0.097 | -0.097 | 0.125 | -0.102 | |
| 147891 | hsa-miR-3175 | 0.185 | -0.349 | 5.709 | 4.73E-02 | 1.58E-01 | 0.403 | -0.313 | -0.606 | -0.403 | -0.362 | -0.256 | -0.798 | -0.143 | -0.539 | -0.503 | -0.185 | -0.483 | |
| 147595 | hsa-miR-3178 | 0.352 | -0.761 | 5.863 | 1.85E-03 | 4.35E-02 | -1.469 | -1.108 | 1.252 | 0.128 | -1.440 | -0.872 | 0.818 | -1.746 | -1.756 | -1.486 | -1.137 | -0.312 | |
| 147667 | hsa-miR-3182 | 0.112 | -1.133 | 8.398 | 1.57E-01 | 3.29E-01 | -0.892 | -1.195 | -0.420 | -1.488 | -0.872 | -1.595 | -0.651 | -1.063 | -1.074 | -0.814 | -1.913 | -1.618 | |
| 29575 | hsa-miR-32-3p | 0.090 | 0.075 | 6.293 | 1.45E-01 | 3.15E-01 | 0.370 | 0.130 | 0.250 | -0.141 | 0.188 | -0.077 | 0.121 | 0.218 | 0.018 | 0.062 | -0.010 | -0.228 | |
| 11053 | hsa-miR-32-5p | 0.470 | 0.341 | 6.267 | 1.44E-02 | 9.22E-02 | 0.623 | 1.064 | 0.569 | -0.211 | 0.553 | 0.858 | 0.166 | 0.819 | -0.034 | -0.093 | 0.054 | -0.276 | |
| 27533 | hsa-miR-320a | 0.118 | -0.099 | 7.343 | 8.94E-02 | 2.35E-01 | 0.159 | -0.711 | -0.418 | 0.012 | 0.407 | 0.310 | -0.488 | 0.156 | -0.821 | 0.365 | 0.157 | -0.317 | |
| 46324 | hsa-miR-320b | 0.274 | -0.253 | 7.120 | 7.94E-03 | 6.32E-02 | 0.231 | -0.831 | -0.404 | -0.227 | 0.321 | 0.214 | -0.537 | -0.060 | -1.502 | 0.192 | 0.000 | -0.430 | |
| 169363 | hsa-miR-320c | 0.340 | -0.297 | 5.718 | 2.49E-03 | 4.35E-02 | -0.069 | -0.552 | -0.330 | -0.148 | 0.238 | 0.095 | -0.450 | -0.279 | -1.273 | -0.075 | -0.179 | -0.547 | |
| 11058 | hsa-miR-325 | 0.106 | 0.774 | 5.732 | 2.01E-01 | 3.86E-01 | 0.882 | 0.812 | 0.674 | 0.708 | 1.022 | 0.865 | 0.815 | 0.934 | 0.594 | 0.648 | 0.686 | 0.650 | |
| 42887 | hsa-miR-331-3p | 0.152 | -0.125 | 5.974 | 7.79E-02 | 2.19E-01 | 0.138 | 0.058 | 0.032 | -0.600 | 0.189 | -0.114 | -0.033 | 0.176 | -0.005 | -0.276 | -0.394 | -0.675 | |
| 145745 | hsa-miR-335-3p | 0.050 | -0.268 | 7.539 | 3.81E-01 | 5.83E-01 | -0.504 | -0.083 | 0.022 | -0.498 | -0.216 | -0.175 | -0.016 | -0.564 | -0.035 | -0.288 | -0.325 | -0.529 | |
| 11065 | hsa-miR-335-5p | 0.095 | 0.255 | 5.970 | 2.66E-01 | 4.71E-01 | 0.118 | 0.522 | 0.596 | -0.132 | 0.112 | 0.597 | 0.728 | 0.015 | 0.574 | -0.242 | 0.280 | -0.109 | |
| 42592 | hsa-miR-338-3p | 0.026 | 0.388 | 5.959 | 7.90E-01 | 8.75E-01 | 0.015 | 1.088 | -0.027 | 0.719 | 0.198 | 0.413 | 0.139 | 0.327 | 0.653 | 0.242 | 0.309 | 0.581 | |
| 42739 | hsa-miR-339-5p | 0.202 | -0.543 | 5.776 | 4.30E-02 | 1.55E-01 | -0.579 | -0.001 | -0.447 | -0.638 | -0.576 | -0.415 | -0.354 | -0.585 | -0.142 | -1.041 | -0.843 | -0.899 | |
| 145859 | hsa-miR-33a-5p | 0.550 | 0.168 | 6.386 | 1.08E-02 | 8.08E-02 | 0.230 | 1.271 | 0.022 | 0.333 | 0.377 | 0.424 | -0.206 | 0.457 | 0.036 | 0.018 | -0.567 | -0.382 | |
| 168586 | hsa-miR-34a-5p | 0.136 | 0.027 | 6.117 | 1.10E-01 | 2.61E-01 | 0.599 | 0.443 | 0.540 | -0.546 | -0.031 | -0.434 | 0.667 | 0.271 | 0.208 | -0.076 | -0.756 | -0.555 | |
| 11074 | hsa-miR-34c-5p | 0.182 | 0.795 | 5.685 | 8.49E-02 | 2.29E-01 | 0.421 | 1.188 | 1.078 | 0.712 | 0.997 | 0.920 | 0.555 | 0.659 | 0.915 | 0.688 | 0.742 | 0.663 | |
| 148420 | hsa-miR-3607-3p | 0.106 | -0.220 | 7.321 | 1.21E-01 | 2.73E-01 | 0.049 | -0.122 | -0.113 | -0.393 | -0.067 | -0.354 | -0.254 | -0.233 | -0.295 | -0.129 | -0.332 | -0.391 | |
| 14301 | hsa-miR-361-5p | 0.008 | 0.750 | 6.779 | 8.96E-01 | 9.34E-01 | 0.750 | 0.719 | 0.627 | 0.639 | 0.580 | 1.209 | 0.676 | 0.653 | 0.881 | 0.623 | 1.059 | 0.583 | |
| 148493 | hsa-miR-3613-3p | 0.065 | -1.378 | 9.035 | 5.74E-01 | 7.43E-01 | -1.062 | -1.262 | -0.896 | -1.657 | -1.668 | -1.529 | -1.557 | -0.794 | -1.239 | -1.539 | -1.668 | -1.665 | |
| 148413 | hsa-miR-3614-3p | 0.227 | 0.743 | 5.756 | 2.38E-02 | 1.08E-01 | 0.890 | 1.014 | 0.785 | 0.912 | 0.762 | 0.772 | 0.615 | 0.961 | 0.494 | 0.457 | 0.710 | 0.537 | |
| 148317 | hsa-miR-3621 | 0.202 | -0.386 | 5.881 | 5.70E-02 | 1.77E-01 | -0.777 | -0.632 | 0.721 | 0.222 | -0.787 | -0.459 | 0.319 | -0.746 | -1.226 | -0.745 | -0.552 | 0.027 | |
| 148481 | hsa-miR-3646 | 0.116 | -0.396 | 7.689 | 1.10E-01 | 2.61E-01 | -0.397 | -0.481 | 0.276 | -0.368 | -0.397 | -0.662 | 0.273 | -0.483 | -0.461 | -0.499 | -0.836 | -0.721 | |
| 148327 | hsa-miR-3651 | 0.058 | -0.052 | 6.231 | 3.86E-01 | 5.83E-01 | 0.030 | 0.089 | 0.227 | -0.127 | -0.246 | -0.111 | 0.189 | -0.183 | -0.111 | -0.159 | -0.154 | -0.067 | |
| 148377 | hsa-miR-3653 | 0.176 | -0.386 | 5.813 | 2.01E-02 | 1.01E-01 | -0.465 | 0.298 | -0.842 | -0.151 | -0.359 | -0.272 | -0.925 | -0.642 | 0.277 | -0.644 | -0.429 | -0.481 | |
| 11078 | hsa-miR-365a-3p/-365b-3p | 0.050 | 0.581 | 6.608 | 6.47E-01 | 7.87E-01 | 0.268 | 0.570 | 0.741 | 0.696 | 0.524 | 0.835 | 1.160 | 0.401 | 0.498 | 0.398 | 0.373 | 0.509 | |
| 148214 | hsa-miR-3675-3p | -0.143 | -0.748 | 5.882 | 1.19E-01 | 2.73E-01 | -0.997 | -1.054 | -0.968 | -0.609 | -0.540 | -0.751 | -0.854 | -0.752 | -0.547 | -0.487 | -0.686 | -0.735 | |
| 148410 | hsa-miR-3676-3p | -0.140 | 0.194 | 7.184 | 3.70E-02 | 1.42E-01 | -0.111 | -0.198 | 0.549 | 0.058 | 0.340 | 0.105 | 0.634 | 0.186 | -0.036 | 0.389 | 0.230 | 0.181 | |
| 168763 | hsa-miR-3676-5p | 0.198 | -0.562 | 5.522 | 1.13E-02 | 8.25E-02 | -0.006 | -0.231 | -0.076 | -1.177 | -0.267 | -1.023 | -0.278 | -0.331 | -0.474 | -0.560 | -1.127 | -1.194 | |
| 148038 | hsa-miR-3679-3p | -0.156 | 1.157 | 7.461 | 2.07E-01 | 3.93E-01 | 0.898 | 0.805 | 0.808 | 1.303 | 1.483 | 1.176 | 1.144 | 1.447 | 1.271 | 1.263 | 1.161 | 1.123 | |
| 148156 | hsa-miR-3686 | 0.137 | -0.634 | 6.370 | 3.06E-02 | 1.29E-01 | -0.602 | -0.739 | -0.535 | -0.329 | -0.700 | -0.485 | -0.675 | -0.712 | -0.782 | -0.868 | -0.656 | -0.522 | |
| 148282 | hsa-miR-3714 | 0.129 | 0.447 | 5.706 | 9.51E-02 | 2.44E-01 | 0.496 | 0.614 | 0.725 | 0.460 | 0.368 | 0.408 | 0.318 | 0.475 | 0.515 | 0.348 | 0.380 | 0.261 | |
| 168978 | hsa-miR-371b-5p | 0.328 | -1.313 | 7.590 | 5.94E-04 | 4.35E-02 | -2.371 | -1.585 | 0.443 | -0.016 | -2.227 | -1.136 | 0.140 | -2.533 | -1.984 | -2.436 | -1.590 | -0.460 | |
| 145844 | hsa-miR-374a-5p | 0.154 | 0.190 | 5.857 | 1.31E-01 | 2.88E-01 | 0.081 | 0.506 | 0.124 | 0.451 | -0.044 | 0.487 | 0.181 | 0.304 | 0.268 | -0.392 | 0.029 | 0.290 | |
| 42476 | hsa-miR-374b-3p | 0.078 | 0.459 | 5.636 | 2.50E-01 | 4.53E-01 | 0.641 | 0.682 | 0.563 | 0.388 | 0.286 | 0.426 | 0.410 | 0.543 | 0.412 | 0.289 | 0.498 | 0.367 | |
| 148098 | hsa-miR-374b-5p | 0.080 | -0.091 | 6.430 | 4.11E-01 | 6.03E-01 | -0.545 | 0.148 | 0.009 | 0.360 | -0.678 | 0.398 | 0.183 | -0.424 | 0.209 | -1.114 | 0.060 | 0.299 | |
| 148430 | hsa-miR-374c-5p | 0.149 | -0.029 | 6.283 | 3.17E-02 | 1.32E-01 | -0.387 | 0.469 | 0.266 | 0.178 | -0.472 | 0.221 | 0.107 | -0.478 | 0.206 | -0.675 | 0.038 | 0.184 | |
| 46918 | hsa-miR-375 | 0.023 | 0.494 | 6.056 | 7.00E-01 | 8.33E-01 | 0.171 | 1.191 | 0.142 | 0.522 | 0.697 | 0.312 | 0.015 | 0.138 | 1.150 | 0.615 | 0.426 | 0.555 | |
| 42899 | hsa-miR-377-5p | -0.037 | 0.616 | 5.675 | 7.71E-01 | 8.65E-01 | 0.307 | 0.431 | 0.585 | 0.902 | 0.876 | 0.486 | 0.864 | 0.873 | 0.574 | 0.565 | 0.440 | 0.494 | |
| 148668 | hsa-miR-378a-3p | 0.023 | 0.034 | 6.441 | 7.19E-01 | 8.42E-01 | -0.268 | 0.124 | 0.073 | 0.484 | -0.171 | 0.033 | 0.136 | -0.122 | 0.046 | -0.363 | -0.001 | 0.442 | |
| 147755 | hsa-miR-378c | -0.147 | 0.685 | 6.284 | 1.51E-01 | 3.20E-01 | 0.570 | 0.522 | 0.141 | 0.955 | 0.705 | 0.775 | 0.792 | 0.638 | 0.658 | 0.794 | 0.757 | 0.910 | |
| 168637 | hsa-miR-3940-5p | 0.448 | -1.557 | 7.123 | 7.55E-03 | 6.32E-02 | -2.627 | -1.626 | 1.068 | -0.576 | -2.732 | -1.508 | 0.551 | -2.818 | -2.733 | -2.709 | -1.804 | -1.175 | |
| 169024 | hsa-miR-3960 | 0.318 | -1.451 | 7.978 | 6.93E-04 | 4.35E-02 | -2.445 | -1.762 | 0.503 | -0.231 | -2.563 | -1.253 | 0.176 | -2.760 | -2.106 | -2.688 | -1.753 | -0.525 | |
| 42730 | hsa-miR-423-3p | 0.086 | -0.226 | 6.328 | 1.82E-01 | 3.66E-01 | 0.042 | -0.190 | 0.035 | -0.496 | -0.152 | -0.336 | 0.019 | 0.009 | -0.348 | -0.399 | -0.419 | -0.475 | |
| 27565 | hsa-miR-423-5p | 0.055 | -0.111 | 6.696 | 3.44E-01 | 5.54E-01 | -0.038 | -0.133 | 0.120 | -0.116 | -0.183 | -0.150 | -0.021 | -0.135 | -0.218 | -0.180 | -0.103 | -0.174 | |
| 17608 | hsa-miR-425-5p | 0.216 | -0.079 | 5.843 | 4.71E-03 | 5.22E-02 | -0.356 | -0.037 | -0.315 | 0.475 | 0.490 | -0.085 | -0.477 | -0.479 | -0.197 | 0.120 | -0.306 | 0.218 | |
| 147631 | hsa-miR-4258 | 0.130 | 0.354 | 5.898 | 5.72E-02 | 1.77E-01 | 0.313 | 0.461 | 0.518 | 0.592 | 0.365 | 0.265 | 0.485 | 0.247 | 0.310 | 0.164 | 0.225 | 0.304 | |
| 147751 | hsa-miR-4274 | -0.005 | 0.893 | 6.348 | 9.39E-01 | 9.54E-01 | 0.891 | 0.790 | 0.871 | 0.841 | 1.048 | 0.903 | 0.693 | 0.924 | 0.772 | 1.071 | 0.958 | 0.956 | |
| 147767 | hsa-miR-4279 | 0.010 | 0.261 | 7.081 | 8.81E-01 | 9.28E-01 | 0.005 | 0.028 | 0.710 | 0.597 | 0.183 | 0.069 | 0.581 | 0.155 | 0.080 | 0.199 | 0.076 | 0.446 | |
| 169129 | hsa-miR-4284 | -0.298 | -0.976 | 11.711 | 1.26E-01 | 2.81E-01 | -0.257 | -1.338 | -1.834 | -0.543 | -2.419 | -0.362 | -1.917 | -0.170 | -0.118 | -1.702 | -0.312 | -0.745 | |
| 169409 | hsa-miR-4286 | 0.259 | -0.728 | 9.763 | 1.88E-02 | 9.90E-02 | -0.573 | -0.373 | -0.143 | -1.283 | -0.873 | -0.344 | -0.448 | -1.078 | -0.715 | -0.670 | -0.722 | -1.510 | |
| 147588 | hsa-miR-4288 | 0.127 | -0.420 | 8.555 | 1.39E-01 | 3.04E-01 | 0.053 | 0.072 | 0.256 | -0.593 | -1.214 | -0.714 | 0.040 | -0.255 | 0.054 | -0.995 | -0.918 | -0.829 | |
| 147735 | hsa-miR-4289 | 0.104 | -0.079 | 5.744 | 1.06E-01 | 2.60E-01 | -0.213 | 0.153 | -0.111 | -0.081 | 0.129 | -0.034 | -0.092 | -0.462 | 0.069 | -0.007 | -0.160 | -0.131 | |
| 13171 | hsa-miR-429 | 0.084 | -0.005 | 7.304 | 2.39E-01 | 4.36E-01 | -1.718 | 1.231 | 0.002 | -0.108 | 0.041 | 0.776 | -0.091 | -1.721 | 0.986 | -0.108 | 0.614 | 0.041 | |
| 169282 | hsa-miR-4290 | 0.089 | -0.079 | 6.649 | 1.79E-01 | 3.66E-01 | -0.344 | -0.367 | 0.412 | 0.438 | -0.062 | -0.286 | 0.314 | -0.272 | -0.422 | -0.208 | -0.335 | 0.179 | |
| 147616 | hsa-miR-4291 | 0.052 | -0.451 | 5.941 | 4.61E-01 | 6.58E-01 | -0.561 | -0.331 | 0.026 | -0.892 | -0.448 | -0.340 | 0.003 | -0.749 | -0.126 | -0.554 | -0.355 | -1.079 | |
| 169407 | hsa-miR-4301 | 0.081 | -0.445 | 5.930 | 1.82E-01 | 3.66E-01 | -0.396 | -0.277 | -0.292 | -0.476 | -0.783 | -0.202 | -0.400 | -0.512 | -0.218 | -0.894 | -0.245 | -0.646 | |
| 147722 | hsa-miR-4306 | 0.091 | -0.219 | 5.880 | 1.12E-01 | 2.63E-01 | -0.345 | 0.421 | 0.053 | -0.170 | -0.397 | -0.601 | 0.016 | -0.441 | 0.305 | -0.520 | -0.675 | -0.271 | |
| 42674 | hsa-miR-431-3p | 0.014 | 0.651 | 5.953 | 8.82E-01 | 9.28E-01 | 0.546 | 0.491 | 0.471 | 0.751 | 0.919 | 0.772 | 0.780 | 0.778 | 0.102 | 0.786 | 0.731 | 0.686 | |
| 145705 | hsa-miR-431-5p | 0.382 | 0.645 | 5.807 | 1.64E-02 | 9.90E-02 | 1.068 | 1.107 | 1.298 | 0.542 | 0.535 | 0.465 | 0.391 | 0.660 | 0.328 | 0.364 | 0.504 | 0.480 | |
| 147907 | hsa-miR-4312 | -0.074 | 0.164 | 5.804 | 3.81E-01 | 5.83E-01 | 0.065 | -0.046 | 0.043 | 0.207 | 0.292 | 0.197 | 0.118 | 0.254 | 0.371 | 0.142 | 0.143 | 0.175 | |
| 168709 | hsa-miR-4429 | 0.332 | -0.603 | 5.626 | 7.28E-04 | 4.35E-02 | -0.443 | -0.964 | -0.614 | -0.456 | -0.026 | -0.118 | -0.768 | -0.823 | -1.349 | -0.223 | -0.466 | -0.988 | |
| 169171 | hsa-miR-4436b-5p | 0.067 | 0.384 | 5.713 | 3.85E-01 | 5.83E-01 | 0.218 | 0.352 | 0.397 | 0.383 | 0.632 | 0.522 | 0.435 | 0.414 | 0.288 | 0.335 | 0.317 | 0.312 | |
| 169188 | hsa-miR-4443 | 0.100 | -0.727 | 10.159 | 3.74E-01 | 5.83E-01 | -0.473 | -0.419 | -0.184 | -1.007 | -1.252 | -0.724 | -0.392 | -0.806 | -0.347 | -0.821 | -1.127 | -1.166 | |
| 169015 | hsa-miR-4454 | 0.034 | -0.700 | 14.716 | 8.24E-01 | 8.93E-01 | -0.691 | -0.169 | -0.279 | -1.091 | -1.487 | -0.380 | -0.338 | -1.174 | -0.197 | -0.642 | -0.674 | -1.280 | |
| 168919 | hsa-miR-4456 | -0.080 | -0.905 | 7.356 | 3.92E-01 | 5.86E-01 | 0.084 | -1.625 | 0.354 | -1.581 | -1.175 | -1.725 | 0.157 | -0.022 | -1.215 | -1.176 | -1.672 | -1.258 | |
| 169285 | hsa-miR-4467 | 0.254 | -1.065 | 7.019 | 1.47E-03 | 4.35E-02 | -1.669 | -1.474 | 0.328 | -0.127 | -1.709 | -0.978 | 0.158 | -1.902 | -1.679 | -2.064 | -1.323 | -0.341 | |
| 169395 | hsa-miR-4484 | 0.250 | -1.168 | 5.050 | 1.14E-01 | 2.63E-01 | -1.467 | -1.454 | -1.550 | 0.053 | -1.494 | -0.349 | -1.726 | -0.881 | -2.048 | -1.820 | -0.865 | -0.418 | |
| 169110 | hsa-miR-4497 | 0.136 | -0.750 | 5.747 | 5.84E-02 | 1.79E-01 | -1.387 | -1.273 | 1.126 | -0.143 | -1.375 | -1.037 | 1.037 | -1.483 | -1.480 | -1.398 | -1.094 | -0.491 | |
| 169385 | hsa-miR-4500 | 0.133 | -0.276 | 5.809 | 5.91E-02 | 1.79E-01 | -0.167 | -0.033 | -0.380 | -0.332 | -0.263 | -0.081 | -0.522 | -0.328 | -0.342 | -0.283 | -0.251 | -0.328 | |
| 42892 | hsa-miR-450b-3p | -0.026 | 0.978 | 5.890 | 8.43E-01 | 9.05E-01 | 1.054 | 0.506 | 0.772 | 0.976 | 1.209 | 1.274 | 1.183 | 1.163 | 1.019 | 0.887 | 0.903 | 0.789 | |
| 29379 | hsa-miR-452-5p | -0.035 | 0.219 | 6.037 | 5.98E-01 | 7.68E-01 | -0.485 | 0.686 | 1.132 | -0.280 | -0.161 | 0.314 | 1.421 | -0.464 | 0.679 | -0.163 | 0.232 | -0.287 | |
| 169159 | hsa-miR-4521 | -0.035 | -0.467 | 5.777 | 8.31E-01 | 8.95E-01 | -0.283 | -0.854 | -0.512 | -1.234 | 0.256 | -0.281 | -0.509 | -0.169 | -0.108 | -0.348 | -0.684 | -0.882 | |
| 168844 | hsa-miR-4532 | 0.048 | -1.483 | 7.230 | 5.60E-01 | 7.33E-01 | -1.535 | -1.658 | -1.114 | -1.362 | -1.644 | -1.441 | -1.097 | -1.479 | -1.394 | -1.880 | -1.710 | -1.483 | |
| 148620 | hsa-miR-454-3p | 0.046 | 0.249 | 5.541 | 6.03E-01 | 7.68E-01 | 0.061 | 0.426 | 0.429 | -0.051 | 0.426 | 0.341 | 0.486 | 0.317 | 0.313 | 0.146 | 0.031 | 0.063 | |
| 169102 | hsa-miR-4639-3p | -0.021 | -0.561 | 6.377 | 7.55E-01 | 8.54E-01 | -0.656 | -0.891 | -0.207 | -0.411 | -0.574 | -0.690 | -0.406 | -0.560 | -0.818 | -0.676 | -0.594 | -0.248 | |
| 46731 | hsa-miR-4657 | 0.048 | -0.407 | 7.418 | 5.24E-01 | 7.10E-01 | 0.258 | -0.514 | -1.097 | -0.347 | -0.189 | -0.411 | -1.077 | 0.139 | -0.337 | -0.503 | -0.484 | -0.323 | |
| 168798 | hsa-miR-4668-5p | 0.210 | -1.304 | 7.178 | 1.20E-01 | 2.73E-01 | -0.891 | -1.056 | -1.016 | -1.454 | -1.351 | -1.425 | -1.577 | -0.462 | -1.518 | -1.746 | -1.662 | -1.489 | |
| 169070 | hsa-miR-4695-3p | 0.085 | -0.821 | 8.788 | 2.70E-01 | 4.74E-01 | -0.655 | -0.809 | -0.353 | -1.280 | -0.736 | -0.839 | -0.514 | -0.977 | -0.806 | -0.567 | -1.009 | -1.312 | |
| 169028 | hsa-miR-4708-3p | 0.335 | -1.589 | 8.159 | 1.08E-03 | 4.35E-02 | -2.719 | -1.880 | 0.608 | -0.209 | -2.932 | -1.397 | 0.180 | -2.987 | -2.286 | -2.970 | -1.866 | -0.609 | |
| 169311 | hsa-miR-4714-5p | 0.156 | -0.736 | 6.317 | 4.38E-02 | 1.55E-01 | -0.616 | -0.665 | -0.614 | -0.250 | -0.889 | -0.915 | -0.787 | -0.818 | -0.872 | -1.129 | -0.790 | -0.487 | |
| 169323 | hsa-miR-4723-3p | -0.147 | 0.933 | 6.880 | 1.81E-01 | 3.66E-01 | 0.721 | 0.657 | 0.664 | 1.131 | 1.179 | 0.802 | 0.851 | 1.214 | 1.081 | 1.106 | 0.909 | 0.875 | |
| 169031 | hsa-miR-4726-5p | 0.104 | -1.221 | 5.927 | 1.65E-01 | 3.44E-01 | -1.412 | -1.269 | -1.022 | -0.785 | -1.339 | -1.186 | -1.050 | -1.318 | -1.262 | -1.588 | -1.478 | -0.943 | |
| 169182 | hsa-miR-4728-3p | 0.134 | -0.135 | 5.829 | 8.17E-02 | 2.26E-01 | -0.082 | -0.034 | 0.153 | 0.029 | -0.101 | -0.373 | 0.098 | -0.217 | -0.240 | -0.386 | -0.253 | -0.211 | |
| 169239 | hsa-miR-4732-5p | 0.262 | -0.807 | 5.647 | 8.02E-03 | 6.32E-02 | -1.142 | -1.281 | -0.964 | 0.686 | -1.197 | -0.156 | -1.046 | -1.345 | -1.800 | -1.235 | -0.464 | 0.266 | |
| 169130 | hsa-miR-4764-3p | 0.025 | -0.655 | 6.365 | 7.62E-01 | 8.59E-01 | -0.816 | -0.735 | -0.549 | -0.067 | -1.067 | -0.622 | -0.924 | -0.649 | -0.770 | -1.211 | -0.523 | 0.068 | |
| 168943 | hsa-miR-4769-3p | -0.070 | 0.207 | 5.795 | 4.72E-01 | 6.64E-01 | 0.057 | 0.089 | -0.031 | 0.464 | 0.289 | 0.162 | 0.358 | 0.369 | 0.188 | 0.218 | 0.158 | 0.159 | |
| 168915 | hsa-miR-4780 | 0.123 | -0.243 | 7.097 | 7.58E-02 | 2.16E-01 | 0.018 | -0.034 | -0.674 | -0.184 | -0.021 | -0.196 | -0.787 | -0.201 | -0.202 | -0.262 | -0.148 | -0.227 | |
| 169050 | hsa-miR-4787-5p | 0.387 | -1.399 | 8.201 | 9.59E-04 | 4.35E-02 | -2.395 | -1.570 | 0.575 | -0.187 | -2.530 | -1.125 | 0.162 | -2.678 | -2.166 | -2.609 | -1.742 | -0.523 | |
| 168995 | hsa-miR-4791 | -0.070 | -0.159 | 5.971 | 2.54E-01 | 4.57E-01 | -0.248 | -0.200 | -0.135 | -0.250 | -0.215 | -0.117 | 0.021 | -0.237 | -0.071 | -0.290 | -0.043 | -0.122 | |
| 169022 | hsa-miR-4797-5p | 0.066 | -0.966 | 5.963 | 3.32E-01 | 5.53E-01 | -1.267 | -0.905 | -0.559 | -1.534 | -0.636 | -0.699 | -0.517 | -1.235 | -0.952 | -0.933 | -0.815 | -1.543 | |
| 169313 | hsa-miR-4800-3p | 0.179 | -0.836 | 7.992 | 2.31E-02 | 1.08E-01 | -0.942 | -0.938 | -0.226 | -0.035 | -1.327 | -1.009 | -0.424 | -1.280 | -1.036 | -1.315 | -1.328 | -0.168 | |
| 148682 | hsa-miR-483-3p | 0.105 | 0.019 | 6.633 | 1.48E-01 | 3.20E-01 | 0.113 | -0.288 | 0.100 | 0.444 | 0.216 | -0.153 | -0.050 | -0.001 | -0.195 | 0.003 | -0.128 | 0.171 | |
| 147701 | hsa-miR-491-3p | 0.058 | -0.929 | 9.717 | 5.47E-01 | 7.31E-01 | -1.335 | -0.917 | -0.558 | -1.162 | -0.632 | -0.797 | -0.659 | -1.630 | -0.790 | -0.285 | -1.112 | -1.273 | |
| 148059 | hsa-miR-493-5p | -0.083 | -0.256 | 6.507 | 2.05E-01 | 3.92E-01 | -0.111 | -0.633 | -0.250 | -0.166 | -0.232 | -0.389 | -0.180 | -0.019 | -0.447 | -0.316 | -0.363 | 0.040 | |
| 42442 | hsa-miR-498 | 0.272 | 0.385 | 5.645 | 1.69E-02 | 9.90E-02 | 0.816 | 0.742 | 0.594 | 0.404 | 0.340 | 0.233 | 0.417 | 0.316 | 0.103 | 0.134 | 0.298 | 0.226 | |
| 14313 | hsa-miR-499a-5p | 0.270 | 0.780 | 5.801 | 2.91E-02 | 1.24E-01 | 1.164 | 1.314 | 0.933 | 0.387 | 0.596 | 1.098 | 0.525 | 0.520 | 0.895 | 0.367 | 0.961 | 0.606 | |
| 11134 | hsa-miR-502-5p | 0.334 | 0.535 | 5.824 | 3.31E-02 | 1.34E-01 | 0.811 | 0.885 | 1.335 | 0.510 | 0.477 | 0.190 | 0.382 | 0.514 | 0.381 | 0.087 | 0.430 | 0.412 | |
| 42490 | hsa-miR-505-5p | 0.095 | 0.549 | 5.828 | 2.56E-01 | 4.57E-01 | 0.568 | 0.716 | 0.595 | 0.649 | 0.579 | 0.474 | 0.585 | 0.630 | 0.287 | 0.409 | 0.574 | 0.528 | |
| 11140 | hsa-miR-508-3p | -0.042 | 0.774 | 5.987 | 5.59E-01 | 7.33E-01 | 0.613 | 0.658 | 0.657 | 0.774 | 0.998 | 0.821 | 0.741 | 0.846 | 0.777 | 0.778 | 0.814 | 0.817 | |
| 11141 | hsa-miR-509-3p | 0.159 | 0.817 | 5.875 | 6.52E-02 | 1.91E-01 | 0.840 | 0.717 | 0.804 | 0.999 | 1.091 | 0.927 | 0.733 | 0.947 | 0.559 | 0.934 | 0.704 | 0.548 | |
| 168878 | hsa-miR-5100 | 0.091 | -0.580 | 14.448 | 6.35E-01 | 7.83E-01 | -0.421 | -0.169 | -0.343 | -1.084 | -0.679 | -0.512 | -0.562 | -1.155 | -0.248 | 0.330 | -0.859 | -1.262 | |
| 145690 | hsa-miR-512-5p | 0.326 | 0.601 | 5.602 | 7.62E-02 | 2.16E-01 | 1.015 | 1.646 | 1.173 | 0.710 | 0.108 | -0.066 | 0.341 | 0.745 | 0.688 | 0.094 | 0.330 | 0.429 | |
| 42581 | hsa-miR-513a-5p | 0.196 | 0.183 | 5.890 | 5.06E-02 | 1.66E-01 | 0.218 | 0.262 | 0.323 | 0.371 | 0.335 | 0.177 | 0.067 | 0.214 | -0.365 | 0.277 | 0.121 | 0.195 | |
| 145717 | hsa-miR-516a-3p/-516b-3p | 0.101 | 1.070 | 5.723 | 5.43E-01 | 7.29E-01 | 0.969 | 0.802 | 0.721 | 1.252 | 1.448 | 1.528 | 1.572 | 0.711 | 0.805 | 1.006 | 1.112 | 0.910 | |
| 46221 | hsa-miR-519d | 0.007 | 0.617 | 5.887 | 9.21E-01 | 9.48E-01 | 0.465 | 0.648 | 0.649 | 0.556 | 0.703 | 0.701 | 0.498 | 0.734 | 0.693 | 0.585 | 0.616 | 0.552 | |
| 46744 | hsa-miR-526b-5p | 0.368 | 0.945 | 5.809 | 2.34E-03 | 4.35E-02 | 1.037 | 1.280 | 1.483 | 0.864 | 1.043 | 1.066 | 0.772 | 0.629 | 0.934 | 0.534 | 0.911 | 0.789 | |
| 14271 | hsa-miR-539-5p | 0.378 | 0.666 | 5.667 | 5.01E-03 | 5.36E-02 | 1.472 | 0.756 | 0.978 | 0.600 | 0.568 | 0.752 | 0.732 | 0.504 | 0.503 | 0.290 | 0.468 | 0.364 | |
| 168951 | hsa-miR-548as-3p | 0.017 | -1.046 | 5.416 | 8.90E-01 | 9.30E-01 | -1.046 | -1.439 | -0.966 | -0.652 | -1.197 | -0.922 | -1.099 | -0.757 | -1.279 | -1.801 | -1.085 | -0.303 | |
| 46705 | hsa-miR-548k | -0.083 | 0.504 | 5.825 | 3.00E-01 | 5.11E-01 | 0.431 | 0.164 | 0.302 | 0.634 | 0.690 | 0.556 | 0.407 | 0.609 | 0.551 | 0.650 | 0.522 | 0.536 | |
| 168933 | hsa-miR-5581-3p | 0.059 | -0.916 | 7.806 | 3.20E-01 | 5.38E-01 | -1.166 | -0.398 | -0.694 | -1.095 | -1.182 | -0.785 | -0.762 | -1.296 | -0.418 | -1.221 | -0.933 | -1.042 | |
| 169169 | hsa-miR-5684 | 0.031 | -0.810 | 7.743 | 6.11E-01 | 7.72E-01 | -0.878 | -1.308 | -0.173 | -0.806 | -0.779 | -0.823 | -0.208 | -0.985 | -1.152 | -0.870 | -0.897 | -0.840 | |
| 169376 | hsa-miR-5701 | 0.159 | -0.468 | 8.260 | 4.79E-02 | 1.59E-01 | 0.089 | -0.566 | -0.643 | -0.159 | -0.120 | -0.932 | -0.836 | -0.263 | -0.696 | -0.006 | -1.086 | -0.400 | |
| 169211 | hsa-miR-5704 | -0.042 | -0.410 | 6.632 | 7.41E-01 | 8.47E-01 | -0.632 | -0.723 | -0.621 | -0.147 | -0.029 | -0.436 | -0.492 | -0.276 | -0.429 | -0.681 | -0.333 | -0.128 | |
| 17490 | hsa-miR-571 | 0.391 | 0.463 | 5.631 | 7.04E-03 | 6.11E-02 | 0.907 | 1.031 | 0.961 | 0.481 | 0.282 | 0.289 | 0.433 | 0.391 | 0.132 | 0.151 | 0.306 | 0.192 | |
| 27740 | hsa-miR-574-5p | -0.027 | 0.513 | 6.188 | 7.32E-01 | 8.46E-01 | 0.353 | 0.510 | 0.541 | 0.396 | 0.616 | 0.585 | 0.635 | 0.650 | 0.652 | 0.512 | 0.389 | 0.323 | |
| 17302 | hsa-miR-578 | 0.204 | 0.948 | 5.855 | 4.54E-02 | 1.58E-01 | 1.150 | 1.339 | 1.255 | 0.705 | 0.934 | 0.915 | 0.777 | 0.701 | 1.329 | 0.663 | 0.801 | 0.801 | |
| 14962 | hsa-miR-581 | 0.266 | 0.553 | 5.819 | 2.33E-02 | 1.08E-01 | 0.731 | 1.021 | 0.913 | 0.388 | 0.527 | 0.539 | 0.461 | 0.512 | 0.312 | 0.344 | 0.472 | 0.422 | |
| 145647 | hsa-miR-584-5p | 0.063 | 0.215 | 6.054 | 3.28E-01 | 5.48E-01 | 0.219 | 0.327 | 0.342 | 0.082 | -0.080 | 0.592 | 0.324 | 0.312 | 0.266 | -0.206 | 0.358 | 0.049 | |
| 42567 | hsa-miR-590-3p | 0.010 | 0.843 | 5.662 | 9.37E-01 | 9.54E-01 | 1.034 | 0.687 | 0.544 | 1.048 | 0.497 | 1.280 | 0.798 | 1.162 | 0.852 | 0.756 | 0.620 | 0.844 | |
| 17503 | hsa-miR-590-5p | 0.169 | 0.255 | 5.568 | 6.34E-02 | 1.88E-01 | 0.423 | 0.763 | 0.350 | -0.015 | 0.324 | 0.194 | 0.206 | 0.446 | 0.473 | -0.074 | -0.117 | 0.092 | |
| 17312 | hsa-miR-592 | 0.198 | 0.746 | 5.693 | 2.27E-01 | 4.18E-01 | 0.490 | 0.450 | 0.798 | 1.105 | 1.084 | 1.143 | 1.048 | 0.942 | 0.287 | 0.550 | 0.703 | 0.352 | |
| 42504 | hsa-miR-593-3p | 0.289 | 0.656 | 5.969 | 2.81E-02 | 1.22E-01 | 0.862 | 0.981 | 1.031 | 0.489 | 0.804 | 0.632 | 0.334 | 0.643 | 0.368 | 0.497 | 0.662 | 0.564 | |
| 17349 | hsa-miR-595 | 0.092 | 0.572 | 5.718 | 2.20E-01 | 4.14E-01 | 0.577 | 0.431 | 0.586 | 0.510 | 0.775 | 0.827 | 0.341 | 0.453 | 0.525 | 0.545 | 0.666 | 0.622 | |
| 17336 | hsa-miR-618 | 0.069 | 0.369 | 5.737 | 3.65E-01 | 5.77E-01 | 0.519 | 0.422 | 0.473 | 0.463 | 0.359 | 0.184 | 0.249 | 0.558 | 0.264 | 0.161 | 0.401 | 0.372 | |
| 46556 | hsa-miR-623 | 0.231 | 0.547 | 5.744 | 6.57E-03 | 6.03E-02 | 0.895 | 0.692 | 0.708 | 0.601 | 0.632 | 0.447 | 0.510 | 0.575 | 0.270 | 0.449 | 0.424 | 0.364 | |
| 17961 | hsa-miR-629-5p | 0.048 | 0.318 | 5.740 | 5.67E-01 | 7.38E-01 | 0.272 | 0.381 | 0.371 | 0.322 | 0.446 | 0.263 | 0.162 | 0.538 | 0.182 | 0.205 | 0.380 | 0.299 | |
| 42591 | hsa-miR-634 | 0.216 | -0.309 | 5.933 | 1.26E-02 | 8.59E-02 | -0.300 | -0.145 | 0.785 | -0.282 | -0.630 | -0.636 | 0.474 | -0.448 | -0.611 | -0.847 | -0.673 | -0.396 | |
| 169034 | hsa-miR-642b-5p | 0.122 | 0.257 | 6.732 | 9.17E-02 | 2.39E-01 | 0.150 | 0.076 | 0.772 | 0.467 | 0.468 | -0.027 | 0.620 | 0.166 | -0.113 | 0.233 | 0.036 | 0.234 | |
| 169375 | hsa-miR-660-3p | 0.158 | -0.675 | 5.586 | 8.49E-02 | 2.29E-01 | -0.944 | -0.890 | -0.091 | -0.101 | -0.733 | -0.817 | -0.070 | -0.862 | -1.055 | -0.902 | -1.021 | -0.614 | |
| 145973 | hsa-miR-664a-3p | 0.016 | 0.305 | 6.247 | 8.26E-01 | 8.93E-01 | 0.209 | 0.118 | 0.419 | 0.363 | 0.469 | 0.300 | 0.299 | 0.275 | 0.375 | 0.309 | 0.221 | 0.304 | |
| 168882 | hsa-miR-664b-3p | 0.156 | -0.567 | 7.364 | 2.64E-02 | 1.16E-01 | -0.706 | -0.405 | 0.284 | -0.640 | -0.560 | -0.907 | 0.120 | -1.012 | -0.578 | -0.670 | -0.917 | -0.814 | |
| 29490 | hsa-miR-7-5p | 0.028 | 0.195 | 8.433 | 7.55E-01 | 8.54E-01 | 0.675 | 0.930 | 0.884 | -0.333 | -0.627 | -0.274 | 0.660 | 0.495 | 0.828 | -0.425 | -0.452 | -0.016 | |
| 146064 | hsa-miR-718 | -0.105 | 0.909 | 6.170 | 4.63E-01 | 6.58E-01 | 0.639 | 0.693 | 0.617 | 0.930 | 1.177 | 1.084 | 1.108 | 1.310 | 0.909 | 0.929 | 0.763 | 0.753 | |
| 27568 | hsa-miR-744-5p | 0.329 | -0.483 | 5.664 | 3.46E-03 | 4.83E-02 | 0.545 | -0.328 | -0.320 | -0.999 | -0.037 | -0.771 | -0.547 | 0.273 | -1.022 | -0.495 | -1.023 | -1.072 | |
| 146111 | hsa-miR-767-5p | 0.083 | -0.237 | 6.323 | 1.86E-01 | 3.66E-01 | -0.132 | -0.227 | -0.312 | -0.301 | 0.179 | -0.376 | -0.425 | -0.127 | -0.407 | -0.020 | -0.381 | -0.310 | |
| 28884 | hsa-miR-876-3p | 0.092 | 0.743 | 5.702 | 3.39E-01 | 5.54E-01 | 0.735 | 0.915 | 0.594 | 0.712 | 0.876 | 0.905 | 0.838 | 0.791 | 0.547 | 0.504 | 0.772 | 0.734 | |
| 148622 | hsa-miR-877-3p | 0.021 | 0.484 | 6.093 | 7.99E-01 | 8.78E-01 | 0.349 | 0.339 | 0.445 | 0.768 | 0.628 | 0.434 | 0.492 | 0.568 | 0.322 | 0.491 | 0.539 | 0.429 | |
| 30033 | hsa-miR-877-5p | -0.037 | 0.369 | 6.142 | 7.22E-01 | 8.43E-01 | 0.290 | 0.413 | 0.362 | 0.114 | 0.679 | 0.244 | 0.480 | 0.654 | 0.036 | 0.493 | 0.255 | 0.405 | |
| 46259 | hsa-miR-885-5p | 0.004 | 0.686 | 5.863 | 9.68E-01 | 9.78E-01 | 0.429 | 0.630 | 0.605 | 0.844 | 0.888 | 0.731 | 0.791 | 0.848 | 0.552 | 0.618 | 0.626 | 0.668 | |
| 28047 | hsa-miR-890 | -0.070 | 1.070 | 5.856 | 6.25E-01 | 7.80E-01 | 0.717 | 0.963 | 0.844 | 1.293 | 1.433 | 0.957 | 1.295 | 1.324 | 1.171 | 1.027 | 0.882 | 0.929 | |
| 29852 | hsa-miR-9-3p | -0.057 | -0.062 | 5.274 | 5.68E-01 | 7.38E-01 | 0.221 | -0.344 | 1.265 | -0.624 | -0.585 | -0.477 | 1.779 | 0.096 | -0.367 | -0.420 | -0.725 | -0.567 | |
| 4040 | hsa-miR-9-5p | -0.100 | 0.779 | 6.248 | 1.06E-01 | 2.60E-01 | 1.059 | 0.242 | 1.825 | 0.378 | 0.538 | 0.333 | 1.979 | 1.261 | 0.242 | 0.604 | 0.395 | 0.492 | |
| 145693 | hsa-miR-92a-3p | 0.089 | -0.117 | 7.751 | 3.37E-01 | 5.54E-01 | -0.253 | 0.020 | -0.481 | 0.429 | -0.268 | 0.117 | -0.795 | -0.689 | -0.056 | -0.148 | 0.186 | 0.530 | |
| 30687 | hsa-miR-93-5p | 0.075 | -0.018 | 8.194 | 3.04E-01 | 5.14E-01 | 0.098 | 0.670 | -0.006 | -0.584 | -0.226 | 0.167 | -0.153 | -0.111 | 0.541 | -0.108 | -0.032 | -0.468 | |
| 42696 | hsa-miR-943 | 0.315 | 0.775 | 6.046 | 1.48E-02 | 9.31E-02 | 1.106 | 1.259 | 1.293 | 0.833 | 0.617 | 0.487 | 0.563 | 0.795 | 0.760 | 0.376 | 0.656 | 0.553 | |
| 13147 | hsa-miR-96-5p | 0.242 | 0.414 | 6.655 | 1.41E-02 | 9.22E-02 | 1.050 | 0.661 | 0.329 | -0.191 | 0.639 | 0.721 | 0.248 | 0.990 | 0.164 | 0.368 | 0.260 | -0.270 | |
| 11182 | hsa-miR-98-5p | -0.141 | 0.737 | 6.972 | 1.13E-01 | 2.63E-01 | 0.741 | 0.969 | 0.695 | 0.298 | 0.654 | 0.644 | 0.708 | 0.766 | 1.327 | 0.632 | 0.673 | 0.742 | |
| 17898 | hsa-miR-99b-3p | -0.032 | 0.482 | 5.810 | 6.59E-01 | 7.93E-01 | 0.596 | 0.704 | 0.495 | 0.363 | 0.334 | 0.300 | 0.385 | 0.617 | 0.515 | 0.418 | 0.557 | 0.496 | |
| 11184 | hsa-miR-99b-5p | 0.080 | -0.988 | 5.494 | 6.02E-01 | 7.68E-01 | 0.012 | 0.751 | -0.461 | -2.744 | -2.618 | -0.630 | -0.434 | -0.308 | 0.641 | -2.205 | -1.445 | -2.416 | |
| 28302 | hsa-miRPlus-A1015 | 0.151 | -0.492 | 6.791 | 4.39E-02 | 1.55E-01 | -0.454 | -0.243 | -0.642 | -0.311 | -0.398 | -0.450 | -0.615 | -0.560 | -0.352 | -0.758 | -0.658 | -0.459 | |
| 42492 | hsa-miRPlus-A1031 | 0.226 | 0.425 | 5.668 | 3.33E-02 | 1.34E-01 | 0.676 | 0.530 | 0.629 | 0.552 | 0.537 | 0.306 | 0.578 | 0.465 | -0.143 | 0.430 | 0.290 | 0.254 | |
| 17858 | hsa-miRPlus-A1073 | -0.005 | 0.881 | 6.151 | 9.36E-01 | 9.54E-01 | 0.848 | 0.797 | 0.812 | 0.784 | 0.991 | 1.038 | 0.780 | 0.931 | 0.883 | 0.881 | 0.898 | 0.927 | |
| 169416 | hsa-miRPlus-A1086 | -0.296 | -1.311 | 6.460 | 5.37E-02 | 1.69E-01 | -1.639 | -1.950 | -0.635 | -1.652 | 0.173 | -3.050 | -0.795 | -1.752 | -1.518 | 0.417 | -2.598 | -0.732 | |
| 17848 | hsa-miRPlus-A1087 | -0.091 | -0.978 | 6.720 | 2.81E-01 | 4.87E-01 | -1.425 | -1.537 | -0.818 | -0.494 | -0.275 | -1.590 | -0.892 | -1.288 | -1.284 | -0.138 | -1.283 | -0.708 | |
| 146113 | hsa-miRPlus-G1246-3p | -0.053 | -0.173 | 7.042 | 3.92E-01 | 5.86E-01 | -0.102 | -0.269 | 0.162 | -0.267 | -0.384 | -0.336 | 0.229 | -0.132 | -0.146 | -0.488 | -0.221 | -0.121 | |
| 147975 | hsa-miRPlus-J1003 | 0.055 | 0.661 | 5.673 | 7.38E-01 | 8.47E-01 | 0.656 | 0.441 | 0.305 | 0.804 | 0.897 | 1.027 | 0.648 | 1.294 | 0.515 | 0.464 | 0.369 | 0.509 | |
| 147576 | hsv1-miR-H1-3p | -0.079 | 0.516 | 5.882 | 3.57E-01 | 5.67E-01 | 0.337 | 0.321 | 0.299 | 0.644 | 0.669 | 0.592 | 0.505 | 0.650 | 0.600 | 0.533 | 0.490 | 0.557 | |
| 146058 | hsv1-miR-H3-3p | 0.323 | 0.379 | 5.682 | 3.53E-02 | 1.38E-01 | 0.748 | 0.712 | 1.011 | 0.252 | 0.239 | 0.282 | 0.123 | 0.287 | 0.111 | 0.175 | 0.204 | 0.406 | |
| 146117 | hsv1-miR-H6-3p | 0.105 | -0.105 | 6.176 | 1.57E-01 | 3.29E-01 | -0.102 | -0.045 | 0.278 | 0.107 | -0.192 | -0.360 | -0.058 | -0.275 | -0.152 | -0.269 | -0.246 | 0.054 | |
| 146090 | hsv1-miR-H7-3p | 0.214 | 0.127 | 5.845 | 4.59E-03 | 5.22E-02 | 0.168 | 0.254 | 0.490 | 0.421 | 0.058 | 0.015 | 0.187 | -0.090 | -0.015 | -0.108 | -0.057 | 0.205 | |
| 146042 | hsv1-miR-H8-3p | -0.006 | 0.319 | 6.092 | 9.32E-01 | 9.54E-01 | 0.251 | 0.238 | 0.501 | 0.465 | 0.321 | 0.121 | 0.345 | 0.330 | 0.330 | 0.288 | 0.273 | 0.365 | |
| 42656 | kshv-miR-K12-10a-3p | -0.197 | 0.641 | 6.033 | 4.28E-02 | 1.55E-01 | 0.280 | 0.260 | 0.328 | 0.792 | 0.822 | 0.775 | 0.704 | 0.759 | 0.549 | 0.897 | 0.819 | 0.712 | |
| 42624 | kshv-miR-K12-10b | -0.181 | 1.151 | 6.216 | 1.74E-01 | 3.58E-01 | 0.819 | 0.814 | 0.800 | 1.318 | 1.325 | 1.289 | 1.315 | 1.392 | 1.199 | 1.382 | 1.151 | 1.010 | |
| 19011 | SNORD10 | -0.085 | 0.119 | 6.544 | 1.85E-01 | 3.66E-01 | -0.060 | 0.115 | -0.200 | 0.167 | 0.204 | 0.234 | -0.025 | 0.111 | 0.183 | 0.357 | 0.186 | 0.156 | |
| 145666 | SNORD110 | 0.291 | 0.060 | 5.638 | 1.84E-02 | 9.90E-02 | 0.333 | 0.488 | 0.474 | -0.025 | 0.034 | -0.072 | -0.221 | 0.015 | -0.032 | -0.208 | -0.098 | 0.030 | |
| 19005 | SNORD118 | 0.080 | -0.503 | 5.623 | 2.68E-01 | 4.72E-01 | -0.438 | -0.654 | -0.775 | -0.304 | -0.200 | -0.406 | -1.086 | -0.517 | -0.551 | -0.209 | -0.413 | -0.482 | |
| 19606 | SNORD12 | 0.110 | 0.385 | 5.744 | 2.95E-01 | 5.07E-01 | 0.364 | 0.672 | 0.589 | 0.180 | 0.414 | 0.417 | 0.091 | 0.171 | 0.470 | 0.339 | 0.382 | 0.527 | |
| 19603 | SNORD13 | -0.061 | -0.161 | 6.990 | 3.36E-01 | 5.54E-01 | 0.369 | -0.683 | -0.712 | -0.053 | 0.438 | -0.507 | -0.729 | 0.359 | -0.495 | 0.394 | -0.331 | 0.016 | |
| 19607 | SNORD15A | 0.263 | 0.467 | 5.685 | 2.08E-03 | 4.35E-02 | 0.801 | 0.590 | 0.687 | 0.494 | 0.505 | 0.516 | 0.470 | 0.470 | 0.240 | 0.110 | 0.331 | 0.392 | |
| 19008 | SNORD2 | 0.114 | -0.428 | 7.705 | 8.04E-02 | 2.24E-01 | -0.380 | -0.424 | -0.312 | -0.067 | -0.508 | -0.535 | -0.529 | -0.567 | -0.532 | -0.491 | -0.576 | -0.214 | |
| 19007 | SNORD3@ | 0.083 | -1.292 | 9.584 | 4.91E-01 | 6.79E-01 | -0.414 | -1.142 | -1.886 | -1.309 | -1.626 | -1.123 | -2.239 | -0.784 | -1.131 | -1.111 | -1.435 | -1.300 | |
| 46204 | SNORD38B | -0.019 | -0.134 | 6.663 | 7.48E-01 | 8.52E-01 | -0.102 | -0.129 | -0.205 | -0.323 | 0.045 | -0.145 | -0.181 | -0.155 | -0.102 | -0.046 | -0.085 | -0.176 | |
| 46206 | SNORD44 | 0.039 | -0.169 | 6.893 | 5.55E-01 | 7.33E-01 | 0.211 | -0.133 | -0.293 | -0.388 | 0.030 | -0.326 | -0.362 | 0.132 | -0.093 | -0.137 | -0.458 | -0.214 | |
| 46205 | SNORD48 | -0.042 | -0.047 | 6.491 | 5.10E-01 | 6.96E-01 | -0.157 | 0.060 | -0.293 | -0.351 | 0.523 | -0.188 | -0.262 | -0.058 | 0.146 | 0.365 | -0.163 | -0.183 | |
| 46203 | SNORD49A | -0.022 | -0.368 | 7.029 | 7.36E-01 | 8.47E-01 | -0.420 | -0.566 | -0.699 | -0.550 | 0.102 | -0.140 | -0.713 | -0.426 | -0.383 | -0.005 | -0.209 | -0.409 | |
| 19604 | SNORD4A | 0.110 | -0.336 | 7.429 | 1.02E-01 | 2.57E-01 | -0.142 | -0.304 | -0.442 | -0.222 | -0.344 | -0.234 | -0.549 | -0.459 | -0.391 | -0.393 | -0.304 | -0.251 | |
| 19605 | SNORD6 | 0.032 | -0.622 | 7.761 | 6.20E-01 | 7.80E-01 | -1.121 | -0.543 | -0.320 | -0.485 | -0.750 | -0.420 | -0.379 | -1.059 | -0.462 | -0.776 | -0.432 | -0.719 | |
| 145663 | SNORD68 | 0.139 | -0.737 | 9.383 | 1.50E-01 | 3.20E-01 | -0.429 | -0.757 | -0.408 | -0.592 | -1.083 | -0.738 | -0.540 | -0.770 | -0.785 | -0.814 | -0.983 | -0.951 | |
| 17492 | sv40-miR-S1-5p | 0.140 | -0.366 | 5.970 | 4.71E-02 | 1.58E-01 | -0.487 | -0.549 | 0.015 | 0.375 | -0.527 | -0.603 | -0.125 | -0.595 | -0.894 | -0.624 | -0.622 | 0.244 | |

**Supplementary Table S2. MicroRNA profiling for colorectal cancer cell lines at 20.9% versus 0.2% oxygen.** LogFC, Log2(FoldChange); AvEx, average expression level calculated based on all samples included in the comparison; AvH3, Log2 (average Hy3 hybridized fluorescent tag signal intensity; p-value calculated from t-statistics comparing the two sample groups; adj.P.Val, adjusted p-value from t-statistics comparing the two sample groups.

|  |  |  |  |  |  |  | **0.2% oxygen** | | | | | | **normoxia** | | | | | |  |
| --- | --- | --- | --- | --- | --- | --- | --- | --- | --- | --- | --- | --- | --- | --- | --- | --- | --- | --- | --- |
| **ProbeID** | **Annotation** | **logFC** | **AvEx** | **AvH3** | **P.Value** | **adj.P.Val** | **VACO** | **HCT116** | **HT29** | **DLD1** | **HT55** | **SW837** | **SW837** | **HCT116** | **HT29** | **DLD1** | **HT55** | **VACO** | |
| 42485 | ebv-miR-BART10-5p | 0.443 | 0.804 | 5.778 | 3.27E-03 | 1.78E-01 | 1.233 | 1.364 | 1.129 | 1.014 | 0.921 | 0.491 | 0.469 | 0.748 | 0.828 | 0.391 | 0.546 | 0.514 | |
| 17306 | ebv-miR-BART12 | -0.362 | 0.756 | 5.755 | 1.97E-02 | 1.78E-01 | 0.608 | 0.376 | 0.453 | 0.782 | 0.512 | 0.716 | 1.552 | 1.026 | 0.870 | 0.894 | 0.718 | 0.562 | |
| 42897 | ebv-miR-BART15 | 0.011 | 0.645 | 5.836 | 9.12E-01 | 9.55E-01 | 0.679 | 0.485 | 0.770 | 0.511 | 0.874 | 0.585 | 0.606 | 0.702 | 0.633 | 0.648 | 0.691 | 0.556 | |
| 42522 | ebv-miR-BART19-3p | 0.206 | 0.957 | 6.858 | 2.53E-01 | 4.69E-01 | 2.214 | 0.767 | 0.854 | 0.816 | 0.944 | 0.765 | 0.536 | 0.913 | 0.903 | 0.744 | 1.008 | 1.022 | |
| 145990 | ebv-miR-BART21-3p | -0.152 | 0.739 | 5.741 | 3.55E-01 | 5.75E-01 | 0.925 | 0.598 | 0.518 | 0.760 | 0.605 | 0.571 | 1.468 | 1.008 | 0.479 | 0.772 | 0.659 | 0.506 | |
| 42610 | hcmv-miR-UL36-3p | 0.494 | 0.579 | 5.591 | 1.12E-02 | 1.78E-01 | 1.186 | 1.298 | 0.461 | 0.813 | 0.765 | 0.433 | 0.288 | 0.544 | 0.600 | 0.093 | 0.397 | 0.071 | |
| 17619 | hcmv-miR-US25-2-5p | -0.250 | 0.706 | 5.757 | 1.29E-01 | 3.73E-01 | 0.662 | 0.324 | 0.848 | 0.582 | 0.504 | 0.567 | 1.148 | 1.226 | 0.864 | 0.730 | 0.646 | 0.372 | |
| 147162 | hsa-let-7a-5p | -0.034 | -0.002 | 7.676 | 8.69E-01 | 9.39E-01 | -1.323 | 0.006 | 0.587 | 0.310 | 0.358 | -0.049 | 0.113 | -0.235 | 0.230 | 0.183 | -0.087 | -0.112 | |
| 147165 | hsa-let-7b-5p | 0.255 | -0.435 | 8.696 | 1.12E-01 | 3.42E-01 | 0.137 | -1.587 | 0.263 | -0.039 | -0.031 | -0.590 | -0.633 | -1.686 | -0.316 | 0.346 | -0.610 | -0.475 | |
| 145820 | hsa-let-7c | 0.114 | -0.023 | 6.654 | 3.14E-01 | 5.54E-01 | -0.055 | -0.465 | 0.455 | 0.434 | 0.028 | -0.193 | -0.196 | -0.571 | -0.081 | 0.510 | -0.120 | -0.023 | |
| 145968 | hsa-let-7d-5p | -0.051 | 0.089 | 7.260 | 7.51E-01 | 8.61E-01 | -0.830 | 0.168 | 0.633 | 0.247 | 0.103 | 0.058 | 0.122 | 0.033 | 0.360 | 0.044 | 0.021 | 0.105 | |
| 145846 | hsa-let-7e-5p | 0.153 | -0.231 | 6.801 | 2.09E-01 | 4.29E-01 | -1.134 | 0.274 | 1.208 | -1.367 | 0.074 | 0.017 | 0.082 | 0.220 | 0.747 | -1.239 | -0.383 | -1.270 | |
| 17752 | hsa-let-7f-5p | -0.240 | 0.365 | 6.109 | 2.01E-01 | 4.17E-01 | -0.077 | 0.239 | 0.862 | -0.402 | 0.355 | 0.491 | 0.529 | 0.329 | 0.322 | 0.588 | 0.599 | 0.543 | |
| 46438 | hsa-let-7g-5p | 0.000 | 0.135 | 7.871 | 9.98E-01 | 1.00E+00 | -0.956 | 0.501 | 0.474 | 0.278 | 0.587 | -0.071 | -0.162 | 0.355 | -0.078 | 0.258 | 0.389 | 0.046 | |
| 9938 | hsa-let-7i-5p | 0.003 | 0.184 | 7.452 | 9.82E-01 | 9.95E-01 | -0.647 | 0.217 | 1.213 | 0.127 | 0.068 | 0.136 | 0.125 | 0.338 | 0.680 | 0.127 | 0.005 | -0.180 | |
| 31026 | hsa-miR-101-3p | 0.172 | -0.058 | 6.389 | 3.31E-01 | 5.70E-01 | -0.783 | 0.421 | 1.209 | -0.131 | -0.457 | -0.090 | 0.127 | 0.274 | 0.330 | -0.643 | -0.670 | -0.280 | |
| 10919 | hsa-miR-103a-3p | 0.116 | 0.075 | 7.925 | 3.69E-01 | 5.87E-01 | -0.226 | 0.225 | 0.925 | -0.110 | 0.213 | -0.230 | -0.173 | -0.134 | 0.429 | -0.083 | -0.025 | 0.088 | |
| 46801 | hsa-miR-106a-5p | -0.063 | -0.024 | 7.885 | 7.37E-01 | 8.54E-01 | -0.575 | 0.110 | 0.434 | -0.001 | 0.392 | -0.693 | -0.549 | -0.195 | 0.323 | -0.225 | 0.128 | 0.562 | |
| 17854 | hsa-miR-106b-3p | 0.098 | 0.408 | 6.425 | 2.99E-01 | 5.34E-01 | 0.422 | 0.545 | 0.880 | 0.125 | 0.470 | 0.303 | 0.088 | 0.459 | 0.812 | 0.021 | 0.517 | 0.259 | |
| 19582 | hsa-miR-106b-5p | 0.172 | -0.006 | 8.395 | 1.68E-01 | 3.90E-01 | -0.699 | 0.135 | 1.124 | -0.235 | 0.268 | -0.113 | 0.026 | -0.108 | 0.542 | -0.364 | -0.039 | -0.611 | |
| 10923 | hsa-miR-107 | -0.027 | 0.348 | 6.853 | 8.53E-01 | 9.28E-01 | -0.239 | 0.476 | 0.837 | 0.483 | 0.267 | 0.184 | 0.232 | 0.258 | 0.438 | 0.451 | 0.313 | 0.477 | |
| 28019 | hsa-miR-10a-3p | 0.325 | -0.211 | 5.464 | 3.30E-02 | 2.17E-01 | -0.180 | -0.572 | 0.218 | 1.360 | -0.453 | -0.663 | -0.997 | -1.081 | -0.009 | 0.476 | -0.399 | -0.229 | |
| 13485 | hsa-miR-10a-5p | -0.122 | -0.342 | 8.056 | 5.38E-01 | 7.31E-01 | -0.853 | -2.247 | 0.459 | 1.086 | 0.229 | -1.092 | -1.347 | -2.194 | 0.069 | 1.184 | 0.201 | 0.396 | |
| 42969 | hsa-miR-10b-3p | -0.203 | 0.527 | 5.828 | 1.16E-01 | 3.46E-01 | 0.231 | 0.448 | 0.833 | 0.109 | 0.538 | 0.393 | 0.686 | 0.643 | 0.548 | 0.576 | 0.643 | 0.676 | |
| 10925 | hsa-miR-10b-5p | -0.075 | 0.492 | 5.860 | 5.73E-01 | 7.52E-01 | 0.279 | 0.172 | 0.605 | 0.748 | 0.474 | 0.445 | 0.152 | 0.222 | 0.453 | 0.957 | 0.461 | 0.930 | |
| 42848 | hsa-miR-1180 | 0.587 | 0.761 | 5.700 | 5.35E-03 | 1.78E-01 | 1.401 | 1.743 | 0.955 | 1.014 | 0.701 | 0.512 | 0.488 | 0.508 | 0.612 | 0.209 | 0.544 | 0.443 | |
| 46345 | hsa-miR-1207-3p | -0.260 | 1.213 | 6.193 | 3.96E-02 | 2.27E-01 | 1.076 | 0.807 | 1.114 | 1.260 | 1.147 | 1.095 | 1.383 | 1.428 | 1.553 | 1.351 | 1.182 | 1.161 | |
| 46531 | hsa-miR-1231 | 0.060 | 0.586 | 5.670 | 6.56E-01 | 8.15E-01 | 0.594 | 0.570 | 0.918 | 0.010 | 0.991 | 0.617 | 0.645 | 0.726 | 0.392 | 0.443 | 0.695 | 0.437 | |
| 46624 | hsa-miR-1236-3p | 0.057 | 0.800 | 5.826 | 6.68E-01 | 8.15E-01 | 0.875 | 1.167 | 1.016 | 0.672 | 0.557 | 0.680 | 0.719 | 0.490 | 1.202 | 0.831 | 0.652 | 0.735 | |
| 46850 | hsa-miR-1237-3p | -0.186 | 0.793 | 5.921 | 1.95E-01 | 4.10E-01 | 0.837 | 0.580 | 0.688 | 0.671 | 0.847 | 0.575 | 1.154 | 1.232 | 0.759 | 0.775 | 0.666 | 0.726 | |
| 46690 | hsa-miR-1238-3p | -0.098 | 0.525 | 5.692 | 3.77E-01 | 5.96E-01 | 0.703 | 0.227 | 0.630 | 0.203 | 0.621 | 0.472 | 0.675 | 0.643 | 0.418 | 0.339 | 0.683 | 0.685 | |
| 42898 | hsa-miR-124-5p | 0.074 | 0.528 | 5.793 | 5.64E-01 | 7.48E-01 | 0.859 | 0.202 | 0.561 | 0.559 | 0.670 | 0.543 | 0.381 | 0.735 | 0.305 | 0.416 | 0.575 | 0.536 | |
| 168870 | hsa-miR-1246 | 0.615 | -0.721 | 11.392 | 4.58E-02 | 2.45E-01 | 2.183 | -0.915 | -0.987 | -1.823 | 0.475 | -1.412 | -1.143 | -1.652 | -1.640 | -1.369 | -0.665 | 0.302 | |
| 145977 | hsa-miR-1247-5p | 0.289 | -0.096 | 5.702 | 4.28E-02 | 2.37E-01 | 0.552 | -0.210 | -0.175 | 0.160 | 0.384 | -0.424 | -0.631 | -0.609 | -0.543 | -0.610 | 0.535 | 0.414 | |
| 46427 | hsa-miR-1248 | 0.506 | 0.693 | 5.847 | 4.30E-03 | 1.78E-01 | 1.447 | 1.438 | 1.217 | 0.656 | 0.574 | 0.347 | 0.308 | 0.579 | 0.670 | 0.178 | 0.419 | 0.488 | |
| 46210 | hsa-miR-1249 | 0.054 | 0.967 | 6.456 | 5.71E-01 | 7.52E-01 | 1.019 | 1.051 | 1.156 | 1.010 | 0.958 | 0.769 | 0.805 | 1.124 | 0.944 | 0.994 | 0.908 | 0.865 | |
| 10928 | hsa-miR-125a-5p | 0.140 | -0.187 | 6.661 | 3.69E-01 | 5.87E-01 | -1.522 | 0.519 | 1.450 | -1.624 | 0.432 | 0.043 | 0.221 | 0.232 | 0.807 | -1.265 | -0.131 | -1.405 | |
| 30787 | hsa-miR-125b-5p | 0.117 | -0.747 | 6.107 | 4.14E-01 | 6.38E-01 | -1.786 | 2.267 | -1.181 | -0.648 | -1.711 | -1.073 | -1.215 | 1.955 | -1.899 | -0.658 | -1.512 | -1.502 | |
| 4610 | hsa-miR-126-3p | 0.098 | 0.211 | 5.668 | 3.56E-01 | 5.75E-01 | 0.060 | 0.954 | 0.165 | -0.015 | 0.150 | 0.247 | 0.395 | 0.797 | -0.213 | -0.125 | -0.006 | 0.123 | |
| 169412 | hsa-miR-1260a | 0.435 | -0.713 | 8.225 | 1.69E-02 | 1.78E-01 | -0.444 | -0.450 | -0.249 | -0.738 | -0.270 | -0.821 | -0.550 | -0.859 | -0.840 | -0.938 | -1.176 | -1.218 | |
| 168619 | hsa-miR-1260b | 0.322 | -0.642 | 13.308 | 1.85E-01 | 4.07E-01 | -0.224 | -0.292 | -0.422 | -1.381 | -0.431 | -0.134 | 0.017 | -0.960 | -0.593 | -0.605 | -1.285 | -1.388 | |
| 46732 | hsa-miR-1264 | 0.177 | -0.330 | 8.101 | 1.92E-01 | 4.07E-01 | -0.182 | -0.524 | -0.086 | -0.205 | -0.183 | -0.268 | -0.034 | -0.412 | -0.401 | -0.287 | -0.765 | -0.612 | |
| 168925 | hsa-miR-1273g-3p | 0.202 | -0.770 | 7.422 | 1.68E-01 | 3.90E-01 | -0.295 | -0.922 | -0.993 | -0.799 | -0.408 | -0.595 | -0.323 | -0.875 | -1.135 | -0.944 | -1.081 | -0.865 | |
| 169082 | hsa-miR-1275 | 0.410 | -0.534 | 6.884 | 2.99E-02 | 2.09E-01 | 0.398 | -0.670 | -0.320 | -0.621 | -0.214 | -0.546 | -0.463 | -0.698 | -1.280 | -0.787 | -0.599 | -0.608 | |
| 46634 | hsa-miR-1281 | -0.127 | 0.529 | 5.865 | 2.46E-01 | 4.64E-01 | 0.285 | 0.518 | 0.520 | 0.731 | 0.364 | 0.377 | 0.648 | 0.752 | 0.699 | 0.473 | 0.496 | 0.488 | |
| 46368 | hsa-miR-1282 | 0.015 | 0.957 | 6.148 | 8.86E-01 | 9.47E-01 | 1.120 | 0.842 | 1.019 | 0.917 | 1.029 | 0.858 | 0.856 | 1.140 | 0.806 | 0.981 | 1.042 | 0.869 | |
| 46440 | hsa-miR-1287 | -0.085 | 0.612 | 5.790 | 3.90E-01 | 6.10E-01 | 0.727 | 0.408 | 0.701 | 0.382 | 0.597 | 0.603 | 0.683 | 0.750 | 0.757 | 0.446 | 0.619 | 0.672 | |
| 168568 | hsa-miR-1290 | 0.229 | -0.359 | 5.679 | 7.00E-02 | 2.76E-01 | 1.002 | -0.580 | -0.568 | -0.870 | 0.235 | -0.688 | -0.614 | -0.752 | -0.917 | -0.845 | -0.147 | 0.433 | |
| 46416 | hsa-miR-1293 | 0.011 | 0.696 | 5.775 | 9.16E-01 | 9.55E-01 | 0.863 | 0.572 | 0.817 | 0.424 | 0.995 | 0.540 | 0.711 | 0.784 | 0.700 | 0.568 | 0.735 | 0.643 | |
| 46215 | hsa-miR-1301 | -0.029 | 0.436 | 5.686 | 8.21E-01 | 9.06E-01 | 0.572 | 0.424 | 0.395 | 0.391 | 0.362 | 0.380 | 0.563 | 0.823 | -0.159 | 0.437 | 0.489 | 0.550 | |
| 46223 | hsa-miR-1306-3p | -0.367 | 0.599 | 5.714 | 6.76E-02 | 2.75E-01 | 0.595 | 0.100 | 0.172 | 0.628 | 0.584 | 0.413 | 0.864 | 1.439 | 0.669 | 0.658 | 0.481 | 0.585 | |
| 10936 | hsa-miR-130b-3p | -0.109 | 0.215 | 6.666 | 2.83E-01 | 5.11E-01 | -0.472 | 0.397 | 0.092 | 0.239 | -0.152 | 0.857 | 0.860 | 0.659 | 0.056 | 0.228 | -0.018 | -0.170 | |
| 42839 | hsa-miR-135a-5p | -0.045 | 0.732 | 6.052 | 6.62E-01 | 8.15E-01 | 0.706 | 0.312 | 0.811 | 0.440 | 0.906 | 1.081 | 1.002 | 0.661 | 0.766 | 0.580 | 0.840 | 0.674 | |
| 145914 | hsa-miR-135b-5p | -0.019 | -0.173 | 5.603 | 8.88E-01 | 9.47E-01 | -0.705 | -0.606 | 0.341 | -0.786 | 0.300 | 0.362 | 0.807 | -0.622 | 0.033 | -0.605 | -0.128 | -0.468 | |
| 10943 | hsa-miR-136-5p | 0.563 | 0.377 | 5.597 | 1.92E-02 | 1.78E-01 | 1.481 | 1.271 | 0.890 | 0.177 | 0.167 | -0.035 | 0.196 | 0.465 | -0.181 | -0.067 | 0.070 | 0.095 | |
| 148278 | hsa-miR-138-2-3p | 0.038 | -0.185 | 6.072 | 6.91E-01 | 8.28E-01 | -0.349 | -0.357 | 0.048 | -0.235 | 0.045 | -0.149 | -0.145 | -0.252 | -0.071 | -0.350 | -0.157 | -0.252 | |
| 10946 | hsa-miR-141-3p | 0.641 | -0.427 | 9.333 | 4.29E-03 | 1.78E-01 | 0.346 | -0.486 | 0.308 | -0.480 | 0.214 | -0.542 | -0.450 | -0.893 | -0.657 | -0.758 | -0.974 | -0.756 | |
| 10947 | hsa-miR-142-3p | 0.047 | 0.716 | 6.296 | 6.81E-01 | 8.28E-01 | 0.322 | 0.121 | 0.825 | 1.446 | 1.273 | 0.450 | 0.388 | 0.425 | 0.596 | 1.057 | 1.232 | 0.455 | |
| 42641 | hsa-miR-145-5p | -0.033 | 0.485 | 5.942 | 7.50E-01 | 8.61E-01 | 0.253 | 0.749 | 0.263 | 0.388 | 0.750 | 0.408 | 0.383 | 0.995 | 0.313 | 0.303 | 0.534 | 0.482 | |
| 146072 | hsa-miR-1469 | 0.116 | -0.413 | 5.617 | 3.45E-01 | 5.73E-01 | -0.004 | -0.693 | -0.324 | -1.174 | -0.319 | 0.386 | 0.480 | -0.765 | -0.704 | -0.879 | -0.600 | -0.355 | |
| 146052 | hsa-miR-1471 | -0.038 | 0.713 | 5.764 | 7.99E-01 | 8.90E-01 | 0.790 | 0.469 | 0.644 | 0.636 | 0.864 | 0.761 | 0.829 | 1.178 | 0.125 | 0.742 | 0.750 | 0.765 | |
| 10954 | hsa-miR-147a | 0.234 | 0.908 | 5.905 | 3.31E-02 | 2.17E-01 | 1.177 | 1.179 | 1.210 | 0.826 | 0.942 | 0.817 | 0.485 | 1.036 | 0.813 | 0.753 | 0.818 | 0.840 | |
| 19585 | hsa-miR-148b-3p | -0.022 | 0.543 | 6.278 | 8.42E-01 | 9.25E-01 | 0.240 | 0.708 | 0.857 | 0.473 | 0.435 | 0.480 | 0.635 | 0.742 | 0.503 | 0.480 | 0.380 | 0.588 | |
| 42810 | hsa-miR-149-5p | 0.494 | 0.519 | 5.588 | 3.24E-03 | 1.78E-01 | 1.066 | 1.490 | 0.827 | 0.498 | 0.385 | 0.332 | 0.208 | 0.580 | 0.397 | -0.013 | 0.217 | 0.246 | |
| 17463 | hsa-miR-151a-3p | 0.180 | 0.273 | 6.388 | 1.58E-01 | 3.83E-01 | 0.234 | 0.514 | 1.648 | -0.030 | -0.132 | -0.053 | -0.194 | 0.230 | 1.022 | 0.086 | -0.026 | -0.017 | |
| 11260 | hsa-miR-151a-5p | 0.005 | 0.134 | 6.470 | 9.67E-01 | 9.94E-01 | -0.664 | 0.605 | 1.345 | -0.165 | -0.099 | -0.202 | -0.142 | 0.313 | 1.050 | -0.253 | -0.047 | -0.133 | |
| 168871 | hsa-miR-151a-5p/-151b | -0.061 | -0.250 | 6.412 | 7.30E-01 | 8.54E-01 | -1.504 | 0.340 | 1.273 | -0.764 | -0.327 | -0.705 | -0.320 | 0.144 | 0.923 | -1.044 | -0.485 | -0.536 | |
| 10964 | hsa-miR-155-5p | -0.218 | 0.426 | 6.055 | 9.70E-02 | 3.18E-01 | 0.824 | -0.061 | 0.368 | -0.268 | 0.072 | 0.966 | 1.238 | 0.225 | 0.204 | 0.409 | 0.263 | 0.868 | |
| 27720 | hsa-miR-15a-5p | -0.052 | 0.308 | 7.413 | 7.60E-01 | 8.68E-01 | 0.072 | -0.887 | 0.798 | 0.612 | 0.767 | 0.332 | 0.001 | -0.635 | 0.419 | 0.502 | 0.722 | 0.995 | |
| 17280 | hsa-miR-15b-5p | -0.163 | 0.276 | 7.811 | 3.50E-01 | 5.74E-01 | -0.852 | -0.193 | 0.852 | 0.346 | 0.352 | 0.663 | 0.661 | -0.104 | 0.628 | 0.350 | 0.326 | 0.287 | |
| 10967 | hsa-miR-16-5p | -0.037 | -0.091 | 9.085 | 8.68E-01 | 9.39E-01 | -1.102 | -0.176 | 0.438 | -0.069 | 0.363 | -0.110 | 0.029 | -0.591 | 0.087 | -0.069 | -0.109 | 0.216 | |
| 169336 | hsa-miR-17-5p | -0.064 | -0.168 | 7.990 | 7.35E-01 | 8.54E-01 | -0.676 | 0.018 | 0.344 | -0.289 | 0.264 | -0.862 | -0.864 | -0.343 | 0.161 | -0.398 | 0.127 | 0.498 | |
| 42865 | hsa-miR-181a-5p | 0.139 | 0.041 | 6.162 | 1.53E-01 | 3.83E-01 | -0.287 | -0.637 | 0.759 | 0.330 | 1.080 | -0.581 | -0.853 | -0.776 | 0.609 | 0.203 | 0.915 | -0.270 | |
| 10972 | hsa-miR-181b-5p | 0.081 | -0.146 | 5.505 | 4.00E-01 | 6.24E-01 | -0.314 | -0.321 | 0.188 | -0.005 | 0.278 | -0.457 | -0.596 | -0.380 | 0.149 | -0.226 | 0.129 | -0.195 | |
| 169408 | hsa-miR-181d | 0.315 | -0.241 | 5.376 | 8.36E-03 | 1.78E-01 | -0.345 | -0.317 | 0.545 | -0.224 | 0.336 | -0.492 | -0.745 | -0.657 | -0.027 | -0.370 | -0.017 | -0.574 | |
| 10975 | hsa-miR-182-5p | 0.187 | -0.230 | 6.280 | 3.32E-01 | 5.70E-01 | -1.547 | 0.862 | 0.242 | 0.162 | 0.144 | -0.679 | -0.265 | 0.243 | -0.571 | -0.374 | 0.074 | -1.047 | |
| 46266 | hsa-miR-1825 | 0.211 | 0.453 | 5.769 | 6.35E-02 | 2.72E-01 | 0.827 | 0.701 | 0.823 | 0.279 | 0.427 | 0.295 | 0.230 | 0.386 | 0.440 | 0.247 | 0.379 | 0.407 | |
| 10977 | hsa-miR-183-5p | 0.229 | -0.110 | 6.228 | 6.24E-02 | 2.70E-01 | -0.772 | 0.709 | 0.230 | 0.163 | 0.065 | -0.366 | -0.289 | 0.188 | -0.031 | -0.264 | -0.163 | -0.786 | |
| 42902 | hsa-miR-185-5p | -0.092 | 0.153 | 6.539 | 3.46E-01 | 5.73E-01 | -0.427 | 0.219 | 0.849 | 0.012 | -0.281 | 0.270 | 0.341 | 0.251 | 0.976 | -0.049 | -0.206 | -0.119 | |
| 18739 | hsa-miR-186-5p | 0.137 | 0.072 | 5.863 | 1.84E-01 | 4.07E-01 | -0.018 | 0.688 | 0.818 | -0.229 | -0.205 | -0.212 | -0.173 | 0.506 | 0.436 | -0.250 | -0.324 | -0.174 | |
| 145670 | hsa-miR-18b-5p | 0.004 | 0.111 | 6.441 | 9.80E-01 | 9.95E-01 | -0.282 | 1.003 | 0.607 | -0.410 | 0.171 | -0.413 | -0.519 | 0.607 | 0.517 | -0.433 | 0.093 | 0.390 | |
| 27536 | hsa-miR-190a | 0.001 | 0.651 | 6.105 | 9.92E-01 | 1.00E+00 | 0.764 | 0.310 | 0.708 | 0.469 | 1.040 | 0.617 | 0.383 | 0.556 | 0.388 | 0.593 | 0.939 | 1.042 | |
| 42705 | hsa-miR-191-3p | -0.156 | 0.651 | 5.714 | 1.90E-01 | 4.07E-01 | 0.686 | 0.692 | 0.245 | 0.616 | 0.573 | 0.627 | 0.798 | 0.687 | 0.874 | 0.693 | 0.622 | 0.701 | |
| 10985 | hsa-miR-191-5p | 0.102 | -0.118 | 7.593 | 3.43E-01 | 5.73E-01 | 0.077 | 0.248 | -0.173 | 0.234 | -0.025 | -0.762 | -0.636 | 0.043 | -0.465 | 0.055 | -0.242 | 0.233 | |
| 146103 | hsa-miR-1913 | -0.109 | 0.630 | 6.146 | 3.10E-01 | 5.50E-01 | 0.451 | 0.490 | 0.827 | 0.446 | 0.751 | 0.486 | 0.704 | 0.741 | 0.686 | 0.656 | 0.627 | 0.692 | |
| 17946 | hsa-miR-192-3p | 0.335 | 0.159 | 5.680 | 1.19E-02 | 1.78E-01 | 0.753 | 0.399 | 0.311 | -0.144 | 0.667 | -0.025 | -0.477 | -0.152 | 0.120 | -0.083 | 0.274 | 0.270 | |
| 17732 | hsa-miR-192-5p | 0.202 | -1.296 | 7.357 | 2.59E-01 | 4.76E-01 | 0.143 | -3.642 | 0.677 | -2.480 | 1.095 | -2.962 | -3.683 | -3.690 | 0.123 | -2.343 | 0.517 | 0.695 | |
| 10986 | hsa-miR-193a-3p | 0.209 | -0.657 | 5.591 | 9.21E-02 | 3.16E-01 | 0.189 | -0.690 | -0.679 | -0.986 | -0.387 | -0.763 | -0.745 | -0.673 | -1.064 | -1.135 | -1.004 | 0.049 | |
| 46443 | hsa-miR-193a-5p | -0.268 | 0.411 | 5.722 | 4.87E-02 | 2.45E-01 | 0.213 | -0.048 | 0.210 | 0.327 | 0.368 | 0.592 | 0.629 | 0.740 | 0.389 | 0.568 | 0.386 | 0.556 | |
| 10987 | hsa-miR-193b-3p | 0.940 | -0.654 | 5.960 | 7.49E-03 | 1.78E-01 | -0.734 | 0.121 | 0.184 | -1.512 | 0.672 | 0.164 | 0.295 | -1.147 | -0.734 | -1.484 | -1.632 | -2.043 | |
| 10988 | hsa-miR-194-5p | 0.279 | -0.979 | 7.059 | 1.34E-01 | 3.83E-01 | 0.543 | -2.721 | 0.494 | -2.263 | 1.039 | -2.126 | -3.160 | -2.721 | -0.021 | -1.912 | 0.506 | 0.599 | |
| 10990 | hsa-miR-196a-5p | -0.088 | 0.339 | 6.790 | 4.10E-01 | 6.36E-01 | 0.060 | -0.344 | 0.693 | 0.532 | 0.630 | 0.199 | 0.136 | -0.237 | 0.529 | 0.580 | 0.880 | 0.411 | |
| 145889 | hsa-miR-196b-5p | 0.015 | 0.491 | 5.857 | 8.83E-01 | 9.47E-01 | 0.431 | 0.513 | 0.800 | 0.444 | 0.483 | 0.323 | 0.450 | 0.576 | 0.489 | 0.561 | 0.600 | 0.226 | |
| 146140 | hsa-miR-1976 | -0.191 | 0.683 | 5.765 | 1.71E-01 | 3.90E-01 | 0.734 | 0.595 | 0.690 | 0.381 | 0.652 | 0.475 | 0.808 | 1.298 | 0.861 | 0.661 | 0.521 | 0.525 | |
| 10997 | hsa-miR-19a-3p | 0.042 | -0.057 | 7.863 | 7.66E-01 | 8.69E-01 | -0.259 | 0.582 | 0.402 | -0.436 | -0.055 | -0.449 | -0.475 | 0.372 | 0.037 | -0.598 | -0.221 | 0.419 | |
| 10998 | hsa-miR-19b-3p | 0.151 | -0.122 | 7.276 | 1.45E-01 | 3.83E-01 | 0.327 | -0.186 | 0.226 | -0.225 | 0.175 | -0.596 | -0.768 | -0.502 | -0.017 | -0.404 | 0.089 | 0.419 | |
| 168819 | hsa-miR-200a-3p | -0.144 | -0.735 | 7.586 | 4.35E-01 | 6.56E-01 | -1.776 | -3.388 | 0.939 | -0.512 | 0.641 | -0.746 | -0.658 | -2.967 | 0.637 | -0.763 | 0.468 | -0.698 | |
| 147186 | hsa-miR-200b-3p | -0.066 | -0.471 | 8.582 | 5.39E-01 | 7.31E-01 | -0.719 | -3.222 | 0.978 | -0.419 | 0.938 | -0.580 | -0.589 | -3.007 | 0.810 | -0.318 | 0.832 | -0.353 | |
| 145974 | hsa-miR-200b-5p | 0.173 | 0.088 | 5.666 | 9.55E-02 | 3.18E-01 | 0.074 | -0.357 | 0.796 | 0.017 | 0.486 | 0.030 | -0.229 | -0.466 | 0.399 | -0.083 | 0.416 | -0.027 | |
| 17427 | hsa-miR-200c-3p | 0.354 | -0.018 | 9.160 | 2.62E-02 | 1.97E-01 | 0.443 | -0.311 | 0.419 | -0.186 | 0.659 | -0.070 | -0.034 | -0.641 | 0.045 | -0.130 | -0.130 | -0.279 | |
| 42507 | hsa-miR-202-5p | -0.322 | 0.931 | 5.800 | 4.74E-02 | 2.45E-01 | 0.855 | 0.551 | 0.557 | 0.914 | 0.982 | 0.756 | 1.824 | 0.983 | 0.727 | 0.993 | 1.034 | 0.989 | |
| 11004 | hsa-miR-203a | 0.029 | -0.253 | 7.208 | 8.81E-01 | 9.47E-01 | 0.413 | -1.384 | -1.023 | -0.467 | 0.704 | 0.326 | 0.260 | -1.675 | -1.452 | -0.550 | 0.306 | 1.509 | |
| 42502 | hsa-miR-204-3p | 0.108 | 0.124 | 5.607 | 2.82E-01 | 5.11E-01 | 0.427 | 0.076 | 0.152 | -0.036 | 0.266 | 0.181 | 0.241 | 0.062 | 0.138 | -0.207 | 0.000 | 0.187 | |
| 11005 | hsa-miR-204-5p | 0.069 | 0.366 | 5.727 | 4.71E-01 | 6.87E-01 | 0.590 | 0.462 | 0.273 | 0.341 | 0.443 | 0.295 | 0.143 | 0.480 | 0.351 | 0.209 | 0.419 | 0.391 | |
| 46917 | hsa-miR-205-5p | 0.184 | 1.000 | 5.856 | 1.43E-01 | 3.83E-01 | 1.073 | 1.555 | 1.442 | 0.891 | 0.815 | 0.776 | 0.915 | 0.902 | 1.373 | 0.774 | 0.696 | 0.787 | |
| 145845 | hsa-miR-20a-5p | -0.172 | -0.064 | 9.196 | 4.65E-01 | 6.82E-01 | -1.101 | 0.196 | 0.417 | -0.250 | 0.538 | -0.701 | -0.628 | -0.178 | 0.395 | -0.168 | 0.229 | 0.478 | |
| 42640 | hsa-miR-20b-5p | -0.004 | -0.124 | 5.786 | 9.78E-01 | 9.95E-01 | -0.636 | -0.020 | 0.516 | -0.218 | 0.130 | -0.529 | -0.621 | -0.352 | 0.189 | -0.297 | 0.087 | 0.263 | |
| 147506 | hsa-miR-21-5p | 0.406 | -0.063 | 8.786 | 2.31E-02 | 1.85E-01 | -0.484 | 0.839 | 0.860 | -0.609 | 0.366 | -0.132 | -0.027 | 0.312 | 0.105 | -0.919 | -0.605 | -0.462 | |
| 145852 | hsa-miR-210 | 1.973 | -0.966 | 7.133 | 2.04E-04 | 6.55E-02 | 0.877 | 0.067 | 1.290 | -1.477 | 0.563 | -1.196 | -2.712 | -1.828 | -1.820 | -1.472 | -1.809 | -2.076 | |
| 11011 | hsa-miR-211-5p | -0.377 | 1.048 | 5.974 | 3.48E-02 | 2.18E-01 | 0.706 | 0.616 | 0.897 | 1.099 | 0.966 | 0.874 | 1.403 | 1.756 | 1.325 | 1.023 | 0.995 | 0.917 | |
| 11020 | hsa-miR-22-3p | 0.410 | -0.182 | 7.657 | 6.70E-02 | 2.75E-01 | 0.534 | -0.612 | 0.197 | -0.168 | 0.276 | -0.091 | 0.115 | -0.526 | -0.282 | -0.240 | -0.510 | -0.879 | |
| 42532 | hsa-miR-22-5p | -0.071 | 0.388 | 5.702 | 5.85E-01 | 7.60E-01 | 0.264 | 0.167 | 0.410 | 0.616 | 0.344 | 0.317 | 0.564 | 0.686 | -0.013 | 0.647 | 0.444 | 0.217 | |
| 11022 | hsa-miR-221-3p | 0.140 | 0.233 | 7.412 | 2.51E-01 | 4.69E-01 | 0.001 | 0.490 | 0.733 | -0.310 | 0.447 | 0.457 | 0.480 | 0.273 | 0.352 | -0.126 | -0.033 | 0.034 | |
| 11023 | hsa-miR-222-3p | 0.472 | -0.411 | 8.560 | 2.05E-02 | 1.78E-01 | -0.092 | 0.397 | 0.121 | -1.360 | -0.024 | -0.090 | -0.059 | -0.105 | -0.331 | -1.205 | -1.135 | -1.048 | |
| 11024 | hsa-miR-223-3p | -0.294 | -0.560 | 5.741 | 1.56E-02 | 1.78E-01 | -1.100 | -0.701 | -0.773 | -0.459 | -0.616 | -0.592 | -0.521 | -0.557 | -0.319 | -0.107 | -0.402 | -0.570 | |
| 42744 | hsa-miR-23a-3p | 0.325 | -0.293 | 9.007 | 2.70E-02 | 1.97E-01 | -0.467 | -0.086 | -0.029 | -0.846 | 0.371 | 0.274 | 0.263 | -0.453 | -0.489 | -0.745 | -0.357 | -0.950 | |
| 169330 | hsa-miR-23b-3p | 0.020 | -0.162 | 7.482 | 8.99E-01 | 9.52E-01 | -1.655 | -0.446 | 0.270 | 0.051 | 0.482 | 0.386 | 0.676 | -0.596 | -0.116 | -0.249 | 0.168 | -0.917 | |
| 148217 | hsa-miR-23c | 0.152 | -0.596 | 5.427 | 2.16E-01 | 4.33E-01 | -1.062 | -0.989 | -0.207 | -0.460 | -0.016 | -0.385 | -0.145 | -1.097 | -0.772 | -0.627 | -0.267 | -1.123 | |
| 17506 | hsa-miR-24-3p | 0.081 | -0.188 | 8.389 | 5.22E-01 | 7.31E-01 | -1.280 | -0.345 | 0.172 | -0.266 | 0.384 | 0.449 | 0.427 | -0.395 | -0.075 | -0.361 | -0.097 | -0.869 | |
| 42682 | hsa-miR-25-3p | -0.087 | 0.243 | 6.632 | 4.88E-01 | 7.00E-01 | -0.421 | 0.891 | 0.644 | 0.041 | -0.131 | 0.173 | 0.226 | 0.433 | 0.813 | 0.128 | 0.211 | -0.090 | |
| 42523 | hsa-miR-26b-3p | -0.030 | 1.029 | 6.018 | 7.69E-01 | 8.70E-01 | 1.013 | 0.946 | 1.138 | 1.037 | 1.009 | 0.938 | 1.095 | 1.152 | 0.862 | 1.154 | 1.082 | 0.918 | |
| 146008 | hsa-miR-26b-5p | -0.001 | 0.362 | 7.415 | 9.96E-01 | 1.00E+00 | -0.781 | 0.190 | 1.055 | 0.457 | 0.800 | 0.450 | 0.285 | -0.031 | 0.380 | 0.434 | 0.799 | 0.309 | |
| 46483 | hsa-miR-27a-3p | 0.105 | -0.135 | 6.294 | 4.46E-01 | 6.66E-01 | -1.035 | 0.145 | 0.294 | -0.424 | 0.211 | 0.313 | 0.480 | -0.052 | -0.293 | -0.555 | -0.057 | -0.644 | |
| 147199 | hsa-miR-27b-3p | 0.026 | 0.046 | 7.182 | 8.44E-01 | 9.25E-01 | -1.288 | -0.359 | 0.359 | 0.601 | 0.328 | 0.711 | 0.881 | -0.592 | -0.006 | 0.329 | 0.374 | -0.790 | |
| 11038 | hsa-miR-299-5p | -0.044 | 0.513 | 5.740 | 6.35E-01 | 8.04E-01 | 0.482 | 0.640 | 0.541 | 0.179 | 0.685 | 0.422 | 0.345 | 0.655 | 0.609 | 0.366 | 0.676 | 0.562 | |
| 168687 | hsa-miR-29a-3p | 0.046 | -0.543 | 7.078 | 7.41E-01 | 8.55E-01 | -1.275 | -0.727 | -0.262 | 0.041 | 0.403 | -1.302 | -1.094 | -0.973 | -0.686 | -0.175 | 0.246 | -0.718 | |
| 145638 | hsa-miR-29a-5p | 0.349 | 0.114 | 5.812 | 1.44E-02 | 1.78E-01 | -0.120 | 0.353 | 0.357 | 0.924 | 0.373 | -0.155 | -0.382 | -0.295 | 0.112 | 0.183 | 0.292 | -0.270 | |
| 17810 | hsa-miR-29b-1-5p | 0.046 | 0.977 | 6.422 | 6.87E-01 | 8.28E-01 | 0.933 | 0.981 | 1.174 | 0.886 | 1.099 | 0.925 | 0.598 | 1.042 | 0.794 | 0.976 | 1.233 | 1.080 | |
| 11040 | hsa-miR-29b-3p | 0.207 | -0.049 | 6.192 | 9.96E-02 | 3.21E-01 | -0.468 | -0.086 | 0.446 | 0.442 | 0.426 | -0.431 | -0.591 | -0.118 | -0.130 | 0.010 | 0.260 | -0.344 | |
| 13143 | hsa-miR-301a-3p | -0.054 | -0.120 | 6.606 | 7.66E-01 | 8.69E-01 | -1.844 | 0.478 | 0.866 | -0.106 | -0.353 | 0.079 | 0.353 | 0.303 | 0.557 | -0.485 | -0.467 | -0.817 | |
| 27838 | hsa-miR-302d-3p | 0.161 | 0.786 | 5.825 | 3.34E-01 | 5.70E-01 | 1.330 | 0.582 | 1.262 | 0.352 | 0.716 | 0.955 | 0.736 | 0.714 | 0.421 | 0.679 | 0.890 | 0.793 | |
| 146112 | hsa-miR-30b-5p | 0.138 | -0.242 | 7.790 | 5.57E-01 | 7.46E-01 | -1.836 | -0.079 | 0.914 | -0.828 | 0.864 | -0.069 | -0.211 | -0.647 | 0.218 | -0.794 | 0.198 | -0.629 | |
| 42923 | hsa-miR-30c-5p | -0.068 | -0.268 | 7.165 | 6.91E-01 | 8.28E-01 | -1.525 | 0.010 | 0.340 | -0.751 | 0.113 | -0.001 | 0.138 | -0.133 | 0.015 | -0.870 | -0.057 | -0.498 | |
| 19596 | hsa-miR-30d-5p | 0.341 | 0.169 | 6.380 | 1.47E-02 | 1.78E-01 | 0.038 | 0.215 | 1.090 | -0.385 | 1.056 | 0.024 | -0.006 | -0.226 | 0.389 | -0.484 | 0.506 | -0.189 | |
| 28191 | hsa-miR-30e-5p | 0.237 | -0.264 | 5.724 | 5.75E-02 | 2.65E-01 | -0.386 | 0.221 | 0.175 | -0.585 | -0.134 | -0.166 | 0.052 | -0.255 | -0.151 | -0.922 | -0.402 | -0.620 | |
| 46320 | hsa-miR-31-3p | 0.102 | -0.441 | 5.737 | 3.36E-01 | 5.70E-01 | -1.291 | 0.520 | 1.634 | -0.913 | -1.591 | -0.700 | -0.493 | 0.446 | 1.306 | -1.095 | -1.634 | -1.484 | |
| 11052 | hsa-miR-31-5p | 0.004 | -0.731 | 6.640 | 9.70E-01 | 9.94E-01 | -2.337 | 0.687 | 1.201 | -1.223 | -2.231 | -0.469 | -0.397 | 0.271 | 1.462 | -1.341 | -2.053 | -2.340 | |
| 169380 | hsa-miR-3124-3p | 0.403 | -0.373 | 6.885 | 6.00E-02 | 2.67E-01 | 1.017 | -0.561 | -0.356 | -0.642 | -0.285 | -0.204 | 0.040 | -0.629 | -0.795 | -0.825 | -0.812 | -0.426 | |
| 146005 | hsa-miR-3129-5p | 0.176 | 0.720 | 5.649 | 2.19E-01 | 4.34E-01 | 1.172 | 0.859 | 1.283 | 0.127 | 0.771 | 0.637 | 0.614 | 0.551 | 0.665 | 0.515 | 0.695 | 0.755 | |
| 169232 | hsa-miR-3156-3p | -0.009 | -0.762 | 6.101 | 9.35E-01 | 9.71E-01 | -1.150 | -0.795 | -0.589 | -0.749 | -0.697 | -0.620 | -0.439 | -0.721 | -0.676 | -0.821 | -0.976 | -0.914 | |
| 146085 | hsa-miR-3170 | 0.039 | 0.039 | 5.648 | 6.68E-01 | 8.15E-01 | -0.120 | 0.019 | 0.129 | -0.038 | 0.248 | 0.109 | 0.054 | 0.039 | 0.097 | -0.097 | 0.125 | -0.102 | |
| 147891 | hsa-miR-3175 | 0.140 | -0.372 | 5.709 | 1.97E-01 | 4.11E-01 | -0.273 | 0.151 | -0.179 | -0.395 | -0.193 | -0.922 | -0.798 | -0.143 | -0.539 | -0.503 | -0.185 | -0.483 | |
| 147595 | hsa-miR-3178 | 0.265 | -0.804 | 5.863 | 1.52E-01 | 3.83E-01 | 0.319 | -1.664 | -0.789 | -1.775 | -0.678 | 0.558 | 0.818 | -1.746 | -1.756 | -1.486 | -1.137 | -0.312 | |
| 147667 | hsa-miR-3182 | 0.214 | -1.082 | 8.398 | 1.56E-01 | 3.83E-01 | -1.074 | -1.007 | -0.962 | -0.600 | -1.203 | -1.002 | -0.651 | -1.063 | -1.074 | -0.814 | -1.913 | -1.618 | |
| 29575 | hsa-miR-32-3p | 0.282 | 0.171 | 6.293 | 8.13E-02 | 2.97E-01 | 0.857 | 0.325 | 0.235 | 0.163 | 0.172 | 0.118 | 0.121 | 0.218 | 0.018 | 0.062 | -0.010 | -0.228 | |
| 11053 | hsa-miR-32-5p | 0.239 | 0.225 | 6.267 | 1.49E-01 | 3.83E-01 | -0.492 | 0.696 | 0.959 | 0.313 | 0.216 | 0.378 | 0.166 | 0.819 | -0.034 | -0.093 | 0.054 | -0.276 | |
| 27533 | hsa-miR-320a | 0.304 | -0.007 | 7.343 | 4.71E-02 | 2.45E-01 | 0.524 | -0.014 | -0.482 | 0.486 | 0.685 | -0.328 | -0.488 | 0.156 | -0.821 | 0.365 | 0.157 | -0.317 | |
| 46324 | hsa-miR-320b | 0.386 | -0.197 | 7.120 | 3.53E-02 | 2.18E-01 | 0.381 | -0.112 | -0.577 | 0.308 | 0.625 | -0.646 | -0.537 | -0.060 | -1.502 | 0.192 | 0.000 | -0.430 | |
| 169363 | hsa-miR-320c | 0.321 | -0.307 | 5.718 | 7.73E-02 | 2.88E-01 | 0.053 | -0.371 | -0.289 | 0.045 | 0.388 | -0.706 | -0.450 | -0.279 | -1.273 | -0.075 | -0.179 | -0.547 | |
| 11058 | hsa-miR-325 | -0.032 | 0.705 | 5.732 | 7.86E-01 | 8.85E-01 | 0.810 | 0.564 | 0.869 | 0.411 | 0.863 | 0.615 | 0.815 | 0.934 | 0.594 | 0.648 | 0.686 | 0.650 | |
| 42887 | hsa-miR-331-3p | 0.062 | -0.170 | 5.974 | 6.87E-01 | 8.28E-01 | -1.206 | 0.263 | 0.228 | 0.331 | -0.101 | -0.351 | -0.033 | 0.176 | -0.005 | -0.276 | -0.394 | -0.675 | |
| 145745 | hsa-miR-335-3p | 0.153 | -0.216 | 7.539 | 1.89E-01 | 4.07E-01 | -0.203 | -0.500 | 0.171 | -0.222 | 0.129 | -0.212 | -0.016 | -0.564 | -0.035 | -0.288 | -0.325 | -0.529 | |
| 11065 | hsa-miR-335-5p | -0.075 | 0.170 | 5.970 | 5.27E-01 | 7.31E-01 | -0.562 | 0.044 | 0.754 | -0.172 | 0.347 | 0.385 | 0.728 | 0.015 | 0.574 | -0.242 | 0.280 | -0.109 | |
| 42592 | hsa-miR-338-3p | 0.064 | 0.407 | 5.959 | 7.02E-01 | 8.38E-01 | 0.393 | -0.206 | 1.532 | 0.144 | 0.466 | 0.306 | 0.139 | 0.327 | 0.653 | 0.242 | 0.309 | 0.581 | |
| 42739 | hsa-miR-339-5p | -0.006 | -0.647 | 5.776 | 9.63E-01 | 9.94E-01 | -1.297 | -0.510 | 0.212 | -0.761 | -0.695 | -0.853 | -0.354 | -0.585 | -0.142 | -1.041 | -0.843 | -0.899 | |
| 145859 | hsa-miR-33a-5p | 0.365 | 0.075 | 6.386 | 3.27E-02 | 2.17E-01 | -0.361 | 0.386 | 1.019 | 0.495 | 0.081 | -0.073 | -0.206 | 0.457 | 0.036 | 0.018 | -0.567 | -0.382 | |
| 168586 | hsa-miR-34a-5p | 0.096 | 0.008 | 6.117 | 5.33E-01 | 7.31E-01 | -0.977 | 0.741 | 0.728 | -0.031 | -0.429 | 0.300 | 0.667 | 0.271 | 0.208 | -0.076 | -0.756 | -0.555 | |
| 11074 | hsa-miR-34c-5p | 0.060 | 0.734 | 5.685 | 6.41E-01 | 8.04E-01 | 1.126 | 0.622 | 1.248 | 0.264 | 0.714 | 0.609 | 0.555 | 0.659 | 0.915 | 0.688 | 0.742 | 0.663 | |
| 148420 | hsa-miR-3607-3p | 0.196 | -0.175 | 7.321 | 7.65E-02 | 2.88E-01 | -0.162 | 0.018 | 0.153 | -0.100 | -0.102 | -0.269 | -0.254 | -0.233 | -0.295 | -0.129 | -0.332 | -0.391 | |
| 14301 | hsa-miR-361-5p | -0.126 | 0.682 | 6.779 | 2.31E-01 | 4.49E-01 | 0.504 | 0.772 | 0.516 | 0.430 | 1.034 | 0.460 | 0.676 | 0.653 | 0.881 | 0.623 | 1.059 | 0.583 | |
| 148493 | hsa-miR-3613-3p | 0.392 | -1.214 | 9.035 | 5.25E-02 | 2.51E-01 | -0.363 | -1.091 | -1.083 | -1.355 | -1.202 | -1.016 | -1.557 | -0.794 | -1.239 | -1.539 | -1.668 | -1.665 | |
| 148413 | hsa-miR-3614-3p | 0.277 | 0.768 | 5.756 | 3.72E-02 | 2.21E-01 | 1.255 | 1.082 | 0.924 | 0.713 | 0.870 | 0.595 | 0.615 | 0.961 | 0.494 | 0.457 | 0.710 | 0.537 | |
| 148317 | hsa-miR-3621 | 0.186 | -0.395 | 5.881 | 2.17E-01 | 4.33E-01 | 0.382 | -0.920 | -0.397 | -0.720 | -0.296 | 0.142 | 0.319 | -0.746 | -1.226 | -0.745 | -0.552 | 0.027 | |
| 148481 | hsa-miR-3646 | 0.304 | -0.303 | 7.689 | 7.53E-02 | 2.88E-01 | 0.179 | -0.441 | -0.210 | -0.242 | -0.159 | -0.032 | 0.273 | -0.483 | -0.461 | -0.499 | -0.836 | -0.721 | |
| 148327 | hsa-miR-3651 | 0.035 | -0.064 | 6.231 | 8.05E-01 | 8.91E-01 | -0.552 | 0.036 | 0.441 | -0.136 | -0.008 | -0.060 | 0.189 | -0.183 | -0.111 | -0.159 | -0.154 | -0.067 | |
| 148377 | hsa-miR-3653 | 0.233 | -0.357 | 5.813 | 5.79E-02 | 2.65E-01 | 0.090 | -0.563 | 0.501 | -0.441 | -0.029 | -1.001 | -0.925 | -0.642 | 0.277 | -0.644 | -0.429 | -0.481 | |
| 11078 | hsa-miR-365a-3p/b-3p | 0.142 | 0.628 | 6.608 | 3.51E-01 | 5.74E-01 | 0.186 | 0.438 | 0.838 | 0.542 | 1.189 | 1.002 | 1.160 | 0.401 | 0.498 | 0.398 | 0.373 | 0.509 | |
| 148214 | hsa-miR-3675-3p | -0.150 | -0.752 | 5.882 | 1.67E-01 | 3.90E-01 | -1.081 | -1.012 | -0.783 | -0.581 | -0.815 | -0.690 | -0.854 | -0.752 | -0.547 | -0.487 | -0.686 | -0.735 | |
| 148410 | hsa-miR-3676-3p | -0.148 | 0.190 | 7.184 | 1.95E-01 | 4.10E-01 | -0.197 | -0.234 | 0.053 | 0.249 | 0.313 | 0.511 | 0.634 | 0.186 | -0.036 | 0.389 | 0.230 | 0.181 | |
| 168763 | hsa-miR-3676-5p | 0.071 | -0.625 | 5.522 | 5.95E-01 | 7.67E-01 | -1.605 | -0.412 | 0.066 | -0.483 | -0.787 | -0.317 | -0.278 | -0.331 | -0.474 | -0.560 | -1.127 | -1.194 | |
| 148038 | hsa-miR-3679-3p | -0.224 | 1.123 | 7.461 | 6.71E-02 | 2.75E-01 | 0.955 | 0.797 | 1.010 | 1.274 | 1.093 | 0.934 | 1.144 | 1.447 | 1.271 | 1.263 | 1.161 | 1.123 | |
| 148156 | hsa-miR-3686 | 0.327 | -0.539 | 6.370 | 5.02E-02 | 2.48E-01 | 0.592 | -0.607 | -0.549 | -0.693 | -0.290 | -0.706 | -0.675 | -0.712 | -0.782 | -0.868 | -0.656 | -0.522 | |
| 148282 | hsa-miR-3714 | 0.156 | 0.461 | 5.706 | 1.59E-01 | 3.83E-01 | 0.732 | 0.611 | 0.551 | 0.374 | 0.673 | 0.293 | 0.318 | 0.475 | 0.515 | 0.348 | 0.380 | 0.261 | |
| 168978 | hsa-miR-371b-5p | 0.578 | -1.188 | 7.590 | 2.31E-02 | 1.85E-01 | 1.099 | -2.459 | -1.395 | -2.279 | -0.378 | 0.019 | 0.140 | -2.533 | -1.984 | -2.436 | -1.590 | -0.460 | |
| 145844 | hsa-miR-374a-5p | -0.120 | 0.053 | 5.857 | 4.63E-01 | 6.82E-01 | -0.614 | 0.129 | 0.645 | -0.162 | 0.022 | -0.059 | 0.181 | 0.304 | 0.268 | -0.392 | 0.029 | 0.290 | |
| 42476 | hsa-miR-374b-3p | -0.014 | 0.413 | 5.636 | 8.99E-01 | 9.52E-01 | 0.052 | 0.709 | 0.228 | 0.386 | 0.691 | 0.371 | 0.410 | 0.543 | 0.412 | 0.289 | 0.498 | 0.367 | |
| 148098 | hsa-miR-374b-5p | -0.143 | -0.203 | 6.430 | 4.73E-01 | 6.87E-01 | -0.905 | -0.480 | 0.326 | -0.644 | 0.241 | -0.183 | 0.183 | -0.424 | 0.209 | -1.114 | 0.060 | 0.299 | |
| 148430 | hsa-miR-374c-5p | -0.047 | -0.126 | 6.283 | 7.36E-01 | 8.54E-01 | -0.468 | -0.266 | 0.486 | -0.574 | 0.093 | -0.169 | 0.107 | -0.478 | 0.206 | -0.675 | 0.038 | 0.184 | |
| 46918 | hsa-miR-375 | -0.054 | 0.456 | 6.056 | 6.01E-01 | 7.72E-01 | 0.450 | 0.119 | 1.245 | 0.265 | 0.329 | 0.165 | 0.015 | 0.138 | 1.150 | 0.615 | 0.426 | 0.555 | |
| 42899 | hsa-miR-377-5p | -0.160 | 0.555 | 5.675 | 1.77E-01 | 3.98E-01 | 0.522 | 0.469 | 0.562 | 0.285 | 0.565 | 0.447 | 0.864 | 0.873 | 0.574 | 0.565 | 0.440 | 0.494 | |
| 148668 | hsa-miR-378a-3p | -0.079 | -0.017 | 6.441 | 4.84E-01 | 6.99E-01 | 0.345 | -0.223 | 0.189 | -0.143 | -0.259 | -0.249 | 0.136 | -0.122 | 0.046 | -0.363 | -0.001 | 0.442 | |
| 147755 | hsa-miR-378c | -0.185 | 0.666 | 6.284 | 8.13E-02 | 2.97E-01 | 0.491 | 0.500 | 0.653 | 0.671 | 0.508 | 0.619 | 0.792 | 0.638 | 0.658 | 0.794 | 0.757 | 0.910 | |
| 168637 | hsa-miR-3940-5p | 0.644 | -1.460 | 7.123 | 1.90E-02 | 1.78E-01 | 0.509 | -2.621 | -1.380 | -2.714 | -1.129 | 0.511 | 0.551 | -2.818 | -2.733 | -2.709 | -1.804 | -1.175 | |
| 169024 | hsa-miR-3960 | 0.605 | -1.307 | 7.978 | 2.67E-02 | 1.97E-01 | 1.181 | -2.545 | -1.433 | -2.688 | -0.530 | -0.011 | 0.176 | -2.760 | -2.106 | -2.688 | -1.753 | -0.525 | |
| 42730 | hsa-miR-423-3p | 0.000 | -0.269 | 6.328 | 1.00E+00 | 1.00E+00 | -0.852 | 0.033 | 0.059 | -0.274 | -0.366 | -0.214 | 0.019 | 0.009 | -0.348 | -0.399 | -0.419 | -0.475 | |
| 27565 | hsa-miR-423-5p | 0.084 | -0.096 | 6.696 | 4.22E-01 | 6.45E-01 | 0.089 | -0.312 | 0.077 | -0.218 | -0.034 | 0.073 | -0.021 | -0.135 | -0.218 | -0.180 | -0.103 | -0.174 | |
| 17608 | hsa-miR-425-5p | 0.073 | -0.150 | 5.843 | 5.02E-01 | 7.13E-01 | 0.061 | -0.365 | -0.068 | 0.518 | -0.201 | -0.627 | -0.477 | -0.479 | -0.197 | 0.120 | -0.306 | 0.218 | |
| 147631 | hsa-miR-4258 | 0.143 | 0.361 | 5.898 | 1.90E-01 | 4.07E-01 | 0.543 | 0.288 | 0.583 | 0.339 | 0.540 | 0.301 | 0.485 | 0.247 | 0.310 | 0.164 | 0.225 | 0.304 | |
| 147751 | hsa-miR-4274 | 0.018 | 0.905 | 6.348 | 8.53E-01 | 9.28E-01 | 0.979 | 0.835 | 1.035 | 0.896 | 1.002 | 0.737 | 0.693 | 0.924 | 0.772 | 1.071 | 0.958 | 0.956 | |
| 147767 | hsa-miR-4279 | 0.164 | 0.338 | 7.081 | 3.41E-01 | 5.73E-01 | 1.473 | -0.029 | 0.317 | 0.136 | 0.350 | 0.274 | 0.581 | 0.155 | 0.080 | 0.199 | 0.076 | 0.446 | |
| 169129 | hsa-miR-4284 | -0.515 | -1.085 | 11.711 | 2.14E-02 | 1.81E-01 | -1.540 | -0.257 | -1.589 | -2.261 | -0.648 | -1.763 | -1.917 | -0.170 | -0.118 | -1.702 | -0.312 | -0.745 | |
| 169409 | hsa-miR-4286 | 0.505 | -0.605 | 9.763 | 2.00E-02 | 1.78E-01 | -0.358 | -0.738 | -0.097 | -0.586 | 0.352 | -0.687 | -0.448 | -1.078 | -0.715 | -0.670 | -0.722 | -1.510 | |
| 147588 | hsa-miR-4288 | 0.401 | -0.283 | 8.555 | 3.51E-02 | 2.18E-01 | 0.207 | 0.126 | 0.247 | -1.093 | 0.004 | 0.012 | 0.040 | -0.255 | 0.054 | -0.995 | -0.918 | -0.829 | |
| 147735 | hsa-miR-4289 | 0.061 | -0.100 | 5.744 | 6.38E-01 | 8.04E-01 | -0.506 | -0.050 | 0.476 | 0.012 | -0.054 | -0.293 | -0.092 | -0.462 | 0.069 | -0.007 | -0.160 | -0.131 | |
| 13171 | hsa-miR-429 | -0.068 | -0.081 | 7.304 | 6.41E-01 | 8.04E-01 | -0.689 | -1.842 | 1.444 | -0.031 | 0.614 | -0.182 | -0.091 | -1.721 | 0.986 | -0.108 | 0.614 | 0.041 | |
| 169282 | hsa-miR-4290 | 0.389 | 0.070 | 6.649 | 4.03E-02 | 2.27E-01 | 1.376 | -0.107 | -0.039 | 0.235 | 0.093 | 0.030 | 0.314 | -0.272 | -0.422 | -0.208 | -0.335 | 0.179 | |
| 147616 | hsa-miR-4291 | 0.181 | -0.386 | 5.941 | 9.25E-02 | 3.16E-01 | -0.697 | -0.453 | 0.115 | -0.554 | -0.188 | 0.003 | 0.003 | -0.749 | -0.126 | -0.554 | -0.355 | -1.079 | |
| 169407 | hsa-miR-4301 | 0.056 | -0.458 | 5.930 | 5.60E-01 | 7.46E-01 | -0.614 | -0.516 | -0.053 | -0.731 | -0.136 | -0.531 | -0.400 | -0.512 | -0.218 | -0.894 | -0.245 | -0.646 | |
| 147722 | hsa-miR-4306 | 0.065 | -0.232 | 5.880 | 5.76E-01 | 7.52E-01 | -0.107 | -0.467 | 0.680 | -0.403 | -0.548 | -0.352 | 0.016 | -0.441 | 0.305 | -0.520 | -0.675 | -0.271 | |
| 42674 | hsa-miR-431-3p | -0.085 | 0.601 | 5.953 | 5.57E-01 | 7.46E-01 | 0.585 | 0.305 | 0.731 | 0.748 | 0.455 | 0.527 | 0.780 | 0.778 | 0.102 | 0.786 | 0.731 | 0.686 | |
| 145705 | hsa-miR-431-5p | 0.472 | 0.690 | 5.807 | 8.21E-03 | 1.78E-01 | 1.462 | 1.072 | 1.267 | 0.475 | 0.707 | 0.572 | 0.391 | 0.660 | 0.328 | 0.364 | 0.504 | 0.480 | |
| 147907 | hsa-miR-4312 | -0.040 | 0.180 | 5.804 | 7.11E-01 | 8.45E-01 | 0.037 | 0.143 | 0.011 | 0.325 | 0.353 | 0.092 | 0.118 | 0.254 | 0.371 | 0.142 | 0.143 | 0.175 | |
| 168709 | hsa-miR-4429 | 0.412 | -0.564 | 5.626 | 1.44E-02 | 1.78E-01 | -0.421 | -0.451 | -0.709 | 0.275 | 0.242 | -1.082 | -0.768 | -0.823 | -1.349 | -0.223 | -0.466 | -0.988 | |
| 169171 | hsa-miR-4436b-5p | 0.047 | 0.373 | 5.713 | 6.59E-01 | 8.15E-01 | 0.557 | 0.241 | 0.562 | 0.248 | 0.435 | 0.336 | 0.435 | 0.414 | 0.288 | 0.335 | 0.317 | 0.312 | |
| 169188 | hsa-miR-4443 | 0.298 | -0.628 | 10.159 | 1.25E-01 | 3.67E-01 | -0.279 | -0.708 | -0.293 | -1.050 | -0.083 | -0.462 | -0.392 | -0.806 | -0.347 | -0.821 | -1.127 | -1.166 | |
| 169015 | hsa-miR-4454 | 0.185 | -0.625 | 14.716 | 3.83E-01 | 6.02E-01 | -0.409 | -0.572 | -0.057 | -1.559 | -0.213 | -0.381 | -0.338 | -1.174 | -0.197 | -0.642 | -0.674 | -1.280 | |
| 168919 | hsa-miR-4456 | -0.053 | -0.891 | 7.356 | 6.38E-01 | 8.04E-01 | -1.498 | -0.059 | -1.501 | -1.053 | -1.367 | -0.026 | 0.157 | -0.022 | -1.215 | -1.176 | -1.672 | -1.258 | |
| 169285 | hsa-miR-4467 | 0.517 | -0.933 | 7.019 | 1.64E-02 | 1.78E-01 | 1.036 | -1.741 | -1.170 | -1.740 | -0.455 | 0.023 | 0.158 | -1.902 | -1.679 | -2.064 | -1.323 | -0.341 | |
| 169395 | hsa-miR-4484 | 0.017 | -1.285 | 5.050 | 9.16E-01 | 9.55E-01 | 0.184 | -1.552 | -1.742 | -1.734 | -0.753 | -2.060 | -1.726 | -0.881 | -2.048 | -1.820 | -0.865 | -0.418 | |
| 169110 | hsa-miR-4497 | 0.212 | -0.712 | 5.747 | 1.46E-01 | 3.83E-01 | 0.117 | -1.273 | -1.011 | -1.436 | -0.720 | 0.686 | 1.037 | -1.483 | -1.480 | -1.398 | -1.094 | -0.491 | |
| 169385 | hsa-miR-4500 | 0.090 | -0.298 | 5.809 | 5.40E-01 | 7.31E-01 | -0.745 | -0.103 | 0.403 | -0.389 | -0.151 | -0.529 | -0.522 | -0.328 | -0.342 | -0.283 | -0.251 | -0.328 | |
| 42892 | hsa-miR-450b-3p | -0.154 | 0.914 | 5.890 | 1.91E-01 | 4.07E-01 | 1.009 | 0.802 | 0.817 | 0.855 | 0.773 | 0.768 | 1.183 | 1.163 | 1.019 | 0.887 | 0.903 | 0.789 | |
| 29379 | hsa-miR-452-5p | -0.195 | 0.139 | 6.037 | 1.47E-01 | 3.83E-01 | -0.365 | -0.597 | 0.794 | -0.817 | 0.290 | 0.944 | 1.421 | -0.464 | 0.679 | -0.163 | 0.232 | -0.287 | |
| 169159 | hsa-miR-4521 | -0.177 | -0.538 | 5.777 | 2.43E-01 | 4.64E-01 | -1.517 | -0.378 | -0.620 | 0.040 | -0.517 | -0.772 | -0.509 | -0.169 | -0.108 | -0.348 | -0.684 | -0.882 | |
| 168844 | hsa-miR-4532 | 0.235 | -1.390 | 7.230 | 1.50E-01 | 3.83E-01 | -0.666 | -1.564 | -1.297 | -1.647 | -1.069 | -1.389 | -1.097 | -1.479 | -1.394 | -1.880 | -1.710 | -1.483 | |
| 148620 | hsa-miR-454-3p | -0.124 | 0.164 | 5.541 | 2.96E-01 | 5.31E-01 | -0.352 | -0.041 | 0.550 | 0.041 | 0.093 | 0.321 | 0.486 | 0.317 | 0.313 | 0.146 | 0.031 | 0.063 | |
| 169102 | hsa-miR-4639-3p | -0.013 | -0.557 | 6.377 | 9.04E-01 | 9.54E-01 | 0.090 | -0.792 | -0.807 | -0.595 | -0.638 | -0.639 | -0.406 | -0.560 | -0.818 | -0.676 | -0.594 | -0.248 | |
| 46731 | hsa-miR-4657 | 0.069 | -0.397 | 7.418 | 5.58E-01 | 7.46E-01 | 0.026 | 0.067 | -0.371 | -0.533 | -0.068 | -1.295 | -1.077 | 0.139 | -0.337 | -0.503 | -0.484 | -0.323 | |
| 168798 | hsa-miR-4668-5p | 0.455 | -1.182 | 7.178 | 2.85E-02 | 2.03E-01 | -0.366 | -0.933 | -0.980 | -0.984 | -1.273 | -1.189 | -1.577 | -0.462 | -1.518 | -1.746 | -1.662 | -1.489 | |
| 169070 | hsa-miR-4695-3p | 0.308 | -0.710 | 8.788 | 5.89E-02 | 2.66E-01 | -0.541 | -0.783 | -0.604 | -0.566 | -0.192 | -0.651 | -0.514 | -0.977 | -0.806 | -0.567 | -1.009 | -1.312 | |
| 169028 | hsa-miR-4708-3p | 0.710 | -1.401 | 8.159 | 8.91E-03 | 1.78E-01 | 1.145 | -2.650 | -1.547 | -2.732 | -0.602 | 0.109 | 0.180 | -2.987 | -2.286 | -2.970 | -1.866 | -0.609 | |
| 169311 | hsa-miR-4714-5p | 0.441 | -0.593 | 6.317 | 1.02E-02 | 1.78E-01 | 0.517 | -0.643 | -0.127 | -0.685 | -0.508 | -0.792 | -0.787 | -0.818 | -0.872 | -1.129 | -0.790 | -0.487 | |
| 169323 | hsa-miR-4723-3p | -0.167 | 0.923 | 6.880 | 1.49E-01 | 3.83E-01 | 0.946 | 0.719 | 0.716 | 1.084 | 0.836 | 0.733 | 0.851 | 1.214 | 1.081 | 1.106 | 0.909 | 0.875 | |
| 169031 | hsa-miR-4726-5p | 0.274 | -1.136 | 5.927 | 9.47E-02 | 3.18E-01 | -0.011 | -1.409 | -1.031 | -1.422 | -0.912 | -1.211 | -1.050 | -1.318 | -1.262 | -1.588 | -1.478 | -0.943 | |
| 169182 | hsa-miR-4728-3p | 0.161 | -0.121 | 5.829 | 1.74E-01 | 3.94E-01 | 0.170 | 0.017 | 0.080 | -0.124 | -0.239 | -0.148 | 0.098 | -0.217 | -0.240 | -0.386 | -0.253 | -0.211 | |
| 169239 | hsa-miR-4732-5p | 0.467 | -0.704 | 5.647 | 1.44E-02 | 1.78E-01 | 1.233 | -1.222 | -0.856 | -0.961 | 0.164 | -1.182 | -1.046 | -1.345 | -1.800 | -1.235 | -0.464 | 0.266 | |
| 169130 | hsa-miR-4764-3p | 0.028 | -0.654 | 6.365 | 7.92E-01 | 8.85E-01 | -0.075 | -0.870 | -0.487 | -1.031 | -0.571 | -0.806 | -0.924 | -0.649 | -0.770 | -1.211 | -0.523 | 0.068 | |
| 168943 | hsa-miR-4769-3p | -0.212 | 0.136 | 5.795 | 8.69E-02 | 3.07E-01 | 0.029 | -0.264 | 0.219 | 0.184 | 0.001 | 0.012 | 0.358 | 0.369 | 0.188 | 0.218 | 0.158 | 0.159 | |
| 168915 | hsa-miR-4780 | 0.105 | -0.252 | 7.097 | 3.33E-01 | 5.70E-01 | -0.384 | 0.042 | 0.163 | -0.195 | 0.018 | -0.845 | -0.787 | -0.201 | -0.202 | -0.262 | -0.148 | -0.227 | |
| 169050 | hsa-miR-4787-5p | 0.703 | -1.241 | 8.201 | 1.48E-02 | 1.78E-01 | 1.257 | -2.429 | -1.369 | -2.375 | -0.359 | -0.059 | 0.162 | -2.678 | -2.166 | -2.609 | -1.742 | -0.523 | |
| 168995 | hsa-miR-4791 | -0.306 | -0.277 | 5.971 | 9.64E-02 | 3.18E-01 | -1.336 | -0.067 | -0.015 | -0.638 | -0.245 | -0.277 | 0.021 | -0.237 | -0.071 | -0.290 | -0.043 | -0.122 | |
| 169022 | hsa-miR-4797-5p | -0.006 | -1.002 | 5.963 | 9.60E-01 | 9.94E-01 | -1.643 | -1.623 | -0.604 | -0.832 | -0.546 | -0.785 | -0.517 | -1.235 | -0.952 | -0.933 | -0.815 | -1.543 | |
| 169313 | hsa-miR-4800-3p | 0.448 | -0.701 | 7.992 | 5.12E-02 | 2.49E-01 | 1.182 | -0.918 | -0.749 | -1.165 | -0.368 | -0.848 | -0.424 | -1.280 | -1.036 | -1.315 | -1.328 | -0.168 | |
| 148682 | hsa-miR-483-3p | 0.364 | 0.149 | 6.633 | 1.16E-01 | 3.46E-01 | 1.885 | 0.108 | -0.016 | 0.083 | -0.078 | 0.004 | -0.050 | -0.001 | -0.195 | 0.003 | -0.128 | 0.171 | |
| 147701 | hsa-miR-491-3p | 0.238 | -0.839 | 9.717 | 1.55E-01 | 3.83E-01 | -0.632 | -1.548 | -0.585 | -0.558 | -0.239 | -0.761 | -0.659 | -1.630 | -0.790 | -0.285 | -1.112 | -1.273 | |
| 148059 | hsa-miR-493-5p | -0.134 | -0.281 | 6.507 | 2.35E-01 | 4.55E-01 | -0.461 | -0.286 | -0.472 | -0.317 | -0.280 | -0.274 | -0.180 | -0.019 | -0.447 | -0.316 | -0.363 | 0.040 | |
| 42442 | hsa-miR-498 | 0.218 | 0.358 | 5.645 | 1.54E-01 | 3.83E-01 | 0.945 | 0.584 | 0.731 | -0.071 | 0.291 | 0.326 | 0.417 | 0.316 | 0.103 | 0.134 | 0.298 | 0.226 | |
| 14313 | hsa-miR-499a-5p | 0.360 | 0.826 | 5.801 | 1.59E-02 | 1.78E-01 | 1.158 | 1.361 | 0.994 | 0.788 | 1.178 | 0.557 | 0.525 | 0.520 | 0.895 | 0.367 | 0.961 | 0.606 | |
| 11134 | hsa-miR-502-5p | 0.453 | 0.594 | 5.824 | 2.95E-03 | 1.78E-01 | 1.321 | 0.920 | 0.919 | 0.559 | 0.658 | 0.547 | 0.382 | 0.514 | 0.381 | 0.087 | 0.430 | 0.412 | |
| 42490 | hsa-miR-505-5p | 0.071 | 0.538 | 5.828 | 4.61E-01 | 6.82E-01 | 0.567 | 0.586 | 0.573 | 0.447 | 0.712 | 0.557 | 0.585 | 0.630 | 0.287 | 0.409 | 0.574 | 0.528 | |
| 11140 | hsa-miR-508-3p | -0.146 | 0.722 | 5.987 | 1.71E-01 | 3.90E-01 | 0.627 | 0.518 | 0.936 | 0.593 | 0.729 | 0.492 | 0.741 | 0.846 | 0.777 | 0.778 | 0.814 | 0.817 | |
| 11141 | hsa-miR-509-3p | -0.030 | 0.723 | 5.875 | 8.01E-01 | 8.90E-01 | 0.713 | 0.590 | 0.889 | 0.618 | 0.715 | 0.720 | 0.733 | 0.947 | 0.559 | 0.934 | 0.704 | 0.548 | |
| 168878 | hsa-miR-5100 | 0.321 | -0.465 | 14.448 | 2.10E-01 | 4.29E-01 | -0.133 | -0.469 | 0.001 | -0.659 | 0.015 | -0.584 | -0.562 | -1.155 | -0.248 | 0.330 | -0.859 | -1.262 | |
| 145690 | hsa-miR-512-5p | 0.493 | 0.684 | 5.602 | 1.20E-02 | 1.78E-01 | 1.583 | 1.463 | 0.748 | 0.726 | 0.854 | 0.212 | 0.341 | 0.745 | 0.688 | 0.094 | 0.330 | 0.429 | |
| 42581 | hsa-miR-513a-5p | 0.240 | 0.205 | 5.890 | 1.29E-01 | 3.73E-01 | 0.986 | 0.134 | 0.331 | 0.172 | 0.205 | 0.119 | 0.067 | 0.214 | -0.365 | 0.277 | 0.121 | 0.195 | |
| 145717 | hsa-miR-516a-3p/b-3p | -0.454 | 0.793 | 5.723 | 1.67E-03 | 1.78E-01 | 0.389 | 0.367 | 0.332 | 0.800 | 0.759 | 0.747 | 1.572 | 0.711 | 0.805 | 1.006 | 1.112 | 0.910 | |
| 46221 | hsa-miR-519d | 0.090 | 0.658 | 5.887 | 3.53E-01 | 5.75E-01 | 0.818 | 0.671 | 0.732 | 0.582 | 0.765 | 0.653 | 0.498 | 0.734 | 0.693 | 0.585 | 0.616 | 0.552 | |
| 46744 | hsa-miR-526b-5p | 0.177 | 0.850 | 5.809 | 1.52E-01 | 3.83E-01 | 1.169 | 1.117 | 1.167 | 0.430 | 1.128 | 0.620 | 0.772 | 0.629 | 0.934 | 0.534 | 0.911 | 0.789 | |
| 14271 | hsa-miR-539-5p | 0.279 | 0.616 | 5.667 | 1.01E-01 | 3.21E-01 | 1.073 | 1.158 | 1.110 | 0.247 | 0.556 | 0.388 | 0.732 | 0.504 | 0.503 | 0.290 | 0.468 | 0.364 | |
| 168951 | hsa-miR-548as-3p | -0.390 | -1.249 | 5.416 | 2.69E-02 | 1.97E-01 | -1.207 | -1.466 | -1.619 | -1.476 | -1.372 | -1.524 | -1.099 | -0.757 | -1.279 | -1.801 | -1.085 | -0.303 | |
| 46705 | hsa-miR-548k | -0.118 | 0.487 | 5.825 | 2.40E-01 | 4.61E-01 | 0.412 | 0.378 | 0.619 | 0.353 | 0.432 | 0.374 | 0.407 | 0.609 | 0.551 | 0.650 | 0.522 | 0.536 | |
| 168933 | hsa-miR-5581-3p | 0.192 | -0.850 | 7.806 | 1.62E-01 | 3.89E-01 | -0.632 | -1.337 | -0.218 | -1.075 | -0.265 | -0.997 | -0.762 | -1.296 | -0.418 | -1.221 | -0.933 | -1.042 | |
| 169169 | hsa-miR-5684 | 0.219 | -0.716 | 7.743 | 7.56E-02 | 2.88E-01 | -0.593 | -0.703 | -1.011 | -0.651 | -0.318 | -0.364 | -0.208 | -0.985 | -1.152 | -0.870 | -0.897 | -0.840 | |
| 169376 | hsa-miR-5701 | 0.427 | -0.335 | 8.260 | 2.00E-02 | 1.78E-01 | 0.724 | 0.152 | -0.291 | 0.034 | -0.420 | -0.926 | -0.836 | -0.263 | -0.696 | -0.006 | -1.086 | -0.400 | |
| 169211 | hsa-miR-5704 | -0.186 | -0.483 | 6.632 | 1.37E-01 | 3.83E-01 | -0.314 | -0.523 | -0.736 | -0.469 | -0.897 | -0.518 | -0.492 | -0.276 | -0.429 | -0.681 | -0.333 | -0.128 | |
| 17490 | hsa-miR-571 | 0.402 | 0.468 | 5.631 | 1.03E-02 | 1.78E-01 | 1.150 | 0.798 | 0.666 | 0.346 | 0.582 | 0.474 | 0.433 | 0.391 | 0.132 | 0.151 | 0.306 | 0.192 | |
| 27740 | hsa-miR-574-5p | -0.029 | 0.512 | 6.188 | 7.89E-01 | 8.85E-01 | 0.529 | 0.235 | 0.748 | 0.588 | 0.342 | 0.540 | 0.635 | 0.650 | 0.652 | 0.512 | 0.389 | 0.323 | |
| 17302 | hsa-miR-578 | 0.277 | 0.984 | 5.855 | 8.69E-02 | 3.07E-01 | 1.459 | 1.471 | 1.043 | 1.020 | 0.981 | 0.758 | 0.777 | 0.701 | 1.329 | 0.663 | 0.801 | 0.801 | |
| 14962 | hsa-miR-581 | 0.171 | 0.506 | 5.819 | 2.13E-01 | 4.29E-01 | 1.189 | 0.388 | 0.467 | 0.242 | 0.745 | 0.520 | 0.461 | 0.512 | 0.312 | 0.344 | 0.472 | 0.422 | |
| 145647 | hsa-miR-584-5p | 0.039 | 0.203 | 6.054 | 7.34E-01 | 8.54E-01 | 0.015 | 0.113 | 0.549 | -0.356 | 0.752 | 0.263 | 0.324 | 0.312 | 0.266 | -0.206 | 0.358 | 0.049 | |
| 42567 | hsa-miR-590-3p | -0.203 | 0.737 | 5.662 | 1.57E-01 | 3.83E-01 | 1.051 | 0.469 | 0.353 | 0.523 | 0.722 | 0.695 | 0.798 | 1.162 | 0.852 | 0.756 | 0.620 | 0.844 | |
| 17503 | hsa-miR-590-5p | 0.063 | 0.202 | 5.568 | 5.21E-01 | 7.31E-01 | 0.013 | 0.429 | 0.776 | -0.062 | -0.020 | 0.265 | 0.206 | 0.446 | 0.473 | -0.074 | -0.117 | 0.092 | |
| 17312 | hsa-miR-592 | -0.139 | 0.577 | 5.693 | 3.30E-01 | 5.70E-01 | 0.576 | 0.475 | 0.558 | 0.497 | 0.517 | 0.424 | 1.048 | 0.942 | 0.287 | 0.550 | 0.703 | 0.352 | |
| 42504 | hsa-miR-593-3p | 0.246 | 0.634 | 5.969 | 4.86E-02 | 2.45E-01 | 1.140 | 0.840 | 0.846 | 0.658 | 0.670 | 0.387 | 0.334 | 0.643 | 0.368 | 0.497 | 0.662 | 0.564 | |
| 17349 | hsa-miR-595 | -0.145 | 0.453 | 5.718 | 2.26E-01 | 4.45E-01 | 0.179 | 0.057 | 0.544 | 0.451 | 0.501 | 0.551 | 0.341 | 0.453 | 0.525 | 0.545 | 0.666 | 0.622 | |
| 17336 | hsa-miR-618 | 0.035 | 0.352 | 5.737 | 7.21E-01 | 8.54E-01 | 0.532 | 0.450 | 0.525 | 0.152 | 0.422 | 0.136 | 0.249 | 0.558 | 0.264 | 0.161 | 0.401 | 0.372 | |
| 46556 | hsa-miR-623 | 0.146 | 0.505 | 5.744 | 3.24E-01 | 5.69E-01 | 0.848 | 0.660 | 0.923 | 0.043 | 0.580 | 0.415 | 0.510 | 0.575 | 0.270 | 0.449 | 0.424 | 0.364 | |
| 17961 | hsa-miR-629-5p | 0.052 | 0.320 | 5.740 | 5.92E-01 | 7.66E-01 | 0.293 | 0.494 | 0.474 | 0.183 | 0.361 | 0.273 | 0.162 | 0.538 | 0.182 | 0.205 | 0.380 | 0.299 | |
| 42591 | hsa-miR-634 | 0.372 | -0.231 | 5.933 | 8.36E-03 | 1.78E-01 | 0.230 | -0.061 | 0.014 | -0.604 | -0.276 | 0.429 | 0.474 | -0.448 | -0.611 | -0.847 | -0.673 | -0.396 | |
| 169034 | hsa-miR-642b-5p | 0.222 | 0.307 | 6.732 | 1.70E-01 | 3.90E-01 | 1.235 | 0.127 | 0.196 | 0.335 | 0.202 | 0.410 | 0.620 | 0.166 | -0.113 | 0.233 | 0.036 | 0.234 | |
| 169375 | hsa-miR-660-3p | 0.363 | -0.572 | 5.586 | 1.02E-01 | 3.21E-01 | 0.885 | -0.813 | -0.771 | -0.681 | -0.537 | -0.429 | -0.070 | -0.862 | -1.055 | -0.902 | -1.021 | -0.614 | |
| 145973 | hsa-miR-664a-3p | 0.010 | 0.302 | 6.247 | 9.14E-01 | 9.55E-01 | 0.188 | 0.216 | 0.352 | 0.341 | 0.347 | 0.400 | 0.299 | 0.275 | 0.375 | 0.309 | 0.221 | 0.304 | |
| 168882 | hsa-miR-664b-3p | 0.229 | -0.531 | 7.364 | 1.40E-01 | 3.83E-01 | 0.044 | -0.873 | -0.274 | -0.797 | -0.505 | -0.093 | 0.120 | -1.012 | -0.578 | -0.670 | -0.917 | -0.814 | |
| 29490 | hsa-miR-7-5p | -0.064 | 0.150 | 8.433 | 6.64E-01 | 8.15E-01 | -0.800 | 0.735 | 1.102 | -0.607 | -0.404 | 0.681 | 0.660 | 0.495 | 0.828 | -0.425 | -0.452 | -0.016 | |
| 146064 | hsa-miR-718 | -0.204 | 0.860 | 6.170 | 1.49E-01 | 3.83E-01 | 0.551 | 0.647 | 0.792 | 1.132 | 0.841 | 0.586 | 1.108 | 1.310 | 0.909 | 0.929 | 0.763 | 0.753 | |
| 27568 | hsa-miR-744-5p | 0.212 | -0.542 | 5.664 | 1.65E-01 | 3.90E-01 | -0.781 | 0.464 | -0.181 | -0.547 | -0.717 | -0.850 | -0.547 | 0.273 | -1.022 | -0.495 | -1.023 | -1.072 | |
| 146111 | hsa-miR-767-5p | 0.134 | -0.211 | 6.323 | 2.11E-01 | 4.29E-01 | 0.013 | -0.143 | -0.104 | 0.063 | -0.166 | -0.531 | -0.425 | -0.127 | -0.407 | -0.020 | -0.381 | -0.310 | |
| 28884 | hsa-miR-876-3p | -0.083 | 0.656 | 5.702 | 4.65E-01 | 6.82E-01 | 0.884 | 0.566 | 0.803 | 0.375 | 0.533 | 0.527 | 0.838 | 0.791 | 0.547 | 0.504 | 0.772 | 0.734 | |
| 148622 | hsa-miR-877-3p | -0.062 | 0.443 | 6.093 | 5.37E-01 | 7.31E-01 | 0.405 | 0.327 | 0.456 | 0.551 | 0.457 | 0.275 | 0.492 | 0.568 | 0.322 | 0.491 | 0.539 | 0.429 | |
| 30033 | hsa-miR-877-5p | -0.092 | 0.341 | 6.142 | 5.74E-01 | 7.52E-01 | 0.186 | -0.151 | 0.610 | 0.647 | 0.100 | 0.381 | 0.480 | 0.654 | 0.036 | 0.493 | 0.255 | 0.405 | |
| 46259 | hsa-miR-885-5p | -0.176 | 0.596 | 5.863 | 1.46E-01 | 3.83E-01 | 0.605 | 0.592 | 0.762 | 0.157 | 0.517 | 0.416 | 0.791 | 0.848 | 0.552 | 0.618 | 0.626 | 0.668 | |
| 28047 | hsa-miR-890 | -0.325 | 0.942 | 5.856 | 6.69E-02 | 2.75E-01 | 1.154 | 0.214 | 1.000 | 0.735 | 0.789 | 0.784 | 1.295 | 1.324 | 1.171 | 1.027 | 0.882 | 0.929 | |
| 29852 | hsa-miR-9-3p | -0.155 | -0.111 | 5.274 | 4.28E-01 | 6.52E-01 | -0.259 | 0.416 | -0.530 | -0.560 | -0.737 | 0.537 | 1.779 | 0.096 | -0.367 | -0.420 | -0.725 | -0.567 | |
| 4040 | hsa-miR-9-5p | -0.143 | 0.757 | 6.248 | 2.46E-01 | 4.64E-01 | 0.216 | 1.218 | 0.549 | 0.321 | 0.296 | 1.517 | 1.979 | 1.261 | 0.242 | 0.604 | 0.395 | 0.492 | |
| 145693 | hsa-miR-92a-3p | 0.182 | -0.071 | 7.751 | 1.00E-01 | 3.21E-01 | 0.589 | -0.200 | 0.163 | -0.177 | 0.396 | -0.653 | -0.795 | -0.689 | -0.056 | -0.148 | 0.186 | 0.530 | |
| 30687 | hsa-miR-93-5p | 0.082 | -0.014 | 8.194 | 4.91E-01 | 7.00E-01 | -0.783 | 0.014 | 0.884 | -0.087 | 0.335 | -0.204 | -0.153 | -0.111 | 0.541 | -0.108 | -0.032 | -0.468 | |
| 42696 | hsa-miR-943 | 0.424 | 0.829 | 6.046 | 1.56E-02 | 1.78E-01 | 1.477 | 1.663 | 1.026 | 0.821 | 0.754 | 0.507 | 0.563 | 0.795 | 0.760 | 0.376 | 0.656 | 0.553 | |
| 13147 | hsa-miR-96-5p | 0.149 | 0.368 | 6.655 | 2.30E-01 | 4.49E-01 | -0.525 | 1.260 | 0.644 | 0.677 | 0.428 | 0.169 | 0.248 | 0.990 | 0.164 | 0.368 | 0.260 | -0.270 | |
| 11182 | hsa-miR-98-5p | -0.142 | 0.737 | 6.972 | 2.48E-01 | 4.65E-01 | 0.087 | 0.702 | 1.353 | 0.620 | 0.468 | 0.764 | 0.708 | 0.766 | 1.327 | 0.632 | 0.673 | 0.742 | |
| 17898 | hsa-miR-99b-3p | 0.107 | 0.551 | 5.810 | 2.60E-01 | 4.76E-01 | 0.596 | 0.664 | 0.782 | 0.457 | 0.614 | 0.516 | 0.385 | 0.617 | 0.515 | 0.418 | 0.557 | 0.496 | |
| 11184 | hsa-miR-99b-5p | 0.185 | -0.936 | 5.494 | 3.67E-01 | 5.87E-01 | -2.276 | 0.055 | 1.139 | -2.726 | -0.374 | -0.878 | -0.434 | -0.308 | 0.641 | -2.205 | -1.445 | -2.416 | |
| 28302 | hsa-miRPlus-A1015 | 0.256 | -0.439 | 6.791 | 5.68E-02 | 2.65E-01 | 0.232 | -0.432 | 0.029 | -0.529 | -0.394 | -0.773 | -0.615 | -0.560 | -0.352 | -0.758 | -0.658 | -0.459 | |
| 42492 | hsa-miRPlus-A1031 | 0.057 | 0.341 | 5.668 | 7.30E-01 | 8.54E-01 | 0.734 | 0.420 | 0.601 | 0.064 | 0.226 | 0.169 | 0.578 | 0.465 | -0.143 | 0.430 | 0.290 | 0.254 | |
| 17858 | hsa-miRPlus-A1073 | -0.002 | 0.882 | 6.151 | 9.83E-01 | 9.95E-01 | 0.944 | 0.844 | 0.982 | 0.871 | 0.941 | 0.706 | 0.780 | 0.931 | 0.883 | 0.881 | 0.898 | 0.927 | |
| 169416 | hsa-miRPlus-A1086 | -0.454 | -1.390 | 6.460 | 1.17E-01 | 3.46E-01 | -2.814 | -1.428 | -1.718 | 0.455 | -3.221 | -0.978 | -0.795 | -1.752 | -1.518 | 0.417 | -2.598 | -0.732 | |
| 17848 | hsa-miRPlus-A1087 | -0.141 | -1.003 | 6.720 | 1.85E-01 | 4.07E-01 | -1.102 | -1.417 | -1.360 | -0.152 | -1.565 | -0.843 | -0.892 | -1.288 | -1.284 | -0.138 | -1.283 | -0.708 | |
| 146113 | hsa-miRPlus-G1246-3p | -0.065 | -0.179 | 7.042 | 5.27E-01 | 7.31E-01 | -0.416 | -0.170 | -0.107 | -0.386 | -0.167 | -0.021 | 0.229 | -0.132 | -0.146 | -0.488 | -0.221 | -0.121 | |
| 147975 | hsa-miRPlus-J1003 | -0.145 | 0.561 | 5.673 | 3.43E-01 | 5.73E-01 | 0.670 | 0.378 | 0.457 | 0.433 | 0.546 | 0.443 | 0.648 | 1.294 | 0.515 | 0.464 | 0.369 | 0.509 | |
| 147576 | hsv1-miR-H1-3p | -0.220 | 0.446 | 5.882 | 6.09E-02 | 2.68E-01 | 0.225 | 0.205 | 0.525 | 0.141 | 0.549 | 0.375 | 0.505 | 0.650 | 0.600 | 0.533 | 0.490 | 0.557 | |
| 146058 | hsv1-miR-H3-3p | 0.324 | 0.379 | 5.682 | 3.84E-02 | 2.24E-01 | 1.024 | 0.709 | 0.919 | 0.012 | 0.360 | 0.223 | 0.123 | 0.287 | 0.111 | 0.175 | 0.204 | 0.406 | |
| 146117 | hsv1-miR-H6-3p | 0.250 | -0.033 | 6.176 | 3.61E-02 | 2.19E-01 | 0.556 | -0.010 | 0.195 | -0.207 | 0.102 | -0.081 | -0.058 | -0.275 | -0.152 | -0.269 | -0.246 | 0.054 | |
| 146090 | hsv1-miR-H7-3p | 0.248 | 0.145 | 5.845 | 6.90E-02 | 2.76E-01 | 0.880 | 0.275 | 0.301 | -0.153 | 0.257 | 0.052 | 0.187 | -0.090 | -0.015 | -0.108 | -0.057 | 0.205 | |
| 146042 | hsv1-miR-H8-3p | 0.059 | 0.351 | 6.092 | 5.27E-01 | 7.31E-01 | 0.405 | 0.450 | 0.541 | 0.226 | 0.330 | 0.334 | 0.345 | 0.330 | 0.330 | 0.288 | 0.273 | 0.365 | |
| 42656 | kshv-miR-K12-10a-3p | -0.199 | 0.641 | 6.033 | 7.06E-02 | 2.76E-01 | 0.425 | 0.291 | 0.418 | 0.693 | 0.723 | 0.697 | 0.704 | 0.759 | 0.549 | 0.897 | 0.819 | 0.712 | |
| 42624 | kshv-miR-K12-10b | -0.330 | 1.076 | 6.216 | 9.42E-03 | 1.78E-01 | 0.879 | 0.742 | 0.837 | 1.147 | 0.976 | 0.885 | 1.315 | 1.392 | 1.199 | 1.382 | 1.151 | 1.010 | |
| 19011 | SNORD10 | -0.074 | 0.124 | 6.544 | 5.33E-01 | 7.31E-01 | -0.435 | 0.043 | 0.266 | 0.322 | 0.310 | 0.017 | -0.025 | 0.111 | 0.183 | 0.357 | 0.186 | 0.156 | |
| 145666 | SNORD110 | 0.347 | 0.088 | 5.638 | 8.90E-03 | 1.78E-01 | 0.504 | 0.512 | 0.593 | -0.045 | 0.165 | -0.159 | -0.221 | 0.015 | -0.032 | -0.208 | -0.098 | 0.030 | |
| 19005 | SNORD118 | 0.226 | -0.430 | 5.623 | 8.81E-02 | 3.07E-01 | 0.223 | -0.520 | -0.570 | -0.184 | -0.070 | -0.783 | -1.086 | -0.517 | -0.551 | -0.209 | -0.413 | -0.482 | |
| 19606 | SNORD12 | 0.071 | 0.365 | 5.744 | 4.90E-01 | 7.00E-01 | 0.748 | 0.324 | 0.480 | 0.089 | 0.528 | 0.238 | 0.091 | 0.171 | 0.470 | 0.339 | 0.382 | 0.527 | |
| 19603 | SNORD13 | -0.060 | -0.161 | 6.990 | 6.18E-01 | 7.90E-01 | -0.543 | 0.400 | -0.496 | 0.376 | -0.410 | -0.473 | -0.729 | 0.359 | -0.495 | 0.394 | -0.331 | 0.016 | |
| 19607 | SNORD15A | 0.149 | 0.410 | 5.685 | 2.77E-01 | 5.06E-01 | 0.867 | 0.559 | 0.841 | 0.098 | 0.359 | 0.180 | 0.470 | 0.470 | 0.240 | 0.110 | 0.331 | 0.392 | |
| 19008 | SNORD2 | 0.184 | -0.393 | 7.705 | 1.05E-01 | 3.24E-01 | -0.139 | -0.387 | -0.067 | -0.511 | -0.197 | -0.501 | -0.529 | -0.567 | -0.532 | -0.491 | -0.576 | -0.214 | |
| 19007 | SNORD3@ | 0.343 | -1.162 | 9.584 | 4.88E-02 | 2.45E-01 | -0.257 | -0.409 | -0.956 | -1.205 | -0.827 | -2.289 | -2.239 | -0.784 | -1.131 | -1.111 | -1.435 | -1.300 | |
| 46204 | SNORD38B | -0.092 | -0.170 | 6.663 | 4.31E-01 | 6.52E-01 | -0.762 | -0.205 | -0.017 | -0.037 | -0.108 | -0.168 | -0.181 | -0.155 | -0.102 | -0.046 | -0.085 | -0.176 | |
| 46206 | SNORD44 | -0.140 | -0.259 | 6.893 | 4.45E-01 | 6.66E-01 | -1.380 | 0.240 | 0.063 | 0.105 | -0.425 | -0.575 | -0.362 | 0.132 | -0.093 | -0.137 | -0.458 | -0.214 | |
| 46205 | SNORD48 | -0.050 | -0.051 | 6.491 | 6.52E-01 | 8.14E-01 | -0.584 | -0.057 | 0.195 | 0.614 | -0.223 | -0.396 | -0.262 | -0.058 | 0.146 | 0.365 | -0.163 | -0.183 | |
| 46203 | SNORD49A | -0.119 | -0.417 | 7.029 | 4.15E-01 | 6.38E-01 | -1.268 | -0.554 | -0.292 | 0.032 | -0.036 | -0.738 | -0.713 | -0.426 | -0.383 | -0.005 | -0.209 | -0.409 | |
| 19604 | SNORD4A | 0.187 | -0.298 | 7.429 | 8.81E-02 | 3.07E-01 | -0.016 | -0.222 | -0.062 | -0.328 | 0.040 | -0.638 | -0.549 | -0.459 | -0.391 | -0.393 | -0.304 | -0.251 | |
| 19605 | SNORD6 | 0.224 | -0.526 | 7.761 | 1.45E-01 | 3.83E-01 | 0.227 | -1.086 | -0.250 | -0.806 | -0.123 | -0.449 | -0.379 | -1.059 | -0.462 | -0.776 | -0.432 | -0.719 | |
| 145663 | SNORD68 | 0.441 | -0.586 | 9.383 | 1.89E-02 | 1.78E-01 | 0.069 | -0.323 | -0.409 | -0.869 | -0.086 | -0.576 | -0.540 | -0.770 | -0.785 | -0.814 | -0.983 | -0.951 | |
| 17492 | sv40-miR-S1-5p | 0.223 | -0.325 | 5.970 | 1.04E-01 | 3.24E-01 | 0.764 | -0.771 | -0.424 | -0.602 | -0.120 | -0.126 | -0.125 | -0.595 | -0.894 | -0.624 | -0.622 | 0.244 | |

**Supplementary Table S3. Differentially expressed miRNAs under hypoxic conditions.** Significant differentially expressed miRNAs under hypoxia determined by multivariate analysis. A p-value <0.05 was considered significant and miRNAs are ranked according to significance.


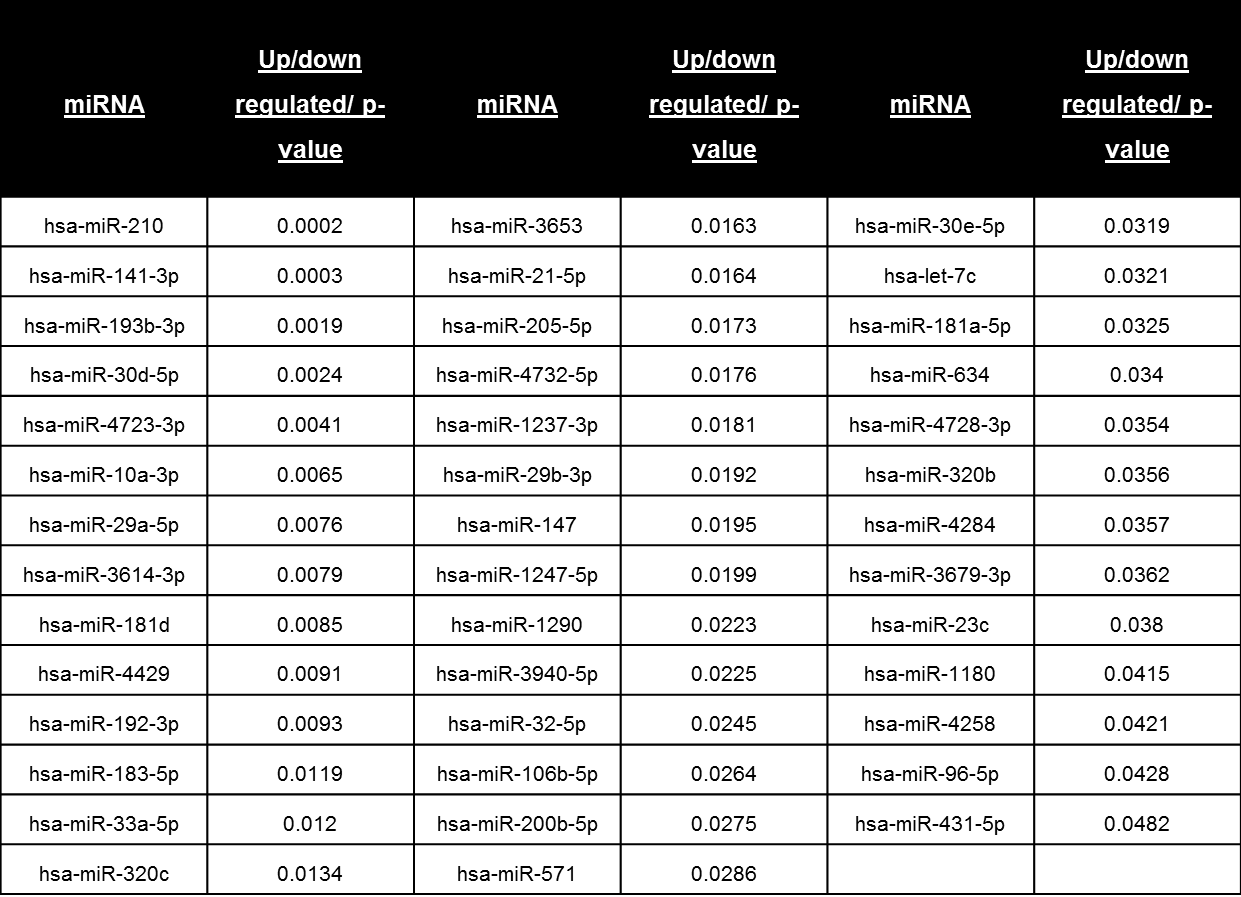


**Supplementary Table S4. Metabolites with concentrations altered by hypoxia.** Metabolites identified after linear modelling following fitting for cell line and oxygen tension. All significant metabolites are shown (<0.05) and are rank with the most significant first.

| KEGG ID | Name | P-value | KEGG ID | Name | P-value |
| --- | --- | --- | --- | --- | --- |
| C00041 | Ala | <.0001 | **C00248** | alpha-Lipoamide+5-Methoxyindoleacetate | 0.0015 |
| C00188 | Thr | <.0001 | **C00354** | F1,6P | 0.0015 |
| C00075 | UTP | <.0001 | **C00025** | Glu | 0.0021 |
| C00049 | Asp | <.0001 | **C00270** | N-Acetylneuraminate | 0.0023 |
| C00167 | UDP-glucuronate | <.0001 | **C00315** | Spermidine | 0.0028 |
| C00079 | Phe | <.0001 | **C00127** | Glutathione(ox) | 0.0034 |
| C00082 | Tyr | <.0001 | **C00631** | 2PG | 0.0036 |
| C00135 | His | <.0001 | **C03406** | Argininosuccinate | 0.0037 |
| C00051 | Glutathione(red) | <.0001 | **C00003** | NAD+ | 0.0044 |
| C00063 | CTP | <.0001 | **C01620** | Threonate | 0.0049 |
| C00233 | 4-Methyl-2-oxopentanoate | <.0001 | **C00065** | Ser | 0.005 |
| C01015 | Hydroxyproline | <.0001 | **C03793** | N6,N6,N6-Trimethyllysine | 0.0052 |
| C00029 | UDP-glucose | <.0001 | **C00024** | Acetyl CoA | 0.0053 |
| C02291 | Cystathionine | <.0001 | **C03626** | ADMA | 0.0062 |
| C02356 | 2AB | <.0001 | **C01089** | 3-Hydroxybutyrate | 0.0075 |
| C00141 | 2-Oxoisopentanoate | <.0001 | **C00019** | SAM+ | 0.0081 |
| C00670 | Glycerophosphorylcholine | 0.0001 | **-** | 2-Hydroxypentanoate | 0.0098 |
| C00015 | UDP | 0.0001 | **C01585** | Hexanoate | 0.0098 |
| C00092 | G6P | 0.0002 | **C00383** | Malonate | 0.0109 |
| C00186 | Lactate | 0.0002 | **C00489** | Glutarate | 0.0114 |
| C01152 | 3-Methylhistidine | 0.0002 | **C00044** | GTP | 0.0119 |
| C00864 | Pantothenate | 0.0002 | **C00005** | NADPH | 0.0123 |
| C00624 | N-Acetylglutamate | 0.0003 | **C00345** | 6-Phosphogluconate | 0.0125 |
| C00183 | Val | 0.0004 | **C00407** | Ile | 0.0128 |
| C00152 | Asn | 0.0005 | **C01601** | Pelargonate | 0.0132 |
| C00346 | Ethanolamine phosphate | 0.0005 | **C00214** | Thymidine | 0.015 |
| C00158 | Citrate | 0.0006 | **C00334** | GABA | 0.0151 |
| C00803 | Pentanoate | 0.0006 | **C02989** | Methionine sulfoxide | 0.0164 |
| C00750 | Spermine | 0.0008 | **C00037** | Gly | 0.0166 |
| C00085 | F6P | 0.0008 | **C00147** | Adenine | 0.0177 |
| C00093 | Glycerophosphate | 0.0009 | **C00111** | DHAP | 0.0179 |
| C00134 | Putrescine(1,4-Butanediamine) | 0.0009 | **C00213** | Sarcosine | 0.0206 |
| C00148 | Pro | 0.0009 | **C00170** | 5-Methylthioadenosine | 0.0211 |
| C00197 | 3PG | 0.0009 | **C00086** | Urea | 0.0217 |
| C05382 | S7P | 0.001 | **C08261** | Azelate | 0.0222 |
| C00021 | SAH | 0.001 | **C02571** | o-Acetylcarnitine | 0.0234 |
| C00020 | AMP | 0.0012 | **C01042** | N-Acetylaspartate | 0.0246 |
| C00103 | G1P | 0.0012 | **C00328** | Kynurenine | 0.0252 |
| C00248 | alpha-Lipoamide+5-Methoxyindoleacetate | 0.0015 | **C06104** | Adipate | 0.0261 |
| C00354 | F1,6P | 0.0015 | **C00612** | N1-Acetylspermidine | 0.0275 |
| C00025 | Glu | 0.0021 | **C00430** | 5-Aminolevulinate | 0.0294 |
| C00270 | N-Acetylneuraminate | 0.0023 | **C06423** | Octanoate | 0.0295 |
| C00315 | Spermidine | 0.0028 | **C00212** | Adenosine | 0.0296 |
| C00127 | Glutathione(ox) | 0.0034 | **C03761** | 3-Hydroxy-3-methylglutarate | 0.0319 |

**Supplementary Table S5. Clinicopathological features of patients undergoing adjuvant 5-fluorouracil treatment for colorectal cancer.**

| **Code** | **Group** | **Stage** | **Age** | **Survival (yrs)** | **TNM** |
| --- | --- | --- | --- | --- | --- |
| 11 | 0 | C | 64 | 9.4 | T3N1M0 |
| 12 | 0 | C | 58 | 8.7 | T3N1M0 |
| 17 | 0 | C | 30 | 7.0 | T3N1M0 |
| 21 | 0 | C | 67 | 7.5 | T2N1M0 |
| 22 | 0 | C | 74 | 7.6 | T2N1M0 |
| 34 | 0 | B | 42 | 7.3 | T4N0M0 |
| 14 | 1 | C | 73 | 0.7 | T3N1M0 |
| 15 | 1 | C | 77 | 3.4 | T4N1M0 |
| 20 | 1 | B | 75 | 3.7 | T4N1M0 |
| 30 | 1 | C | 61 | 1.4 | T4N2M0 |
| 31 | 1 | C | 72 | 2.2 | T3N1M0 |

**Supplementary Figure legends**

**Supplementary Figure S1. PLS-DA axis 6 loadings.** Log transformed miRNA data was subjected to unsupervised PLS-DA analysis. Loadings from PLS-DA axis 6 for each of the miRNAs are shown. Rectangular boxes outline the location of the bar graphs below.

**
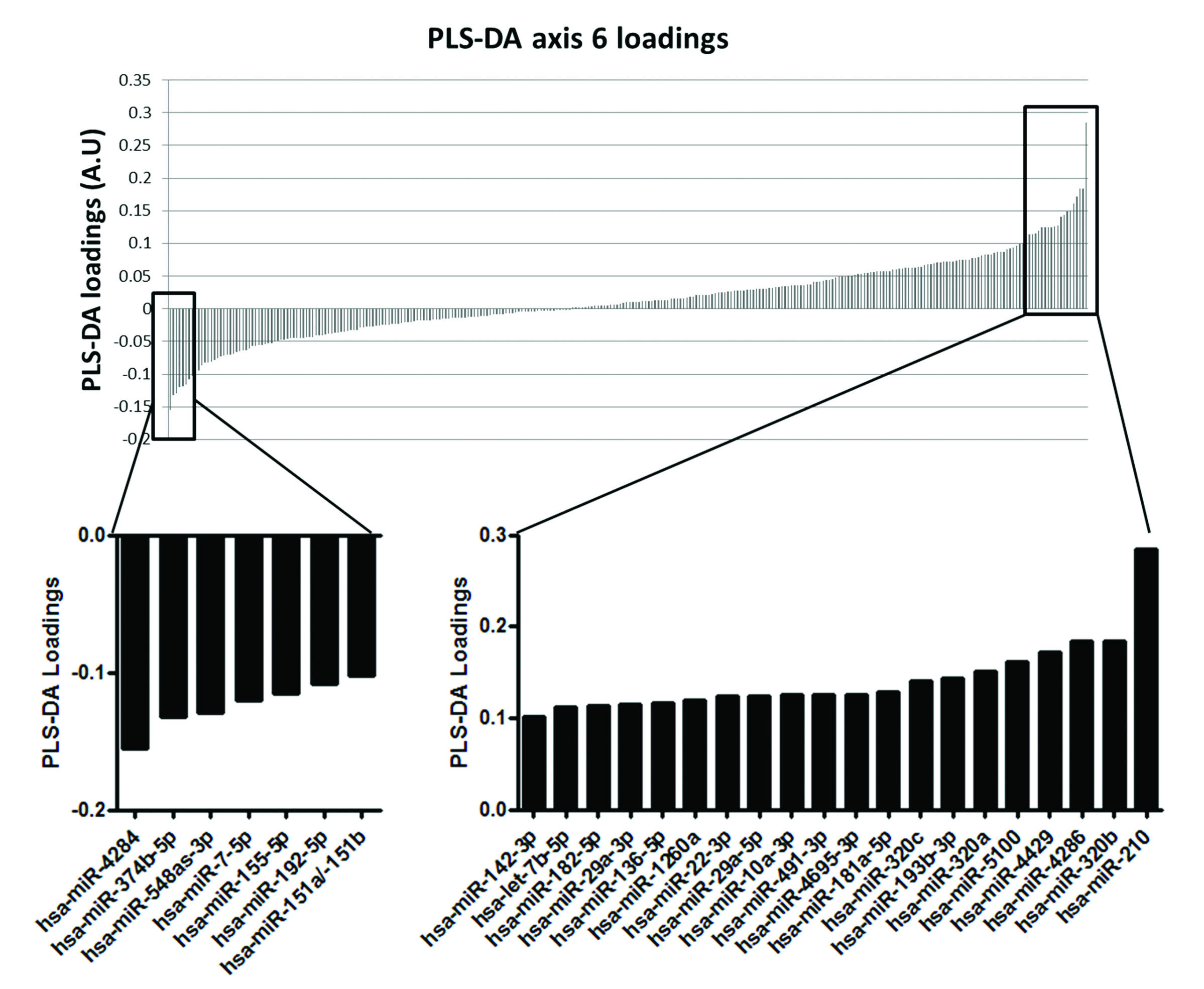
**

**Supplementary Figure S2. Lactate levels are increased in colorectal cancer cells held in hypoxia.** Bars represent mean values with SEM calculated from all six colorectal cell lines ***p<0.001.


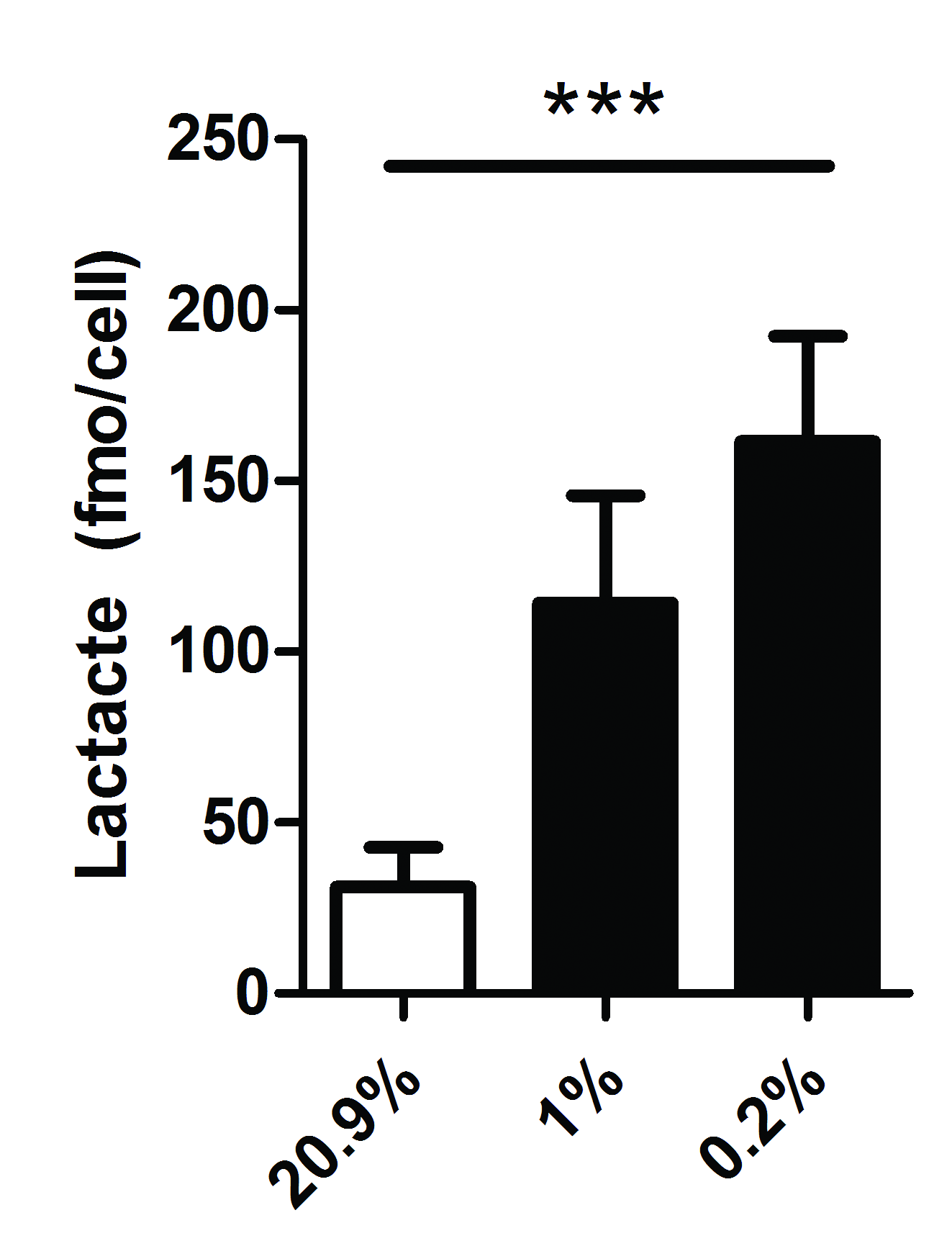


**Supplementary Figure S3. Cell survival following various doses of 5-FU treatment of DLD-1 cells**. Cells were cultured under two oxygen conditions (normoxia *versus* hypoxia (0.2%) expressed as a percentage of cells without treatment (black bar).


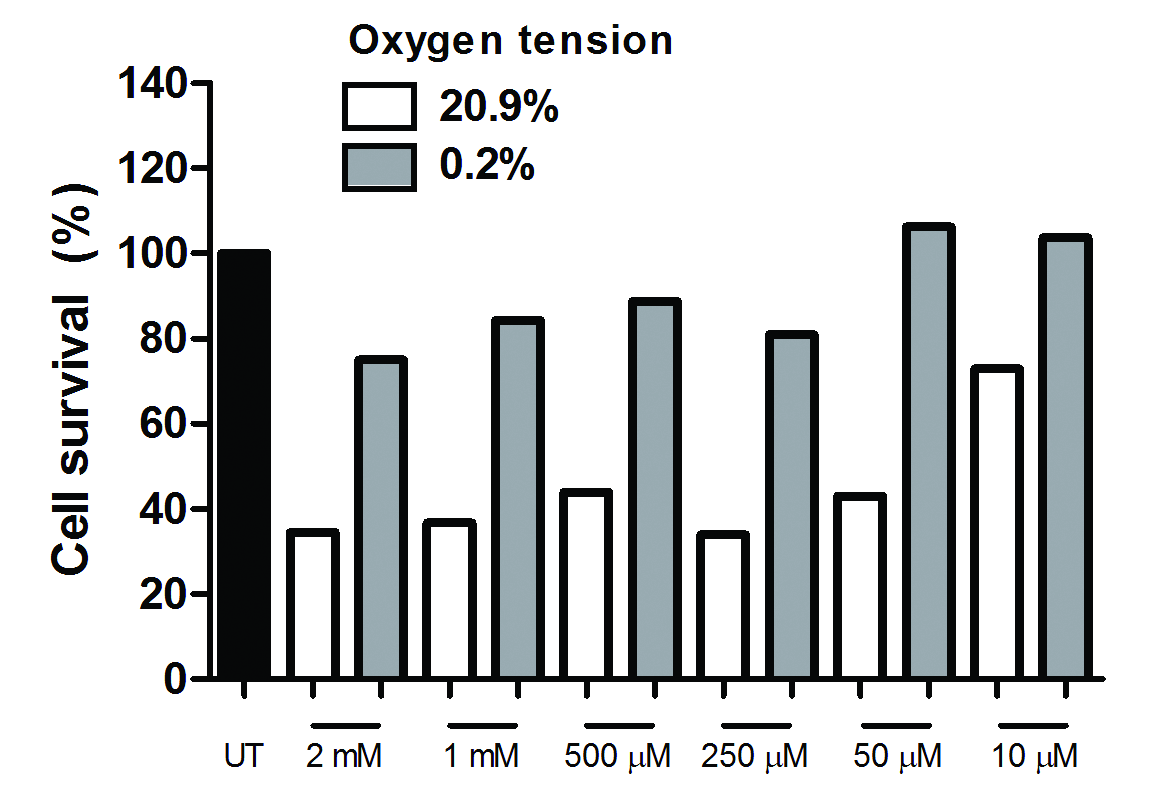

Supplement: Supplementary Data [file ddx059_Supp.docx]
